# Continuum tuning of nanoparticle interfacial properties by dynamic covalent exchange†
†Electronic supplementary information (ESI) available: Experimental procedures; characterization data for all compounds and nanoparticles; supplementary TEM images; NP size distributions (TEM); cluster size distributions (DLS). See DOI: 10.1039/c7sc03666c


**DOI:** 10.1039/c7sc03666c

**Published:** 2017-11-17

**Authors:** William Edwards, Nicolas Marro, Grace Turner, Euan R. Kay

**Affiliations:** a EaStCHEM School of Chemistry , University of St Andrews , North Haugh, St Andrews , KY16 9ST , UK . Email: ek28@st-andrews.ac.uk

## Abstract

Surface chemical composition is fundamental to determining properties on the nanoscale, making precise control over surface chemistry critical to being able to optimise nanomaterials for virtually any application. Surface-engineering independent of the preparation of the underlying nanomaterial is particularly attractive for efficient, divergent synthetic strategies, and for the potential to create reactive, responsive and smart nanodevices. For monolayer-stabilised nanoparticles, established methods include ligand exchange to replace the ligand shell in its entirety, encapsulation with amphiphilic (macro)molecules, noncovalent interactions with surface-bound biomolecules, or a relatively limited number of covalent bond forming reactions. Yet, each of these approaches has considerable drawbacks. Here we show that dynamic covalent exchange at the periphery of the nanoparticle-stabilizing monolayer allows surface-bound ligand molecular structure to be substantially modified in mild and reversible processes that are independent of the nanoparticle–molecule interface. Simple stoichiometric variation allows the extent of exchange to be controlled, generating a range of kinetically stable mixed-monolayer compositions across an otherwise identical, self-consistent series of nanoparticles. This approach can be used to modulate nanoparticle properties that are defined by the monolayer composition. We demonstrate switching of nanoparticle solvent compatibility between widely differing solvents – spanning hexane to water – and the ability to tune solubility across the entire continuum between these extremes, all from a single nanoparticle starting point. We also demonstrate that fine control over mixed-monolayer composition influences the assembly of discrete, colloidally stable nanoparticle clusters. By carefully assessing monolayer composition in each state, using both *in situ* and *ex situ* methods, we are able to correlate the molecular-level details of the nanoparticle-bound monolayer with system-level properties and behaviour. These empirically determined relationships contribute fundamental insights on nanoscale structure–function relationships, which are currently beyond the capabilities of *ab initio* prediction.

## Abstract 2

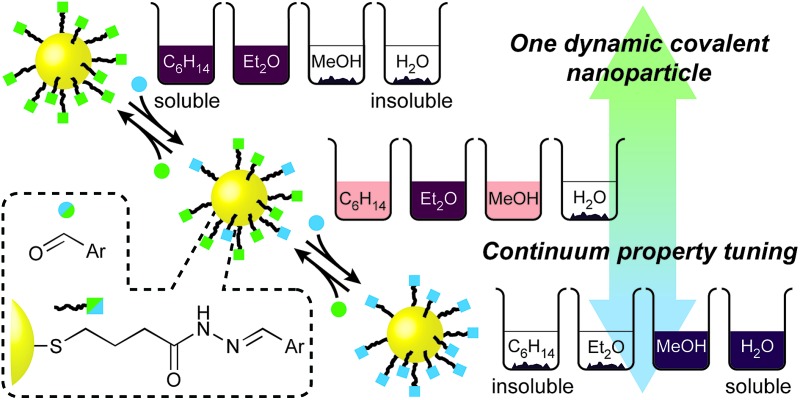
Dynamic covalent modification of the surface-stabilizing monolayer accesses a continuum of nanoparticle properties from a single starting point.

## Introduction

The remarkable and often unique behaviours displayed by nanoparticles suggest myriad potential applications, from optoelectronics to catalysis; sensing to biolabelling.^1^ Many nanoparticle characteristics are determined primarily by the chemical composition, size and shape of the core. Yet, a whole host of physicochemical properties and interactions with exogenous species are governed by the interface between the nanoparticle and its surrounding matrix. Control over the characteristics and composition of the surface-bound ligand shell is therefore crucial to defining nanoparticle behaviour and achieving success in any application.^2^

It has recently been demonstrated that dynamic covalent exchange reactions at the periphery of nanoparticle-stabilizing monolayers offer a powerful strategy for manipulating molecular structure of the ligand shell,^3^ thus achieving adaptive surface functionalization,^4^,^5^ switchable physicochemical properties,^4^ or assembly and disassembly of covalently linked assemblies.^6^ We therefore hypothesized that dynamic covalent exchange could be used to achieve continuum tuning of monolayer-related properties within an otherwise structurally and chemically identical series, generated from a single nanoparticle starting point.

From a technological standpoint, solution processability is one of the defining characteristics of monolayer-stabilized colloidal nanoparticles, allowing manipulation using established (macro)molecular techniques and infrastructure. Meanwhile, any solution-based application demands that nanoparticle solvent compatibility be optimized for the target environment. Yet, very few studies have examined the relationships between monolayer features and nanoparticle colloidal stability in detail. A recent report quantitatively delineated the enthalpic and entropic thermodynamic factors that govern nanoparticle solubility, and confirmed that appropriate molecular-level design of the ligand shell can greatly enhance performance of nanoparticle-based devices both in solution and in the solid state.^7^

Mixed-ligand monolayers that combine hydrophobic and hydrophilic ligands are receiving increasing attention for generating nanoparticles of differing solvophilicities.^8^ Nanoparticle solubility, aggregation, and interactions with interfaces are each highly sensitive to the monolayer composition (*i.e.* ligand molecular structure and relative stoichiometry), and consequential effects including the hierarchical arrangement of surface-bound components.‡
‡Environment-responsive or spontaneous phase-separation in mixed monolayers is sometimes observed for ligands of markedly differing structures; both experiment and theory provide evidence that such hierarchical morphology parameters can be important in determining nanoparticle solubility (ref. 8*b*, *h*, 9*a*, *c* and *i*), self-assembly (ref. 8*g* and 9*e*), assembly at interfaces (ref. 8*a*, *e*, 9*g* and *j*), and interactions with biomembranes (ref. 9*b*, *d*, *f*, *h* and *k*).
8,9 Yet, it remains a considerable challenge to produce mixed-ligand monolayers with predictable and verifiable compositions across a self-consistent series of nanoparticles; meanwhile dynamic switching of ligand-shell compositions – and therefore between nanoparticle property states – has not been demonstrated.

Reliable methods for manipulating the ligand shell are therefore essential for practical engineering of nanoparticle surface functionalization and associated properties.^2^ Established strategies for post-synthesis nanoparticle solubility tuning or phase transfer are dominated by ligand exchange to replace the ligand shell in its entirety, encapsulation in micelles or with amphiphilic polymers, formation of noncovalent host–guest complexes, or a relatively limited number of covalent bond forming reactions.^2^,^10^ Ligand exchange is often essentially irreversible and can lead to surface defects or reconstruction.2a,d,^10^,^11^ Although this is the method most commonly employed to produce mixed-ligand monolayers, nonlinear relationships between the ligand composition introduced and the final nanoparticle-bound ratio are frequently observed, and it can be challenging to access all monolayer compositions.8h,9j,^12^ Encapsulation approaches typically achieve kinetic stability at the expense of reversibility,2a,^10^,^13^ while the attendant increase in nanostructure size, and masking of the core material, can each be deleterious to application performance. On the other hand, host–guest complexes tend to be kinetically labile and therefore intrinsically concentration sensitive; commonly an excess of the noncovalent modifier is required, and there is no simple way to reverse the property change.^14^

**Fig. 1:**
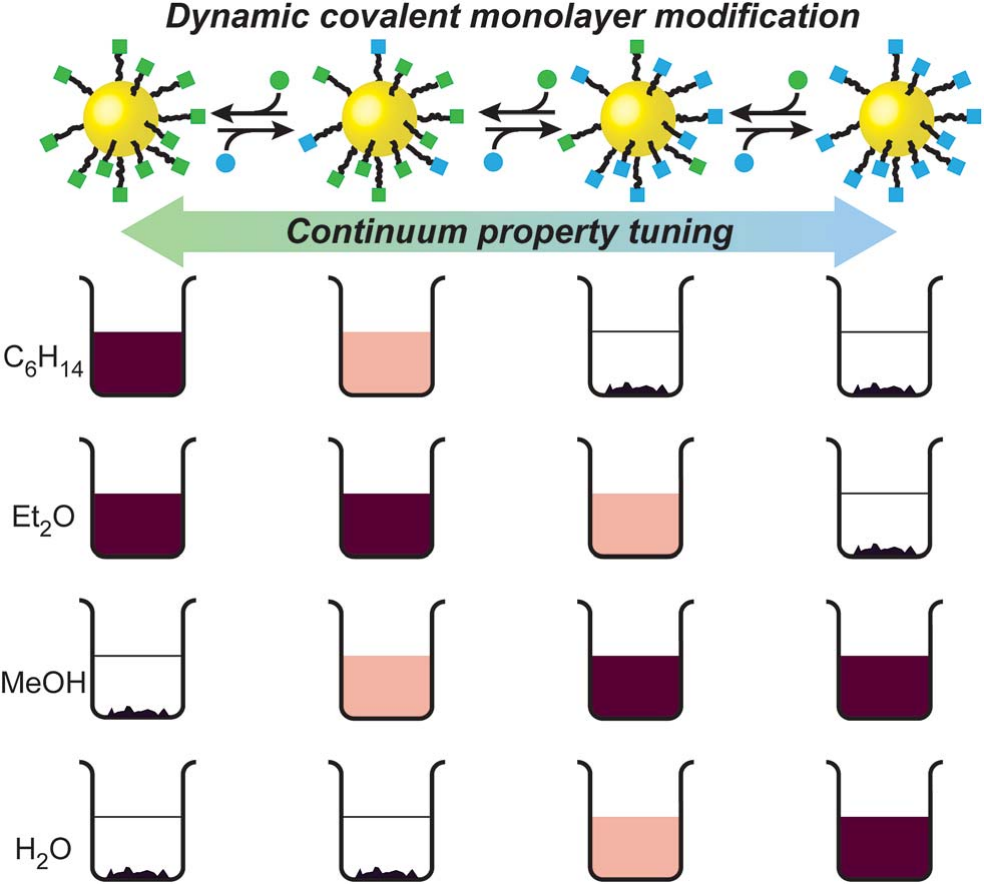
The dynamic covalent approach to reversible nanoparticle property tuning. From a single, synthetically optimised starting point, dynamic covalent modification gives access to multiple single-component or mixed-component nanoparticle-bound monolayer compositions, and hence, nanoparticle property states; for example, tuning nanoparticle solubility, illustrated schematically here as depth of magenta colour for nanoparticle solutions in a range of solvents.

Here, we show that dynamic covalent exchange provides a rapid and simple method to vary nanoparticle-bound ligand shell composition, and therefore nanoparticle properties (Fig. 1). Complete conversion of one single-component monolayer into another may be achieved, or any number of binary mixed-ligand compositions between these two extremes generated. We use this method to tune nanoparticle solubility properties across a continuum of states ranging from excellent solubility in hexane through to water, all from a single, synthetically optimized, nanoparticle starting point. The processes are entirely reversible, require neither disruption nor masking of the ligand shell, and each state corresponds to an easily isolable, all-covalent, kinetically stable entity. It is therefore possible to establish quantitative correlations between monolayer composition and nanoparticle solubility, which would not be predictable *ab initio*. Furthermore, we demonstrate that this approach can be extended to vary other properties associated with the nanoparticle–environment interface, specifically solvent- and monolayer-composition-dependent nanoparticle clustering. This study exemplifies the power of dynamic covalent exchange reactions as a general method for precisely engineering nanoparticle surface chemical composition, through which fine control over a whole host of properties may be achieved.

## Nanoparticle preparation and solubility switching

**Fig. 2:**
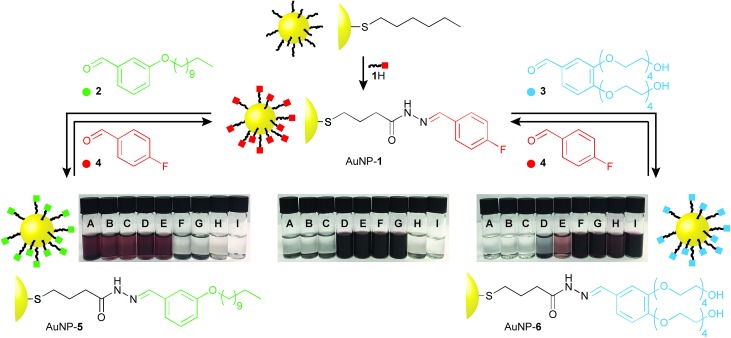
Reversible switching of nanoparticle solvophilicity between three states by dynamic covalent hydrazone exchange from a single nanoparticle starting point AuNP-**1**. Dynamic covalent exchange conditions: aldehyde **2**, **3**, or **4** (5 equivalents with respect to nanoparticle-bound ligand), THF/CH_2_Cl_2_/D_2_O (interconversions between AuNP-**1** and AuNP-**5**) or THF/D_2_O (interconversions between AuNP-**1** and AuNP-**6**), CF_3_CO_2_H (20 mM), 50 °C. Digital photographs of nanoparticles at 0.5 mg mL^–1^ in solvents: (A) *n*-hexane; (B) CCl_4_; (C) Et_2_O; (D) tetrahydrofuran (THF); (E) CH_2_Cl_2_; (F) *N*,*N*-dimethylformamide (DMF); (G) (CH_3_)_2_SO; (H) EtOH; (I) H_2_O.

The dynamic covalent reactivity of hydrazones provides an attractive balance of kinetic lability under acid catalysed conditions, and long-term stability on separation from the catalyst.^4^,5b,^15^ In order to maximise the effect of dynamic covalent exchange on nanoparticle physicochemical properties, we designed ligand **1**H (Fig. 2 and Scheme S1†), which has a simplified structure and lower molecular weight compared to previously reported dynamic covalent nanoparticle ligand structures.^4^,^5^ An *N*-acyl hydrazone is connected through a short hydrocarbon linker to a terminal sulfur, which allows binding to AuNP surfaces *via* the strong thiyl–gold interaction.^16^ The 4-fluorobenzylidine derivative was chosen to allow for accurate assessment of monolayer structure and composition by ^19^F NMR.^4^,^6^ Ligand exchange from a hexanethiyl-protected AuNP precursor (Scheme S1†), followed by purification from all unbound molecular species by nanoparticle precipitation, washing and re-dispersion, afforded AuNP-**1** (*d* ≈ 3.8 nm). Monolayer molecular structure, and purity from unbound molecular species could be verified by ^1^H and ^19^F NMR analyses (Fig. S3†).

Nanoparticle solubility was qualitatively assessed by adding solvent to dried samples at a ratio of 0.5 mg mL^–1^, followed by ultrasonication for 10 minutes, then sedimentation of undissolved solids by centrifugation. On visual inspection (Fig. 2) AuNP-**1** exhibited excellent solubility in organic solvents of intermediate polarity (tetrahydrofuran (THF) and CH_2_Cl_2_) as well as in polar aprotic solvents (*N*,*N*-dimethylformamide (DMF) and (CH_3_)_2_SO), while being completely insoluble in both apolar organic solvents (*e.g. n*-hexane, CCl_4_, Et_2_O) or water. These nanoparticles were also entirely insoluble in EtOH. This solubility behaviour can be correlated with empirical measures of solvent polarity, appearing to align most closely with the *E*_T_(30) parameter (see ESI, Section 6†), and is consistent with AuNP-**1** presenting a solvent-exposed surface that is compatible with environments of intermediate polarity.

Nanoparticle solubility properties could be altered by dynamic covalent hydrazone exchange with simple aldehyde modifiers, each chosen to introduce significantly different solvophilicities at the monolayer periphery: oleophilic, alkyl-modified **2**; or hydrophilic tetraethylene glycol appended **3** (Fig. 2). Treating AuNP-**1** with either **2** or **3**, in the presence of an acid catalyst (CF_3_CO_2_H) and a small quantity of water triggered the hydrazone exchange reaction. Monitoring *in situ* by ^19^F NMR in the presence of an internal standard, the broad signal for nanoparticle-bound 4-fluorobenzylidine hydrazone **1** was observed to decrease in intensity, concomitant with appearance of a sharp signal for released 4-fluorobenzaldehyde (**4**), allowing simultaneous determination of the concentration of both species (Fig. S4†). The dramatic induced change in nanoparticle solvent compatibility demanded careful choice of reaction solvent. Initial attempts in THF/D_2_O (9 : 1) led to nanoparticle precipitation after several hours, consistent with a significant change in monolayer solvophilicity, but arresting the exchange process before complete conversion was achieved. In improved procedures, AuNP-**1** was treated with 5 equivalents (with respect to the concentration of ligand **1**) aldehyde **2** in a solvent mixture of decreasing polarity (THF/CH_2_Cl_2_/D_2_O 18 : 4 : 1 → 18 : 6 : 1) to maintain a stable nanoparticle dispersion, allowing quantitative exchange of AuNP-**1** into AuNP-**5**. Similarly, treating AuNP-**1** with 5 equivalents **3** in a solvent mixture of increasing polarity (THF/D_2_O 9 : 1 → 3 : 1) smoothly produced AuNP-**6**. In each case, adding a further 5 equivalents aldehyde **2** or **3** induced no further release of **4**, the concentration of which matched the concentration of nanoparticle-bound **1** estimated at the start of the experiment (Fig. S4 and S7†). Pure samples of both AuNP-**5** and AuNP-**6** were isolated by several cycles of precipitation and washing with non-solvents. In both cases, ^1^H and ^19^F analyses indicated complete conversion of the 4-fluorobenzylidine species stabilizing AuNP-**1** (Fig. S5 and S8†). This was further verified by oxidative stripping of alkylthiyls from the nanoparticle surface using iodine. Subsequent NMR analysis of all released species in bulk solution by ^19^F and ^1^H NMR revealed only signals corresponding to the newly generated nanoparticle-bound ligands (**5** or **6**), with no indication of any remaining ligand **1** (Fig. S6 and S9†).

On isolation, it was immediately apparent that the nanoparticle solubility properties had changed dramatically (Fig. 2). AuNP-**5** displayed excellent solubility in solvents of low to medium polarity (*e.g.*, *n*-hexane, CCl_4_, Et_2_O, THF and CH_2_Cl_2_), but were completely insoluble in solvents of higher polarity, including polar aprotic solvents, which we commonly find to be excellent for other nanoparticles bearing similar monolayer designs. On the other hand, AuNP-**6** were insoluble in apolar solvents (*n*-hexane, CCl_4_ or Et_2_O), were only marginally soluble in THF or CH_2_Cl_2_, but showed excellent solubility in polar aprotic solvents (DMF, (CH_3_)_2_SO), alcohols (EtOH), and even water. High saturation concentrations were achieved in ‘good’ solvents for each nanoparticle sample: AuNP-**1** in THF 34 mg mL^–1^; AuNP-**1** in DMF 41 mg mL^–1^; AuNP-**5** in *n*-hexane 35 mg mL^–1^; AuNP-**6** in water 22 mg mL^–1^.

Purified samples of either AuNP-**5** or AuNP-**6** could be reconverted back to AuNP-**1**, simply by subjecting them to the same hydrazone exchange conditions in the presence of an excess of aldehyde **4**. Thus, from the one nanoparticle starting point, excellent solubility can be attained in solvents spanning the full range of polarity form *n*-hexane to water, using only three readily interchangeable components to modify a compact surface monolayer. These substantial transformations are achieved using only structurally simple and charge-neutral nanoparticle-bound ligand structures, and do not require addition of polymeric modifiers or other surfactants. In contrast to ligand exchange, quantitative conversion of one single-component monolayer into another is readily achieved, the process is entirely reversible, and the crucial nanoparticle–ligand bond is unaffected, suggesting that this strategy should be generalizable across a range of underlying nanomaterials.

## Continuum tuning of nanoparticle solubility

We hypothesised that a finer degree of property control could be achieved by creating mixed monolayers to access solubility states intermediate between the extremes defined by AuNP-**1**, AuNP-**5** and AuNP-**6**. Binary mixed-ligand compositions were readily produced by treating AuNP-**1** with differing stoichiometric quantities of aldehyde modifiers **2** or **3** (Tables 1 and 2). Once the reaction endpoint had been reached, the nanoparticle products were purified by precipitation and resuspension (see ESI, Sections 4 and 5† for full experimental details). Again, the concentration of aldehyde **4** released during the exchange reaction (as determined by *in situ*^19^F NMR *versus* an internal standard) gave an initial estimation of the monolayer composition. We sought to corroborate this for the purified nanoparticle products by direct observation of both surface-bound ligands. The lack of a fluorine substituent in **5** or **6** hampers accurate quantification of these species by NMR when bound to the nanoparticle surface. However, for the series of samples AuNP-**1**_*x*_**5**_*y*_, oxidative decomposition using iodine allowed the ratio of ligand components to be measured in bulk solution by ^1^H NMR (Fig. S10†), giving excellent agreement with the results from the *in situ* pre-purification analysis (Table 1). The difference between the monolayer composition measured by each method is within the error of the measurement techniques, and importantly, the quantity of aldehyde **4** released during the dynamic covalent exchange is never greater than the amount of hydrazone **5** incorporated within the new monolayer,§
§The fact that any small discrepancies between the two measurements always show less **4** released during the reaction than **2** incorporated within the product monolayer may be the result of a small systematic error in estimating the initial concentration of AuNP-**1**, or may instead indicate preferential selection for more hydrophobic nanoparticles (with higher **5** : **1** ratios) during purification. consistent with quantitative exchange between surface-bound hydrazones, leaving negligible free hydrazide.¶
¶This is consistent with the fact that in this study and others, we commonly observe that hydrazones of the general structure of **1**/**5**/**6** are relatively stable with respect to hydrolysis (whether nanoparticle-bound or not). As expected, the extent of hydrazone exchange was dependent on the quantity of aldehyde **2** introduced, and varying binary mixed-ligand compositions spanning pure AuNP-**1** to AuNP-**1**_0.1_**5**_0.9_ could be accessed by adding between 0.26 and 3.1 equivalents **2**, under otherwise identical conditions (Tables 1 and S1†). Increasing the excess of **2** beyond this value led to no further increase in the monolayer mole fraction of **5** (*χ***_5_**) under these conditions as a result of the poor solubility of AuNP-**1**_0.1_**5**_0.9_ in the 10% D_2_O/THF solvent mixture; quantitative exchange could only be achieved on re-solubilization by addition of CH_2_Cl_2_ (*vide supra*). Although now markedly different at the molecular level in terms of monolayer composition, and in spite of the rigorous purification procedures performed, all samples exhibited very similar nanoscale characteristics in terms of shape and size distributions (see ESI, Section 9†).

**Table 1:** Continuum tuning of binary mixed-monolayer compositions of components **1** and **5** (for full experimental details and sample characterization, see ESI Sections 4, 5, and Table S1)

Sample	Equivalents **2** added^*a*^	% **4** released^*b*^	*χ* **_5_** ^*c*^
AuNP-**1**_0.7_**5**_0.3_	0.26	29%	0.29
AuNP-**1**_0.6_**5**_0.4_	0.52	37%	0.41
AuNP-**1**_0.5_**5**_0.5_	0.54	46%	0.50
AuNP-**1**_0.4_**5**_0.6_	0.80	56%	0.61
AuNP-**1**_0.3_**5**_0.7_	1.1	68%	0.71
AuNP-**1**_0.2_**5**_0.8_	2.1	79%	0.78
AuNP-**1**_0.1_**5**_0.9_-a	3.1	89%^*d*^	0.88
AuNP-**1**_0.1_**5**_0.9_-b	4.1	85%^*d*^	0.89
AuNP-**1**_0.1_**5**_0.9_-c	5.2	89%^*d*^	0.89
AuNP-**5**	5.1	100%^*e*^	>0.97

^*a*^Molar equivalents with respect to initial concentration of nanoparticle-bound ligand **1**.

^*b*^Determined by *in situ*^19^F NMR.

^*c*^Determined by oxidative ligand stripping using I_2_ (see ESI, Section 5).

^*d*^Nanoparticle precipitation observed.

^*e*^Dynamic covalent hydrazone exchange driven to completion by increasing the proportion of CH_2_Cl_2_ to maintain nanoparticle solubility.

**Table 2:** Continuum tuning of binary mixed-monolayer compositions of components **1** and **6** (for full experimental details and sample characterization, see ESI Sections 4, 5, and Table S2)

Sample	Equivalents **3** added^*a*^ ^,^‖	% **4** released^*b*^	*χ* **_6_** ^*c*^
AuNP-**1**_0.9_**6**_0.1_	0.12	n.d.	0.10
AuNP-**1**_0.8_**6**_0.2_^*d*^	0.50	n.d.	0.20
AuNP-**1**_0.7_**6**_0.3_	0.29	26%	0.28
AuNP-**1**_0.6_**6**_0.4_	0.47	45%	0.43
AuNP-**1**_0.5_**6**_0.5_-a^*d*^	1.0	n.d.	0.46
AuNP-**1**_0.5_**6**_0.5_-b	0.67	52%	0.51
AuNP-**1**_0.4_**6**_0.6_	1.1	59%	0.59
AuNP-**1**_0.3_**6**_0.7_	3.0	n.d.	0.69
AuNP-**1**_0.2_**6**_0.8_^*e*^	5.0	n.d.	0.85
AuNP-**1**_0.1_**6**_0.9_^*e*^	8.0	88%^*e*^	0.88
AuNP-**6**	5.0	100%^*f*^	>0.97

^*a*^Molar equivalents with respect to initial concentration of nanoparticle-bound ligand **1**.

^*b*^Determined by *in situ*^19^F NMR (n.d. = not determined).

^*c*^Determined by exhaustive hydrazone exchange in the presence of excess 4-nitrobenzaldehyde (see ESI, Section 5).

^*d*^Experiments performed at higher initial concentrations of AuNP-**1** (see Table S2) tended to give lower than expected conversions, likely resulting from aggregation of aldehyde **3** and/or nanoparticle products.‖

^*e*^Nanoparticle precipitation observed.

^*f*^Dynamic covalent hydrazone exchange driven to completion by increasing the proportion of D_2_O to maintain nanoparticle solubility.

In a similar manner, a series of samples bearing monolayer compositions AuNP-**1**_*x*_**6**_*y*_ was prepared by treating AuNP-**1** with differing concentrations of aldehyde **3** (Table 2 and ESI, Section 4†).‖
‖Accurate determination of the stoichiometric excess of **3** was hampered by its hygroscopic nature and tendency to aggregate even at relatively low concentrations. When determining the monolayer compositions for this series, oxidative ligand stripping gave inconsistent results, owing to decomposition of the molecular components. An alternative method was therefore devised involving exhaustive hydrazone exchange in the presence of a large excess of 4-nitrobenzaldehyde to remove all **4** and **6** from the monolayer, allowing quantitative assessment of monolayer composition by ^1^H NMR (Table 2, see ESI, Section 5† for full details and further discussion).

**Fig. 3:**
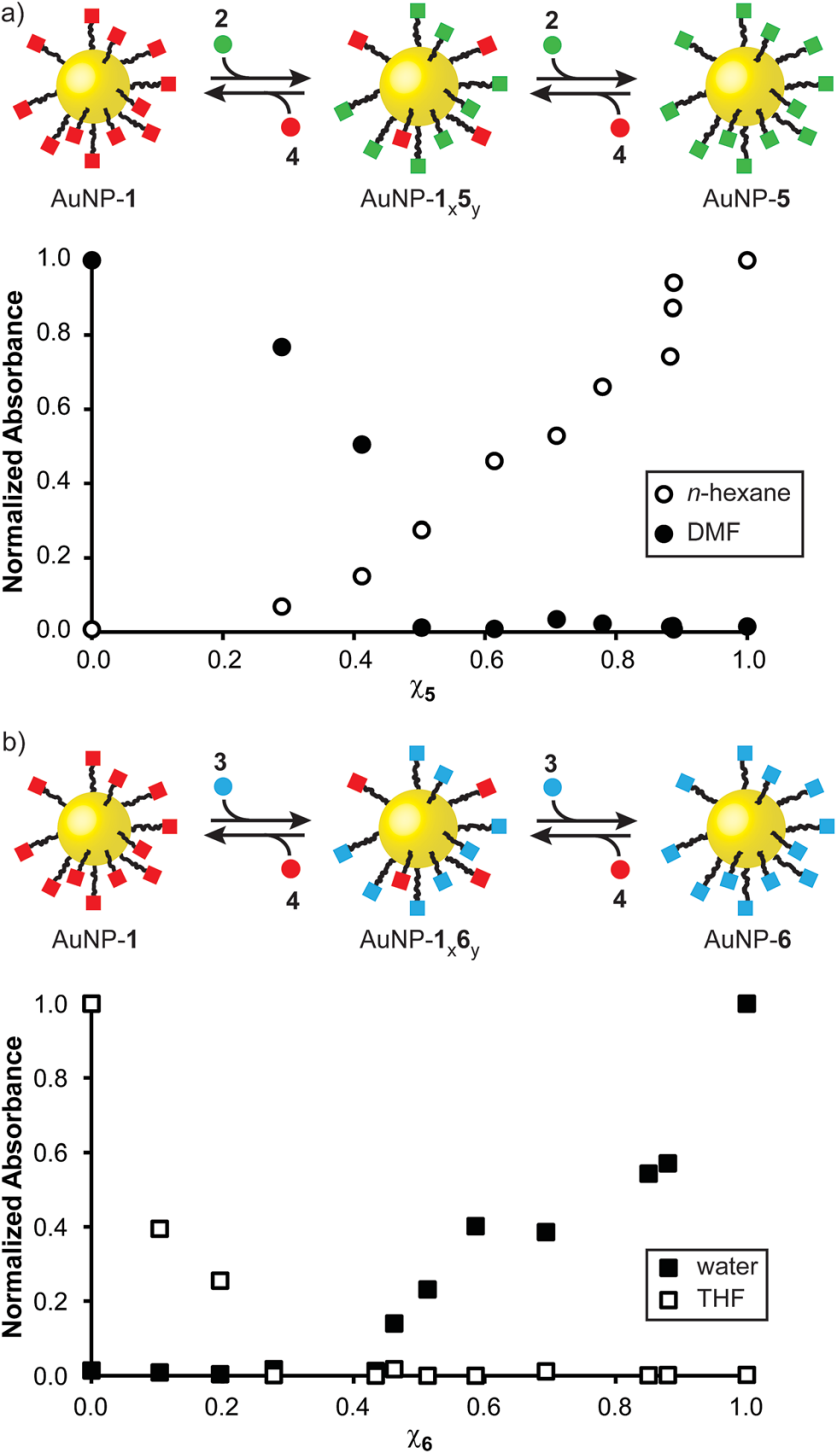
Continuum solubility tuning by dynamic covalent modification of monolayer composition. Absorbance values for saturated solutions of nanoparticle samples bearing a range of monolayer compositions from highly hydrophobic to highly hydrophilic. In both series, the second monolayer component is 4-fluorobenzylidine hydrazone **1**. Absorbance at 517 nm has been normalized to the value for a saturated solution of the most soluble sample for that solvent (*i.e.*, *n*-hexane: AuNP-**5**, 35 mg mL^–1^; DMF: AuNP-**1**, 41 mg mL^–1^; THF: AuNP-**1**, 34 mg mL^–1^; water: AuNP-**6**, 22 mg mL^–1^).

With monolayer compositions unambiguously established, we sought to correlate these with solvent compatibility. Solubility was semi-quantitatively assessed by preparing saturated nanoparticle solutions, then measuring the absorbance of the supernatant (or a known dilution thereof, see ESI Section 6† for details). Across the series AuNP-**1**_*x*_**5**_*y*_ significant variation in solubility was observed in both apolar and polar aprotic solvents. Solubility in *n*-hexane shows a monotonic increase with increasing mole fraction of hydrophobic **5** (*χ***_5_**) in the monolayer, from essentially insoluble for AuNP-**1** to a saturated solution concentration of 35 mg mL^–1^ for AuNP-**5** (Fig. 3a, open circles). For the same series in DMF, saturated concentration decreases quite sharply from the value of 41 mg mL^–1^ for AuNP-**1** as the proportion of hydrophobic **5** increases, with samples bearing >50% of the hydrophobic component essentially insoluble (Fig. 3a, filled circles).

Likewise, for the increasingly hydrophilic series AuNP-**1** → AuNP-**6**, solubility in a solvent of intermediate polarity (THF) drops off rapidly with increasing proportion of **6** (*χ***_6_**); samples above *χ***_6_** ≈ 0.3 becoming essentially insoluble (Fig. 3b, open squares). Although the high water solubility of AuNP-**6** (22 mg mL^–1^) is markedly decreased on introducing even 10% of hydrophobic ligand **1**, little influence of monolayer composition on solubility is observed in the range *χ***_6_** ≈ 0.9–0.6, where a good level of water solubility is maintained (>10 mg mL^–1^) despite the relatively significant proportions of **1** in the monolayer (Fig. 3b, closed squares). There is a sharp decrease in water solubility over the range *χ***_6_** ≈ 0.5–0.4, beyond which negligible solubility in water is observed.

Using only two monolayer components, this strategy therefore allows easy tuning of nanoparticle solubility characteristics between any two single-component monolayer extremes. Solubility is a complex phenomenon involving several factors. The trends observed here are in-line with those observed for wettability of mixed monolayers on extended surfaces,9c,^17^ consistent with interfacial energy being the key parameter that correlates monolayer composition with solvent compatibility. For both series investigated, it appears that there is a critical monolayer composition at which a qualitative transition from soluble to insoluble occurs, with the properties of one monolayer component dominating on either side of this point. This marked non-linearity in the relationship between monolayer composition and solubility is consistent with other binary mixed-ligand nanoparticle systems,8b,h and can be logically explained by considering the difference in length between ligand **1** and either **5** or **6**: it is likely that beyond a critical monolayer composition the properties of the shorter fluorinated ligand are effectively masked by the longer hydrophobic or hydrophilic ligands, with the result that the solvophilicity characteristics of the overall construct change rapidly around this point.

The more subtle changes in solubility observed as monolayer composition is varied appear to be ligand- and solvent-specific, emphasising the value of having access to a well-defined and self-consistent series of samples. It is notable that we do not observe any non-monotonic relationships between monolayer composition and solubility, in contrast to some other binary mixed-ligand nanoparticle systems, where such trends were ascribed to monolayer phase separation or ligand clustering.8b,9c,j The dynamic covalent strategy now enables the investigation of a wide range of ligand molecular structures and underlying nanoparticle scaffolds, where such effects as hierarchical monolayer patterning may be studied in detail. This can be achieved by relatively trivial modifications to the small-molecule aldehyde exchange units, thus avoiding the necessity for multi-step synthesis of numerous surface-active ligands in their entirety, and circumventing the unpredictability of ligand exchange or direct synthesis routes to nanoparticles bearing differing monolayer structures. Although precise monolayer composition control is facilitated by the intrinsically reversible nature of the transformations, any given monolayer composition can be isolated as a kinetically stable, covalently bound entity, which is therefore amenable to detailed structural and physicochemical characterization. Furthermore, the huge structural scope potentially accessible based on the maturity of abiotic small-molecule synthetic chemistry suggests that any number of properties determined by the NP-bound ligand shell may be studied in precisely the same manner.

## Monolayer composition dependent nanoparticle clustering

**Fig. 4:**
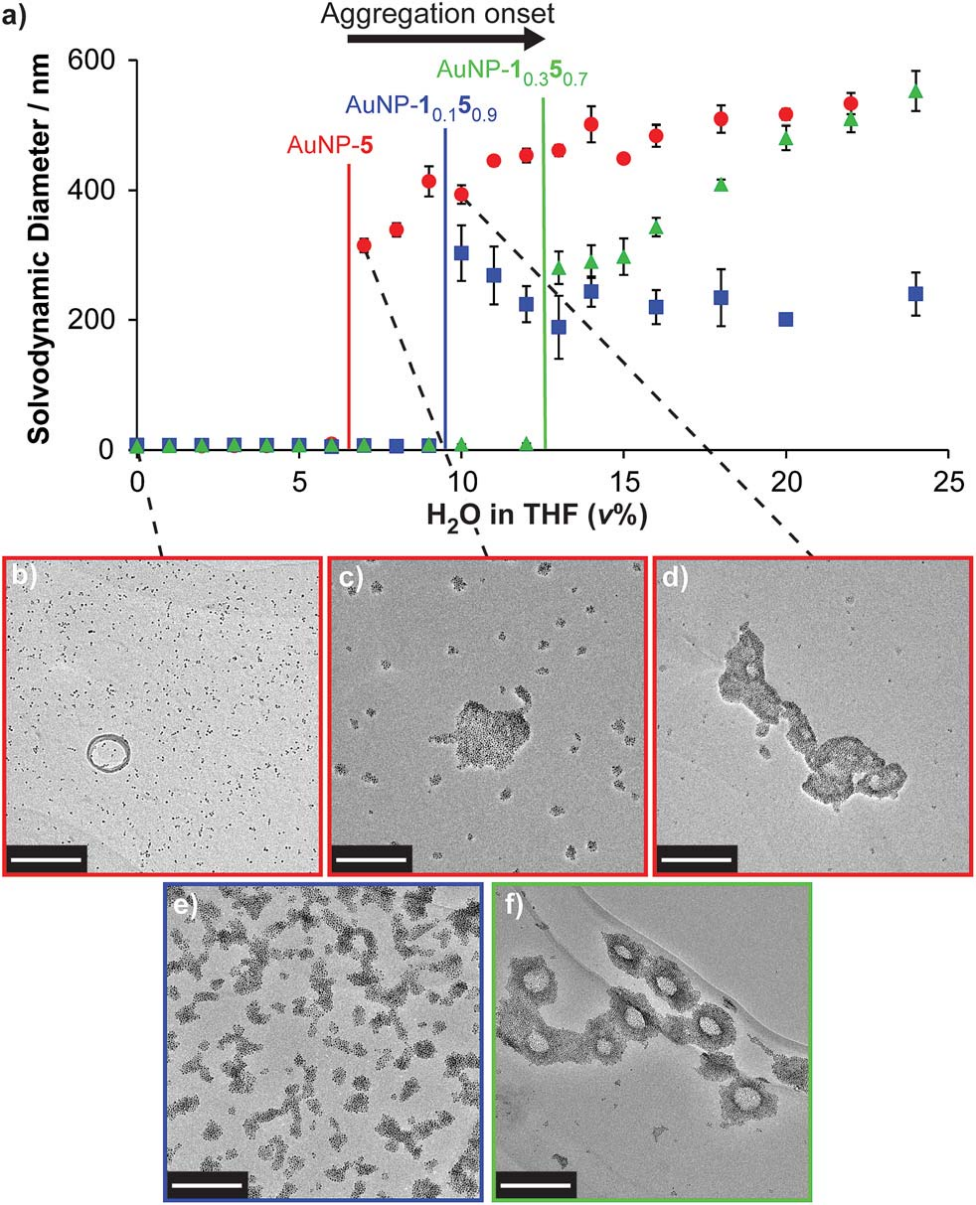
Monolayer composition-dependent solvophobic nanoparticle clustering. (a) Variation in solvodynamic size with solvent polarity as measured by DLS for AuNP-**5** (red circles), AuNP-**1**_0.1_**5**_0.9_ (blue squares), AuNP-**1**_0.3_**5**_0.7_ (green triangles). Monomodal size distributions were observed throughout; sizes are the mean of three measurements for distributions expressed in terms of % number of particles; error bars indicate ±1 standard deviation. See ESI, Section 7.1† for DLS measurements at higher proportions of water, and size distributions for selected sample points, expressed as both % number of particles and % volume. (b–f) Micrographs of dried samples corresponding to: (b) AuNP-**5**, 100% THF; (c) AuNP-**5**, 7% H_2_O/THF; (d) AuNP-**5**, 10% H_2_O/THF; (e) AuNP-**1**_0.1_**5**_0.9_, 10% H_2_O/THF; (f) AuNP-**1**_0.3_**5**_0.7_, 13% THF. Scale bar: 200 nm. See ESI, Section 7.2† for additional TEM images.

Finally, we examined the behaviour of mixed-ligand nanoparticles to incremental changes in solvent polarity. Good solubility in THF was exhibited across the series of particles AuNP-**1**_*x*_**5**_*y*_, so we investigated selected samples from this family in THF/water mixtures. Increasing volume fractions of water were introduced to THF solutions of each sample, then solvodynamic size was measured by dynamic light scattering (DLS). In neat THF, AuNP-**5** – the most oleophilic nanoparticle – exhibits a solvodynamic diameter ≈ 6.1 nm, consistent with well-dispersed particles. On increasing the proportion of water, a sharp increase in solvodynamic size is observed at only 7% H_2_O/THF (v%) (Fig. 4a, red circles). Remarkably, on raising solvent polarity further, rather than precipitation, discrete aggregates were observed, with solvodynamic diameters reaching a plateau in the range 450–550 nm for solvent compositions 12–30% H_2_O/THF (Fig. 4a and S14†). Some precipitation was observed only after the volume fraction of water exceeded *ca.* 25%. This behaviour was corroborated by TEM imaging. Micrographs of AuNP-**5** dropcast from neat THF (Fig. 4b and ESI, Section 7.2†) display individual nanoparticles or very small clusters that are difficult to differentiate from drying effects on the TEM grid. On the other hand, the sample prepared from 7% H_2_O/THF clearly shows discrete aggregates that are quite unlike any nonspecific effects observed for samples prepared from less polar solvents (Fig. 4c and ESI, Section 7.2†). Multiple clusters of relatively small size (*ca.* 25 nm) are visible, along with a smaller number of significantly larger aggregates several hundred nanometres across. The coexistence of large and small aggregates observed by TEM at this stage contrasts with the DLS measurements, which reported monomodal distributions for nearly all samples (ESI, Section 7.1†). This is likely indicative of a diverse population of aggregates that are in rapid exchange, and very sensitive to changes in concentration as samples are dried. At yet higher proportions of water, an increase in the number of large aggregates, at the expense of the smaller clusters, is observed by TEM (Fig. 4d and ESI, Section 7.2†), in-line with the DLS measurements.

Mixed-ligand monolayer samples exhibited qualitatively similar behaviour, but crucially, even small differences in monolayer composition changed the point at which the onset of aggregation is observed. Samples with higher proportions of the more polar ligand required increasingly polar solvents to induce aggregation: 10% H_2_O/THF and 13% H_2_O/THF for AuNP-**1**_0.1_**5**_0.9_ and AuNP-**1**_0.3_**5**_0.7_, respectively (Fig. 4a, blue squares and green triangles; see Fig. S14† for an expansion of the region 0–12% H_2_O/THF and for measurements at >25% H_2_O/THF). Again, TEM imaging was consistent with these observations (Fig. 4e, f and ESI, Section 7.2†). It is noteworthy that the behaviour of AuNP-**1**_0.1_**5**_0.9_ nicely explains our synthetic observation that achieving hydrazone exchange beyond 90% ligand **5** could not be achieved in 10% D_2_O/THF (Table 1), as a result of nanoparticle aggregation.

Solvent-dependent formation of discrete aggregates has been observed for nanoparticles stabilized by amphiphilic mixed-ligand monolayers in water – ascribed to shielding of a hydrophobic monolayer component in the aggregate interior, balanced by repulsive interactions between hydrophilic ligands.9e,g The work of Grzybowski, Bishop and Klajn has demonstrated reversible nanoparticle aggregation in apolar solvents on photoisomerization of azobenzene ligands,^18^ as a result of dipolar attraction and solvophobic interactions experienced by the polar (*Z*)-azobenzene isomer; however aggregation is disrupted in the presence of even small fractions of polar solvents such as methanol.^19^ By contrast, the AuNP-**1**_*x*_**5**_*y*_ series of nanoparticles exhibit solvophobic aggregation on increasing the solvent polarity of a majority organic matrix. Even from this un-optimized study, it is clear that the solvent composition at which the onset of aggregation occurs is sensitive to relatively small changes in monolayer composition. It is also intriguing to note that the maximum size of colloidally stable aggregates that are maintained in solution without precipitation is quite different for each sample (AuNP-**1**: *ca.* 580 nm; AuNP-**1**_0.1_**5**_0.9_: *ca.* 300 nm; AuNP-**1**_0.3_**5**_0.7_: *ca.* 740 nm, Fig. S14†). The absence of a monotonic trend to these values suggests a complex relationship between rather subtle differences in the monolayer composition and colloidal stability of the resulting aggregates, which demands further investigation. Notwithstanding these complexities, it should be expected that relatively simple variations to the ligand molecular structure or solvent characteristics could optimize for response to a wide variety of solvent systems, while the combination of solvent and ligand structure will determine the size of aggregates that can be stabilized in colloidal suspension. Such discrete, colloidally stable nanoparticle aggregates are of interest for controlling stoichiometry, valency and directionality of nanoparticle clusters;^20^ for bottom-up routes to colloidal superparticles;^21^ for preparing colloidally stable surface-enhanced spectroscopy platforms;^22^ and for constructing nanoparticle-lined artificial vesicles or nanoscale cavities.^19^,^23^

## Conclusions

The surface-stabilizing monolayer – defined by the stoichiometry and molecular structures of the constituent ligands – is a fundamental factor determining the characteristics and behaviour of any monolayer-stabilized nanoparticle.^2^ As well as myriad physicochemical properties, the ligand shell is critical to interactions with biomolecules and effects on living systems,^24^ recognition of small-molecule guests,^25^ surface accessibility for catalytic substrates,^26^ nanoparticle self-assembly,^27^ and integration with substrates or non-liquid matrices.^28^ Yet, methods for precisely engineering the monolayer – and in particular for reversibly tuning between multiple compositions – remain limited. Equally important is the challenge of accurately characterizing surface-bound monolayer composition so that it can be correlated with system properties and behaviour.^29^

We have shown that dynamic covalent exchange can be used to reversibly modify the molecular structure of nanoparticle-bound ligands; a variety of small-molecule exchange units can be introduced, generating either single-component monolayers of quite different molecular structures, or mixed-ligand systems with precise control over composition. The operationally simple exchange process is reversible, but each state can be isolated as a kinetically stable, all-covalent entity. This strategy has allowed us to switch between nanoparticles of widely differing solvation properties, spanning hexane to water; to gradually tune solvent compatibility between any two extremes; and to control the solvophobic formation of discrete self-assemblies – all from a single nanoparticle starting point.

Understanding the influence of monolayer composition on properties defined by solid–liquid interfaces is a long-standing challenge;9c,^17^,^30^ rediscovered in relation to colloidal nanomaterials. Having established robust characterisation of the monolayer using both *in situ* and *ex situ* methods, we can correlate each physicochemical state to a well-defined monolayer composition within a self-consistent series. This makes both for practically useful capabilities, and a powerful platform for systematic studies that will generate fundamental understanding about how monolayer composition translates into bulk properties.

Universal and predictable bottom-up synthetic strategies are required in order to break out from system-specific protocols and develop a genuinely general synthetic science for manipulating nano-sized chemical entities.^31^ Dynamic covalent exchange within the nanoparticle-bound monolayer is independent of both the preparation of the underlying nanomaterial, and the specific nature of the nanoparticle–ligand bond, suggesting that this method for modifying surface-bound molecules *in situ* should be generalizable. Using this divergent synthetic strategy, a relatively small set of dynamic covalent nanoparticle building blocks with optimized core properties may therefore be transformed easily and rapidly into a wide range of functionalized and responsive nanoparticle systems, devices and materials, thus contributing to a new generation of synthetic capabilities for manipulating nanoscale synthons with the same level of control as can currently be achieved for molecular building blocks.^3^

## Conflicts of interest

There are no conflicts to declare.

## Supplementary Material

Supplementary information
