# Supplementary material for: Continuum tuning of nanoparticle interfacial properties by dynamic covalent exchange
Source: Chem Sci. 2017 Nov 17;9(1):125–33. doi: 10.1039/c7sc03666c (PMC5869618; doi:10.1039/c7sc03666c)
Supplement: Supplementary file 1 [file SC-009-C7SC03666C-s001.pdf]

Electronic Supplementary Material (ESI) for Chemical Science.
This journal is © The Royal Society of Chemistry 2017

Supporting Information

Continuum tuning of nanoparticle interfacial properties by dynamic covalent exchange

William Edwards, Nicolas Marro, Grace Turner and Euan R. Kay*

1. General experimental procedures S2
2. Synthesis of molecular species S3
3. Synthesis and characterization of AuNP-**1** S6
   3.1 Hexanethiyl-stabilized nanoparticles (AuNP-S**3**) S6
   3.2 4-Fluorobenzylidine hydrazone functionalized nanoparticles (AuNP-**1**) S7
4. Nanoparticle-bound dynamic covalent hydrazone exchange S9
   4.1 Single-component monolayer AuNP-**5** S10
   4.2 Single-component monolayer AuNP-**6** S14
   4.3 Continuum tuning of monolayer composition S18
5. Determination of mixed monolayer composition S19
6. Assessment of nanoparticle solubility properties S24
7. Solvophobic nanoparticle self-assembly S26
   7.1 Assessment of solvodynamic size distributions by dynamic light scattering S26
   7.2 Supplementary TEM images characterizing solvophobic nanoparticle self-assembly S30
8. ^{1}H and ^{13}C NMR spectra of organic compounds S33
9. Size distributions and representative TEM images for nanoparticle samples S38
   Comment on size distributions S43
10. Supplementary references S44

1. General experimental procedures

Unless stated otherwise, all reagents were purchased from commercial sources and used without further purification. Dry solvents were obtained by means of a MBRAUN MB SPS-800™ solvent purification system, where solvents were passed through filter columns and dispensed under an argon atmosphere. Flash column chromatography was performed using Geduran® Si60 (40–63 μm, Merck, Germany) as the stationary phase. Thin-layer chromatography (TLC) was performed on pre-coated silica gel plates (0.25 mm thick, $60F_{254}$, Merck, Germany) and observed under UV light (λ_{max} 254 nm), or visualized by staining with a basic potassium permanganate solution, followed by heating. Nanoparticle micrographs were obtained using a JEM 2010 transmission electron microscope (TEM) on samples prepared by deposition of one drop of nanoparticle suspension on holey carbon films supported on a 300 mesh Cu grid (Agar Scientific®). Nanoparticle diameters were measured automatically using the software *ImageJ*. The images were first converted to black and white images using the "Threshold" function; the area of each nanoparticle was measured using the "Analyze particles" function; particles on edges were excluded. UV-vis absorption spectroscopy was performed on a Thermo Scientific Evolution 220 UV-Visible Spectrophotometer using a quartz cuvette (0.5 mm path length). Dynamic light scattering (DLS) measurements were performed on a Malvern Zetasizer μV instrument using a glass cuvette (10 mm path length). ^{1}H, ^{13}C, and ^{19}F NMR spectra were recorded on Bruker Avance II 300, 400 and 500 instruments, at a constant temperature of 25 °C. ^{1}H Chemical shifts are reported in parts per million (ppm) from low to high field and referenced to the literature values for chemical shifts of residual non-deuterated solvent, with respect to tetramethylsilane. ^{19}F Chemical shifts are referenced to CF_3Cl (0.00 ppm) as external standard. Standard abbreviations indicating multiplicity are used as follows: bs (broad singlet), d (doublet), dd (doublet of doublets), m (multiplet), q (quartet), s (singlet), t (triplet), tt (triplet of triplets), *J* (coupling constant). All spectra were analyzed using MestReNova (Version 9.0.0). All melting points were determined using a Stuart SMP30 Melting Point Apparatus and are reported uncorrected. Freeze drying was achieved using a Christ Alpha 1–2 LD Freeze dryer (Martin Christ GmbH, Osterode am Harz, Germany) at −54 °C, 0.27 mbar vacuum for ca. 15–20 h until complete dryness.

2. Synthesis of molecular species

Chloro(triphenylphosphine)gold(I) and 3-(undecyloxy)benzaldehyde (**2**) were prepared as previously described.[1]

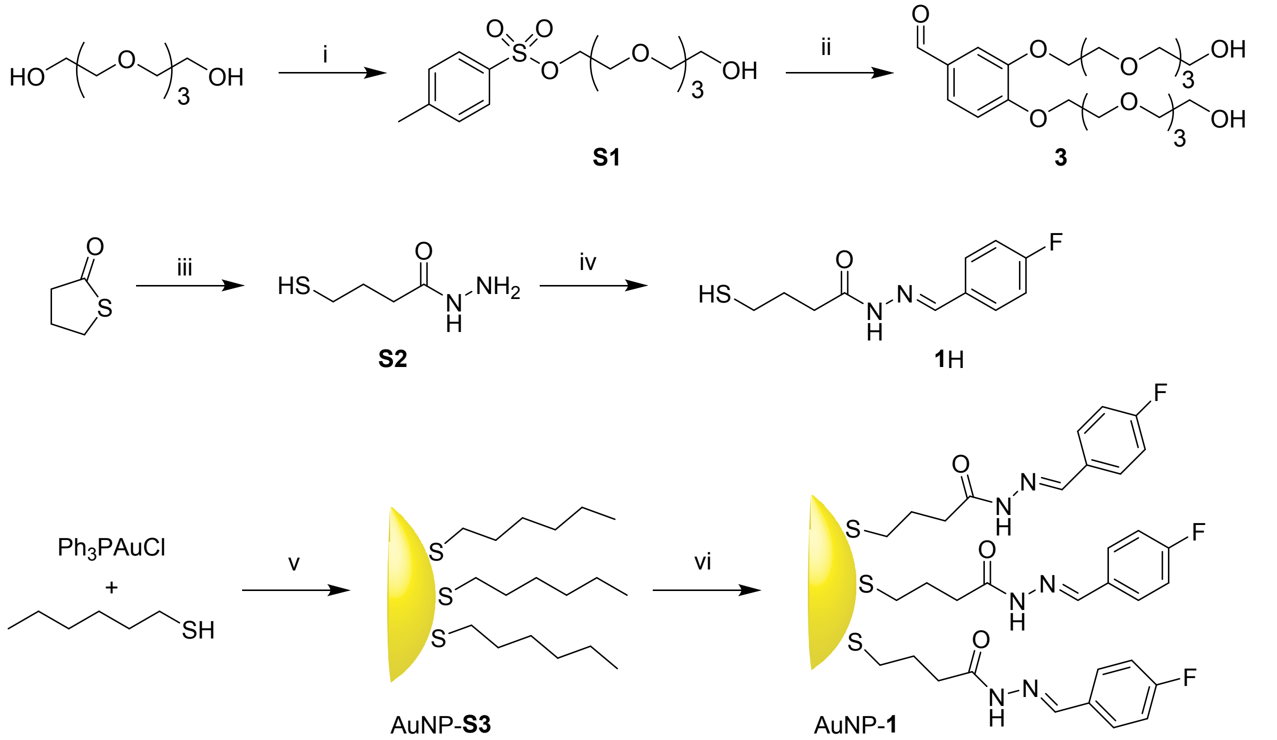

Scheme S1. Summary of synthetic procedures. Reagents and conditions: (i) p-toluenesulfonyl chloride, NaOH, tetrahydrofuran (THF), 0 °C to r.t., 16 h, 76%; (ii) 3,4-dihydroxybenzaldehyde, K_2CO_3, *N*,*N*-dimethylformamide (DMF), 100 °C, 2.5 d, 43%; (iii) $H_2NNH_2{\bullet}H_2O$, MeOH, r.t., 1.5 h, 96%; (iv) 4-fluorobenzaldehyde, AcOH/EtOH (5 % *v*/*v*), r.t., 2 h, 20 %; (v) *t*-$BuNH_2{\bullet}BH_3$, $CHCl_3$, 55 °C to r.t., 4 h; (vi) **1**H, THF, 5 d.

Tetraethylene glycol mono(*p*-toluenesulfonate) (S1)

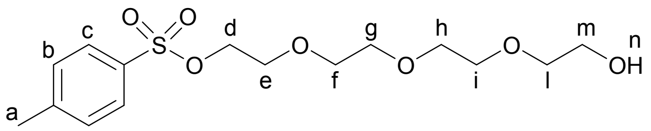

Tetraethylene glycol (60 g, 0.309 mol) and dichloromethane (90 mL) were combined and cooled to 0 °C. Et_3N (5.01 g, 0.049 mol) was added, followed by dropwise addition of *p*-toluenesulfonyl chloride (5.89 g, 0.039 mol) in dichloromethane (10 mL) over 30 min. The mixture was allowed to warm to room temperature and stir for 18 h. Water (200 mL) was then added and the organic layer removed. The aqueous phase was extracted with CH_2Cl_2 (2 × 200 mL). The combined organic phases were washed with H_2O (2 × 200 mL) and brine (sat., 2 × 200 mL) before being dried over $MgSO_4$, filtered and volatiles removed under vacuum to yield **S1** as a colourless oil. Yield = 9.92 g (92%). ^{1}H NMR (500 MHz, $CDCl_3$) δ 7.79 (2H, d, *J* = 8.3 Hz, c), 7.34 (2H, d, *J* = 8.1 Hz, b), 4.17 – 4.14 (2H, m, d), 3.72 – 3.58 (14H, e, f, g, h, i, l, m), 2.44 (3H, s, a), 2.24 (1H, bs, n). $^{13}C\{^1H\}$ NMR (126 MHz, $CDCl_3$) δ 144.9, 133.0, 129.9, 128.1, 72.6, 70.9, 70.8, 70.6, 70.4, 69.4, 68.9, 61.9, 21.8. HRMS (ESI) calculated *m*/*z* for $C_{15}H_{25}O_7S$ $[M+H]^+$ = 349.1316, found 349.1320 (100%).

3,4-Bis(2-(2-(2-(2-hydroxyethoxy)ethoxy)ethoxy)ethoxy)benzaldehyde (3)

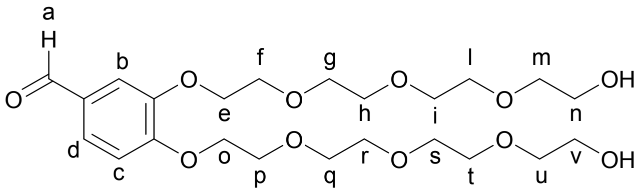

3,4-Dihydroxybenzaldehyde (1.05 g, 7.59 mmol) and K_2CO_3 (2.31 g, 16.7 mmol) dissolved in *N*,*N*-dimethylformamide (40 mL) under an Ar atmosphere and the mixture heated to 100 °C. Tetraethylene glycol mono(*p*-toluenesulfonate) (**S1**) (5.55 g, 15.9 mmol) in *N*,*N*-dimethylformamide (15 mL) was added dropwise over 1 h and then the mixture was stirred at 100 °C for 2.5 d. After cooling to rt, the volatiles were removed under vacuum and the residue partitioned between CH_2Cl_2 and K_2CO_3 (10% aq.). The organic phase was removed and the aqueous phase extracted a further three times with CH_2Cl_2. The combined organics were then washed twice with K_2CO_3 (10% aq.), dried over $MgSO_4$, filtered and volatiles removed under vacuum. This crude product was purified by column chromatography (SiO_2, CH_2Cl_2 to CH_2Cl_2/MeOH 9:1) providing **3** as a yellow oil. Yield =1.59 g (43%). ^1H NMR (500 MHz, $CDCl_3$) δ 9.83 (1H, s, a), 7.45 – 7.42 (2H, m, b, d), 6.99 (1H, d, J = 8.2 Hz, c), 4.24 (4H m, e, o), 3.90 (4H, m, p, f), 3.77 – 3.58 (24H, m, g, h, i, l, m, q, r, s, t, u, v). $^{13}C\{^1H\}$ NMR (126 MHz, $CDCl_3$) δ 190.9, 154.3, 149.1, 130.2, 126.7, 112.4, 111.8, 72.58, 72.56, 71.0, 70.9, 70.7, 70.6, 70.4, 70.3, 69.6, 69.4, 68.8, 68.7, 61.7 (×2). HRMS (ESI) calculated *m*/*z* for $C_{23}H_{38}O_{11}Na$ $[M+Na]^+$ = 513.2306, found 513.2300 (100%).

4-Mercaptobutanehydrazide (S2)

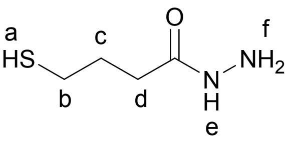

γ-Thiobutyrolactone (3.00 mL, 3.54 g, 34.7 mmol) and hydrazine monohydrate (3.36 mL, 3.47 g, 69.3 mmol) were mixed in MeOH (30 mL) and allowed to stir at rt for 1.5 h. Volatiles were removed under vacuum and the crude product purified by column chromatography (SiO_2, Et_2O/MeOH 5:1) to afford **S2** as a colourless oil. Yield = 4.47 g (96%). ^1H NMR (400 MHz, $CDCl_3$) δ 7.15 (1H, br s, e), 3.92 (2H, br s, f), 2.57 (2H, dt, *J* = 8.0, 7.2 Hz, b), 2.30 (2H, t, *J* = 7.2 Hz, d), 1.95 (2H, m, *J* = 7.2 Hz, c), 1.34 (1H, t, *J* = 8.0 Hz, a). $^{13}C\{^1H\}$ NMR (126 MHz, $CDCl_3$) δ 173.1, 32.6, 29.3, 24.2. HRMS (ESI) calculated *m*/*z* for $C_4H_{10}N_2OSNa$ $[M+Na]^+$ = 157.0406, found 157.0404 (100%).

***N′*-(4-Fluorobenzylidene)-4-mercaptobutanehydrazide (1H)**

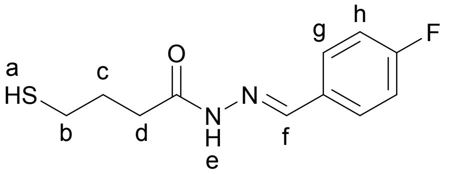

4-Mercaptobutanehydrazide **S2** (4.16 g, 31.0 mmol) and 4-fluorobenzaldehyde (3.99 mL, 4.62 g, 37.2 mmol) were mixed in ethanolic AcOH (5% *v*/*v*, 50 mL). The reaction was stirred at rt for 2 h. Volatiles were removed under vacuum and the crude residue purified by column chromatography (SiO_2, cyclohexane/EtOAc 2:1 to 1:1). Product containing fractions were combined and dissolved in CH_2Cl_2, to which was added Et_2O to yield a precipitate. Filtration and drying under vacuum provided **1**H as a colourless solid. Yield = 1.5 g (20%). m.p. 93–95 °C. ^1H NMR (500 MHz, CD_2Cl_2) δ 9.71 (1H, s, e (major rotamer)), 8.86 (1H, s, e (minor rotamer)), 8.08 (1H, s, f (minor)), 7.78 (1H,s, f (major)), 7.69 (2H, m, g), 7.12 (2H, t, *J* = 8.5 Hz, h), 2.87 (2H, t, *J* = 7.4 Hz, d (major)), 2.64 (2H, dt (app. q), *J* = 7.4, b), 2.42 (2H, t, *J* = 7.2 Hz, d (minor)), 2.01 (2H, m, c), 1.47 (1H, t, *J* = 8.0

Hz, a). ^{19}F NMR (470 MHz, CD_2Cl_2) δ –110.30 (minor rotamer), –110.81 (major rotamer). ^{13}C{^{1}H} NMR (126 MHz, CD_2Cl_2) δ 175.62, 163.9 (d, *J* = 251 Hz), 142.7, 129.9 (d, *J* = 3 Hz), 129.4 (d, *J* = 9.1 Hz), 116.3 (d, *J* = 22 Hz), 31.7, 29.5, 24.8. HRMS (ESI) calculated *m*/*z* for $C_{11}H_{14}FN_2OS$ $[M+H]^+$ = 241.0805, found 241.0806 (100%).

3. Synthesis and characterization of AuNP-1

3.1 Hexanethiyl-stabilized nanoparticles (AuNP-S3)

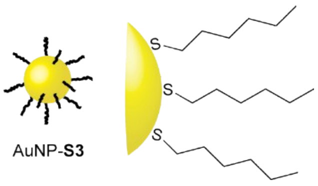

Using a modified version of the synthetic procedure originally developed by Stucky and co-workers,[2] Ph_3PAuCl (1.00 g, 2.02 mmol) was weighed into a 250 mL round bottom flask and dissolved in $CHCl_3$ (160 mL). Hexanethiol (0.603 mL, 0.502 g, 4.25 mmol) was added and the reaction mixture heated to 55 °C. *tert*-Butylamine borane complex (1.76 g, 20.2 mmol) was added in one portion as a powdered solid and the reaction was held at 55 °C for 1 h before being allowed to stir at room temperature for 3 h. The solution was transferred to a 1 L flask to which a large quantity of MeOH was added before standing in the freezer overnight. The supernatant was removed, the residue dissolved in the minimal volume of CH_2Cl_2 and transferred to a vial, then the solvent removed under a stream of compressed air. The residue was dispersed in MeOH with sonication, then subjected to centrifugation (1516×g rcf, 4 °C, 40 min). The supernatant was removed, and the pellet redispersed in fresh solvent. The nanoparticles were washed repeatedly in this manner with MeOH (×7), then MeCN (×3), after which no further impurities were observed in the supernatant by TLC and ^{1}H NMR analysis. The residue was then dried under vacuum to afford AuNP-**S3** as an amorphous black solid.

TEM size analysis (Figure S2) revealed nanoparticles with average diameter of 4.32 nm and standard deviation 0.96 nm (dispersity ca. 22%), consistent with literature reports for hexanethiyl-stabilized nanoparticles prepared under similar conditions.[3]

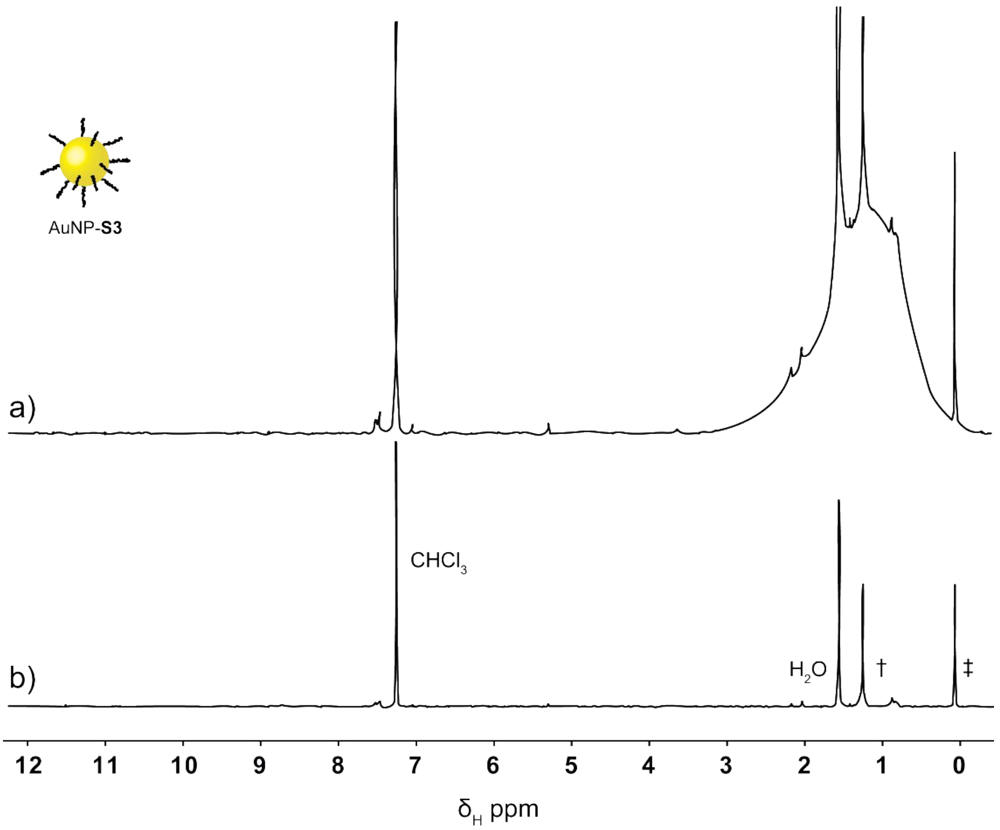

Figure S1. NMR Characterization of AuNP-**S3**. a) ^{1}H NMR spectrum (500 MHz, $CDCl_3$, 32 scans). b) T_2-Filtered ^{1}H NMR spectrum (500 MHz, $CDCl_3$, 8 scans) acquired using the CPMG-z pulse sequence.[4] All sharp signals can be assigned to residual non-deuterated solvents and impurities as indicated († = H grease: δ 0.84–0.87 (m), δ 1.25 (br s); ‡ = silicone grease: δ 0.07 (s)).

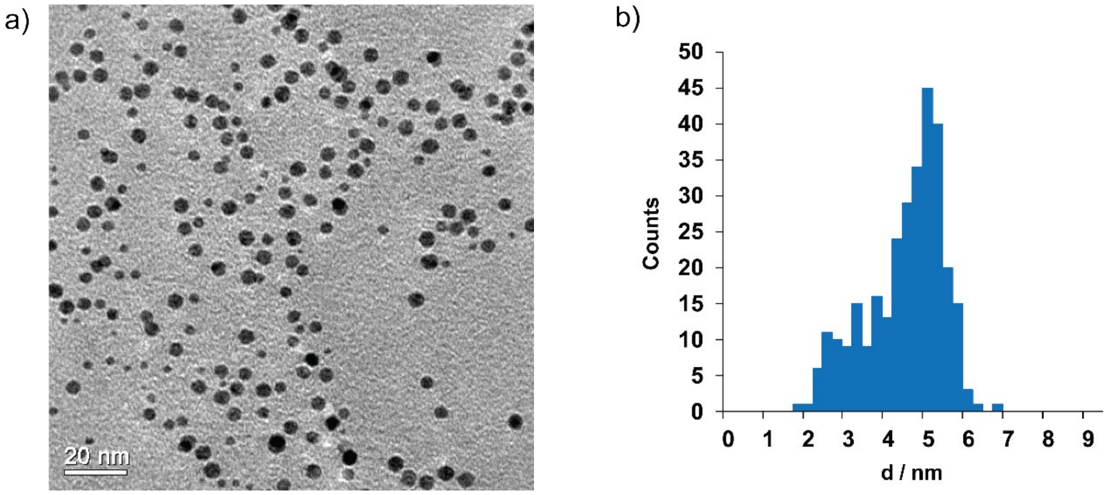

Figure S2. a) Representative TEM image and b) histogram of size distribution as found through analysis of multiple images for hexanethiyl-stabilized AuNP-**S3** (d = 4.32 ± 0.96 nm).

3.2 4-Fluorobenzylidine hydrazone functionalized nanoparticles (AuNP-1)

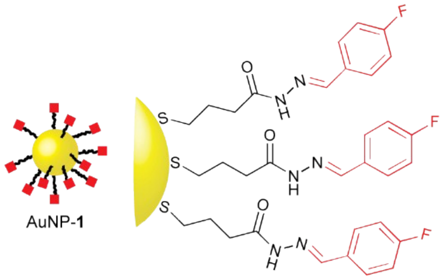

Hexanethiyl-stabilized nanoparticles AuNP-**S3** (276 mg) were suspended in THF (25 mL), the mixture sonicated to ensure good dispersion of the nanoparticles, then placed under an Ar atmosphere. *N′*-(4-Fluorobenzylidene)-4-mercaptobutanehydrazide (**1**H, 300 mg, 1,25 mmol) was added and the mixture was stirred in the dark for 5 d. Volatiles were removed under a stream of compressed air, the residue re-suspended in MeCN and subjected to centrifugation (1312×g rcf, 4 °C, 20 min). The supernatant was removed and discarded. In the same manner, the nanoparticles were subjected to multiple cycles of washing in MeCN (×5) and THF/cyclohexane (×5). After the final washing, the nanoparticles were dried under gentle air flow, then freeze-dried overnight to provide AuNP-**1** as an amorphous black solid.

TEM analysis revealed a mean particle diameter of 3.80 nm and standard deviation 1.02 nm (representative micrograph images and size distribution histograms for all hydrazone-functionalized nanoparticles can be found in Section 9). Although within one standard deviation of both samples, the reduction in mean nanoparticle size during ligand exchange is likely the result of thiol-induced etching or other rearrangement processes of the ligand shell that can occur on extended exposure of gold nanoparticles to an excess of alkylthiols in solution environments.[3, 5] It should be noted that the ligand exchange was allowed to procede for 5 days to ensure exhaustive exchange; this extended reaction time may well have contributed to allowing intinsic changes in nanoparticle size distribution to occur, which could likely be minimized by optimizing the protocol to minimize reaction time.

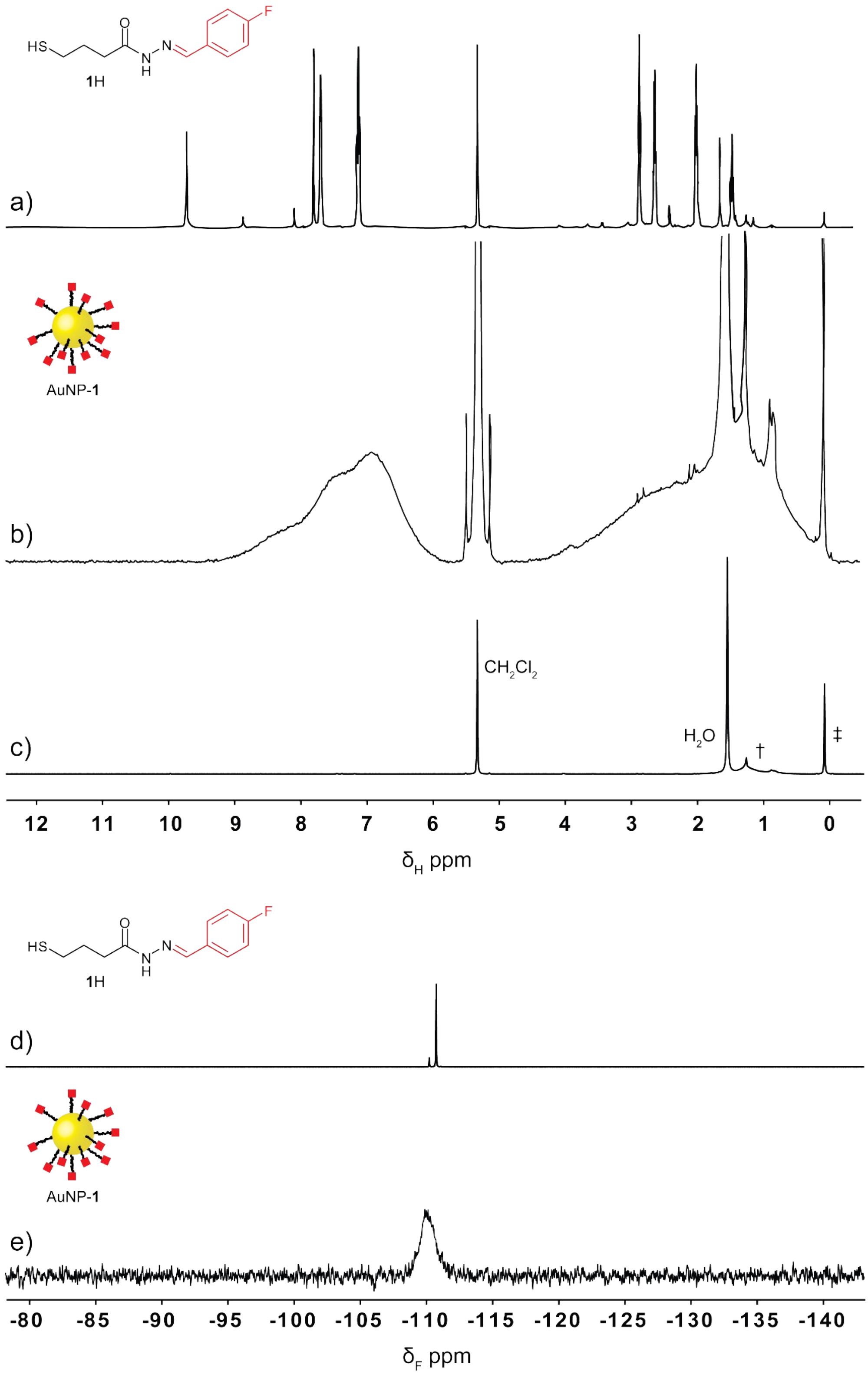

Figure S3. NMR Characterization of AuNP-**1**. a) ^{1}H NMR (500 MHz, CD_2Cl_2, 8 scans) spectrum of ligand **1**H. b) ^{1}H NMR (500 MHz, CD_2Cl_2, 32 scans) spectrum of AuNP-**1**. c) T_2-Filtered ^{1}H NMR (500 MHz, CD_2Cl_2, 8 scans) spectrum of AuNP-**1** acquired using the CPMG-z pulse sequence.[4] All sharp signals can be assigned to residual non-deuterated solvents and impurities as indicated († = H grease: δ 0.84–0.87 (m), δ 1.25 (br s); ‡ = silicone grease: δ 0.07 (s)). d) ^{19}F NMR (470 MHz, CD_2Cl_2, 128 scans) spectrum of ligand **1**H. e) ^{19}F NMR (470 MHz, CD_2Cl_2, 128 scans) spectrum of AuNP-**1**.

4. Nanoparticle-bound dynamic covalent hydrazone exchange

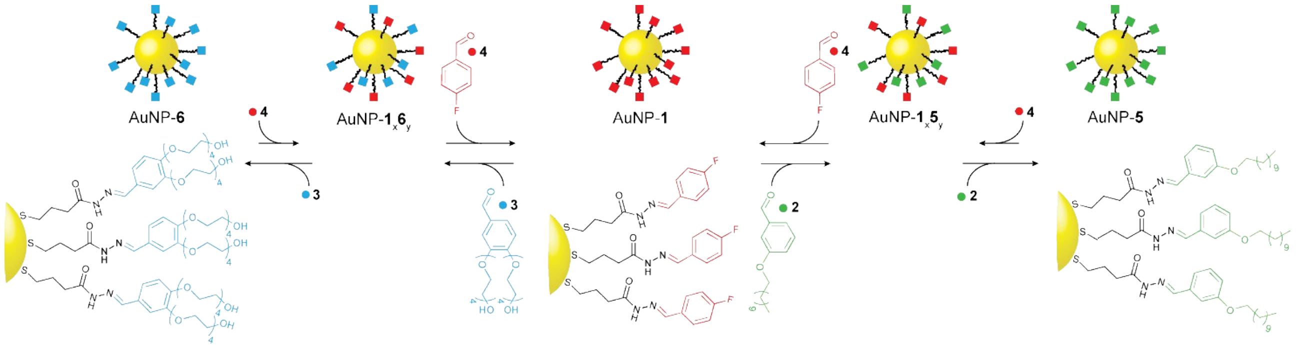

Table S1. Summary of AuNP samples functionalized with components **1** and **5** at various compositions.

Sample	[AuNP-**1**]$_0$ / mM [a]	equivalents **2** added [a]	% **4** released [b]	χ_1 [c]	χ_5 [c]	*d* / nm (± s.d.) [d]
AuNP-**1**	N/A	N/A	N/A	1.00	0.00	3.80 (1.02)
AuNP-$\mathbf{1}_{0.7}\mathbf{5}_{0.3}$	8.7	0.26	29%	0.71	0.29	3.98 (0.68)
AuNP-$\mathbf{1}_{0.6}\mathbf{5}_{0.4}$	6.5	0.52	37%	0.59	0.41	4.36 (0.97)
AuNP-$\mathbf{1}_{0.5}\mathbf{5}_{0.5}$	8.3	0.54	46%	0.50	0.50	3.71 (0.62)
AuNP-$\mathbf{1}_{0.4}\mathbf{5}_{0.6}$	8.5	0.80	56%	0.39	0.61	3.97 (0.56)
AuNP-$\mathbf{1}_{0.3}\mathbf{5}_{0.7}$	6.1	1.1	68%	0.29	0.71	4.67 (0.96)
AuNP-$\mathbf{1}_{0.2}\mathbf{5}_{0.8}$	6.3	2.1	79%	0.22	0.78	4.77 (0.82)
AuNP-$\mathbf{1}_{0.1}\mathbf{5}_{0.9}$-a [e]	6.6	3.1	89% [e]	0.12	0.88	4.58 (0.81)
AuNP-$\mathbf{1}_{0.1}\mathbf{5}_{0.9}$-b [e]	6.6	4.1	85% [e]	0.11	0.89	4.51 (0.77)
AuNP-$\mathbf{1}_{0.1}\mathbf{5}_{0.9}$-c [e]	6.5	5.2	89% [e]	0.11	0.89	4.29 (0.89)
AuNP-**5** [f]	5.2	5.1	100% [f]	< 0.03 [g]	> 0.97	4.74 (0.78)

[a] Initial concentration of AuNP-**1**, and molar equivalents of **2**, expressed in terms of concentration of ligand **1**.
[b] Determined by *in situ* ^{19}F NMR.
[c] Determined by oxidative ligand stripping using I_2 (see Section 5).
[d] Determined from a minimum of 200 measurements taken from several TEM images (see Section 9).
[e] Nanoparticle precipitation observed.
[f] Dynamic covalent hydrazone exchange driven to completion by increasing the proportion of CH_2Cl_2 to maintain nanoparticle solubility.
[g] Undetectable by ^{19}F NMR (470 MHz, THF/CH_2Cl_2/D_2O (9:2:0.5), 16 scans, recycle delay = 25 s).

Table S2. Summary of AuNP samples functionalized with components **1** and **6** at various compositions.

Sample	[AuNP-**1**]$_0$ / mM [a]	equivalents **3** added [a]	% **4** released [b]	χ_1 [c]	χ_6 [c]	d / nm (± s.d.) [d]
AuNP-**1**	N/A	N/A	N/A	1.00	0.00	3.80 (1.02)
AuNP-$\mathbf{1}_{0.9}\mathbf{6}_{0.1}$	10.0	0.1	n.d.	0.90	0.10	3.83 (0.77)
AuNP-$\mathbf{1}_{0.8}\mathbf{6}_{0.2}$ [e]	9.6	0.5	n.d.	0.80	0.20	3.93 (0.79)
AuNP-$\mathbf{1}_{0.7}\mathbf{6}_{0.3}$	7.8	0.3	26%	0.72	0.28	3.84 (0.75)
AuNP-$\mathbf{1}_{0.6}\mathbf{6}_{0.4}$	7.7	0.5	45%	0.57	0.43	3.83 (0.71)
AuNP-$\mathbf{1}_{0.5}\mathbf{6}_{0.5}$-a [e]	9.9	1.0	n.d.	0.54	0.46	3.95 (0.80)
AuNP-$\mathbf{1}_{0.5}\mathbf{6}_{0.5}$-b	8.0	0.7	52%	0.49	0.51	3.87 (0.71)
AuNP-$\mathbf{1}_{0.4}\mathbf{6}_{0.6}$	7.7	1.1	59%	0.41	0.59	3.65 (0.67)
AuNP-$\mathbf{1}_{0.3}\mathbf{6}_{0.7}$	9.2	3.0	n.d.	0.31	0.69	3.69 (0.80)
AuNP-$\mathbf{1}_{0.2}\mathbf{6}_{0.8}$ [f]	9.6	5.0	n.d. [f]	0.15	0.85	3.73 (0.74)
AuNP-$\mathbf{1}_{0.1}\mathbf{6}_{0.9}$ [f]	6.3	8.0	88% [f]	0.12	0.88	3.61 (0.95)
AuNP-**6** [g]	4.9	5.0	100% [g]	< 0.03 [h]	> 0.97	3.90 (0.94)

[a] Initial concentration of AuNP-**1**, and molar equivalents of **3**, expressed in terms of concentration of ligand **1**.
[b] Determined by *in situ* ^{19}F NMR (n.d. = not determined).
[c] Determined by exhaustive hydrazone exchange in the presence of excess 4-nitrobenzaldehyde (see Section 5).
[d] Determined from a minimum of 200 measurements taken from several TEM images (see Section 9).
[e] Experiments performed at higher initial concentrations of AuNP-**1** tended to give lower than expected conversions (compare AuNP-$\mathbf{1}_{0.8}\mathbf{6}_{0.2}$ with AuNP-$\mathbf{1}_{0.6}\mathbf{6}_{0.4}$; and AuNP-$\mathbf{1}_{0.5}\mathbf{6}_{0.5}$-a with AuNP-$\mathbf{1}_{0.4}\mathbf{6}_{0.6}$), likely resulting from aggregation of aldehyde **3** and/or nanoparticle products.
[f] Nanoparticle precipitation observed.
[g] Dynamic covalent hydrazone exchange driven to completion by increasing the proportion of D_2O to maintain nanoparticle solubility.
[h] Undetectable by ^{19}F NMR (470 MHz, THF/D_2O (9:1), 16 scans, recycle delay = 25 s).

4.1 Single-component monolayer AuNP-5

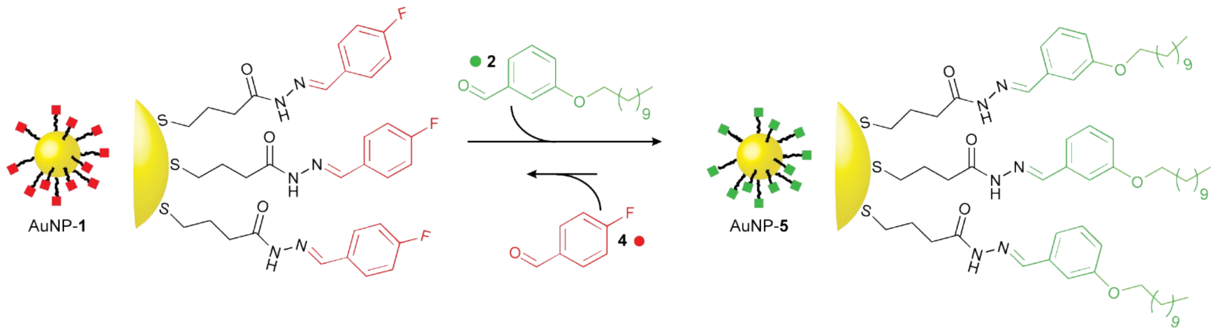

Concentrations of all fluorine-containing species were determined by quantitative ^{19}F NMR in the presence of 4-fluorotoluene as an internal standard of known concentration.

The following stock solutions were prepared:

Aldehyde **2** (0.322 M) with 4-fluorotoluene (5.00 mM) in THF/CH_2Cl_2/D_2O (9:2:0.5).

CF_3CO_2H (0.989 M) with 4-fluorotoluene (5.00 mM) in THF/CH_2Cl_2/D_2O (9:2:0.5).

A solution containing AuNP-**1** (7.5 mg) and 4-fluorotoluene (5.00 mM) was prepared in THF/CH_2Cl_2/D_2O (9:2:0.5, 550 μL), giving 5.78 mM in terms of 4-fluorobenzylidine hydrazone (AuNP-**1**, 3.18 μmol). To this was added an aliquot of aldehyde **2** stock solution (50.0 μL, 16.1 μmol), followed by CF_3CO_2H stock solution (12.1 μL 12.0 μmol), giving final concentrations of

AuNP-**1** (5.16 mM), **2** (26.3 mM, 5.1 equiv.) and CF_3CO_2H (19.6 mM). The mixture was heated to 50 °C. Monitoring by ^{19}F NMR revealed decreasing intensity for the broad signal corresponding to nanoparticle-bound **1** and quantitative conversion to a sharp signal for aldehyde **4** (Figure S4). After 19.5 h, the signal for nanoparticle-bound **1** was no-longer visible and the concentration of released aldehyde **4** reached 3.24 mM (63% conversion). However, some precipitate was observed at this stage and no further change in composition occurred over the next 6.5 h. Addition of CH_2Cl_2 (50 μL) brought all components back into solution and the mixture was left at 50 °C for a further 24.5 h. After this time, ^{19}F NMR analysis indicated 100% conversion of the nanoparticle-bound hydrazone according to the concentration of released aldehyde **4**. A further 5 equivalents aldehyde **2** were added to ensure complete conversion: no change was observed over 17 h at 50 °C.

The mixture was cooled to room temperature and solvent volume reduced by half under a stream of compressed air. Nanoparticle precipitation was induced by addition of MeCN/EtOH (5:1, 10 mL), followed by sonication (10 min, 20 °C), and centrifugation (1935×g rcf, 10 min, –4 °C). The colourless supernatant was carefully discharged and washing repeated a further twice with the same solvent mixture, followed by three more washes using MeCN/EtOH/H_2O (7.5:2.5:1, 10 mL). Traces of volatile solvents were removed under a stream of compressed air, 1 mL water added, and the sample freeze dried to provide quantitatively exchanged AuNP-**5** (6.3 mg).

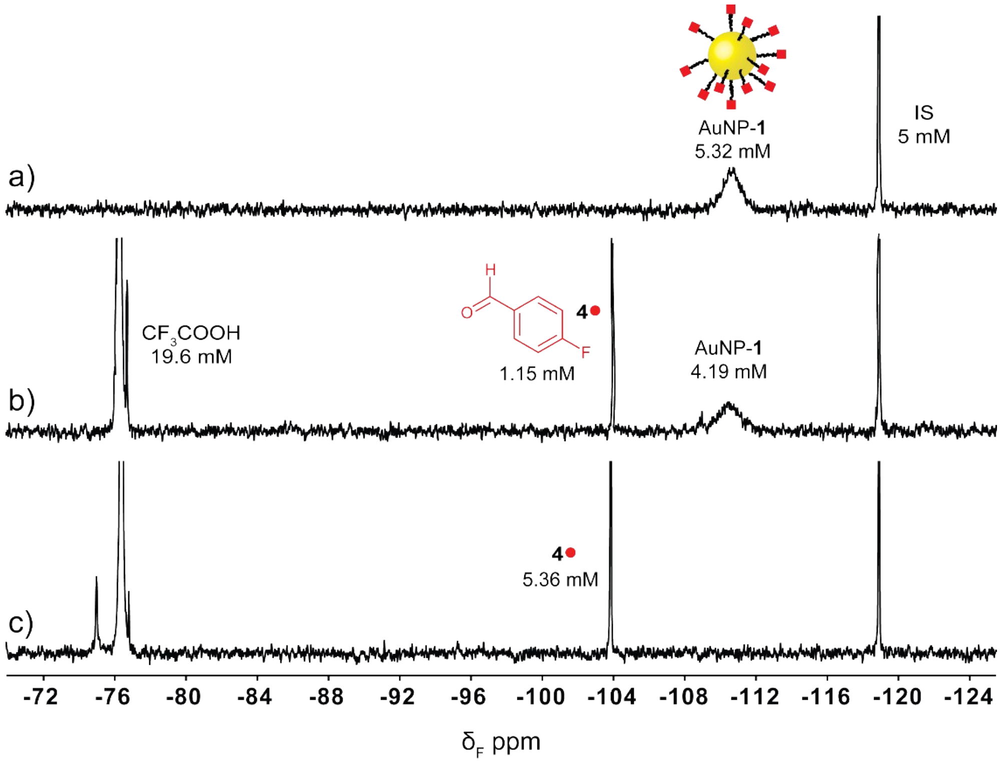

Figure S4. *In situ* monitoring of dynamic covalent exchange from AuNP-**1** to AuNP-**5** by ^{19}F NMR (470 MHz, THF/CH_2Cl_2/D_2O, 16 scans, 25 s recycle delay time). a) AuNP-**1** (5.32 mM). b) Reaction mixture after addition of aldehyde **2** (26.3 mM) and CF_3COOH (19.6 mM), then incubation at 50 °C for 2.75 h, showing the release of aldehyde **4** into bulk solution and reduction in concentration of nanoparticle-bound **1**. c) Reaction mixture after 51 h at 50 °C, confirming 100% conversion of the nanoparticle-bound hydrazone according to the concentration of released aldehyde **4**. IS: internal standard (4-fluorotoluene, 5.00 mM).

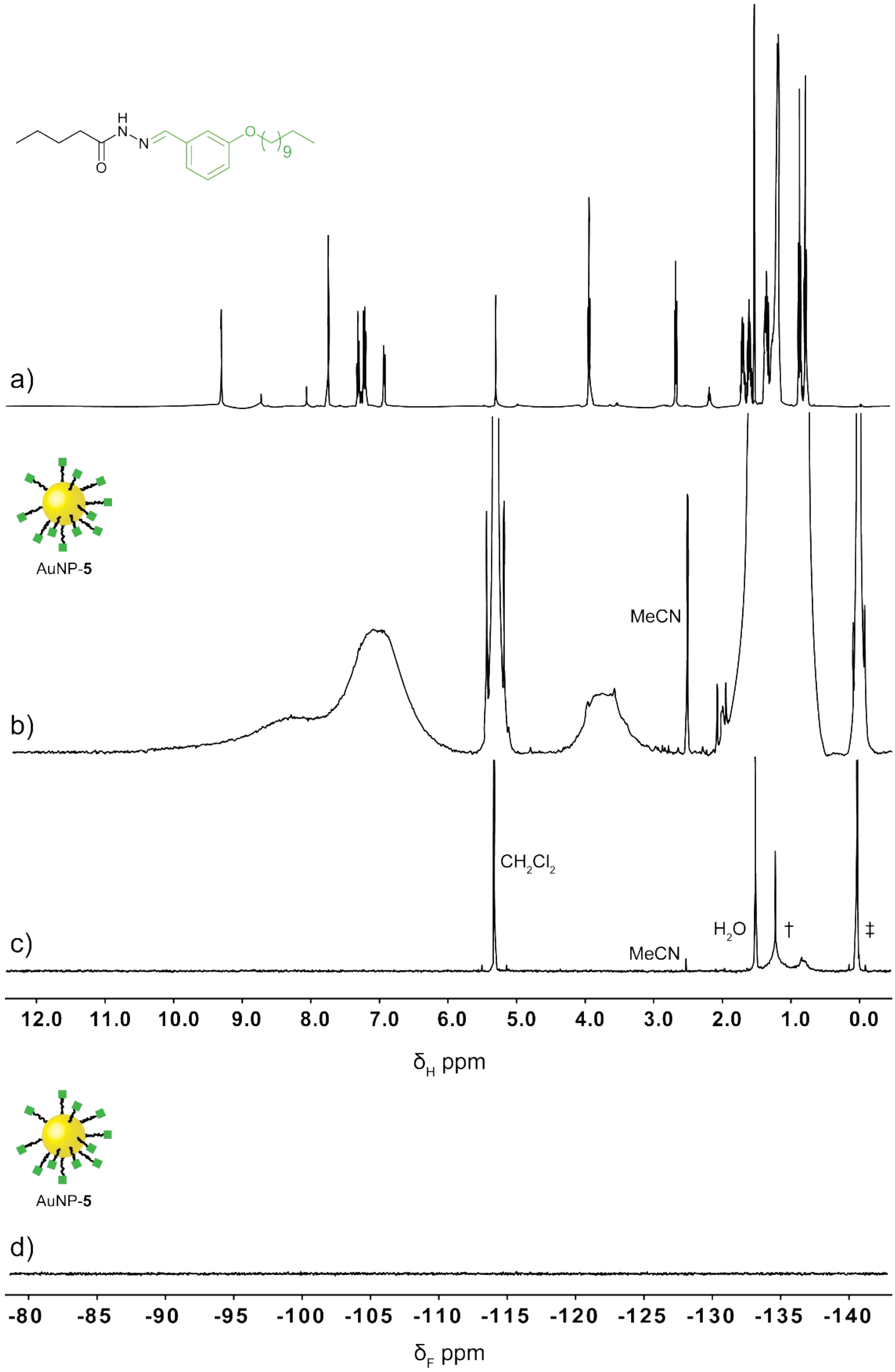

Figure S5. NMR Characterization of AuNP-**5**. a) ^{1}H NMR (500 MHz, CD_2Cl_2, 4 scans) spectrum of small molecule model compound corresponding to nanoparticle-bound **5**. b) ^{1}H NMR (700 MHz, CD_2Cl_2, 54 scans) spectrum of AuNP-**5**. c) T_2-Filtered ^{1}H NMR (500 MHz, CD_2Cl_2, 8 scans) spectrum of AuNP-**5** acquired using the CPMG-z pulse sequence.[4] All sharp signals can be assigned to residual non-deuterated solvents and impurities as indicated († = H grease: δ 0.84–0.87 (m), δ 1.25 (br s); ‡ = silicone grease: δ 0.07 (s)). d) ^{19}F NMR (470 MHz, CD_2Cl_2, 128 scans) spectrum of AuNP-**5**.

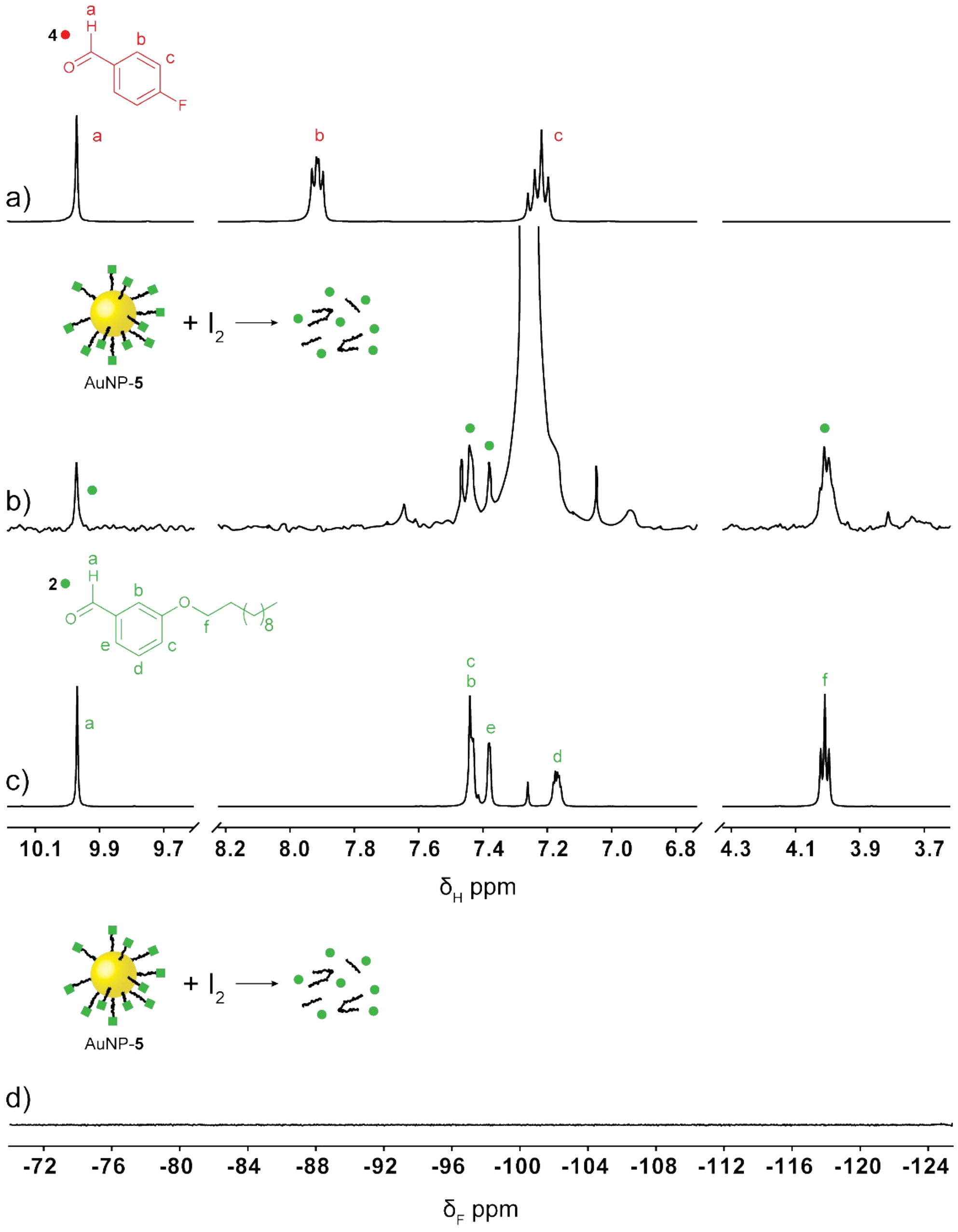

Figure S6. Oxidative ligand stripping from AuNP-**5**. a) ^{1}H NMR (500 MHz, $CDCl_3$, 8 scans) spectrum of aldehyde **4**. b) ^{1}H NMR (500 MHz, $CDCl_3$, 4 scans, 30 s recycle delay time) spectrum recorded 26 hours after addition of iodine to AuNP-**5**, showing the absence of aldehyde **4**, but signals corresponding to aldehyde **2** in bulk solution. c) ^{1}H NMR (400 MHz, $CDCl_3$, 8 scans) spectrum of aldehyde **2**. d) ^{19}F NMR (470 MHz, $CDCl_3$, 128 scans) spectrum recorded 26 hours after addition of iodine to AuNP-**5** showing the absence of fluorinated species in bulk solution.

4.2 Single-component monolayer AuNP-6

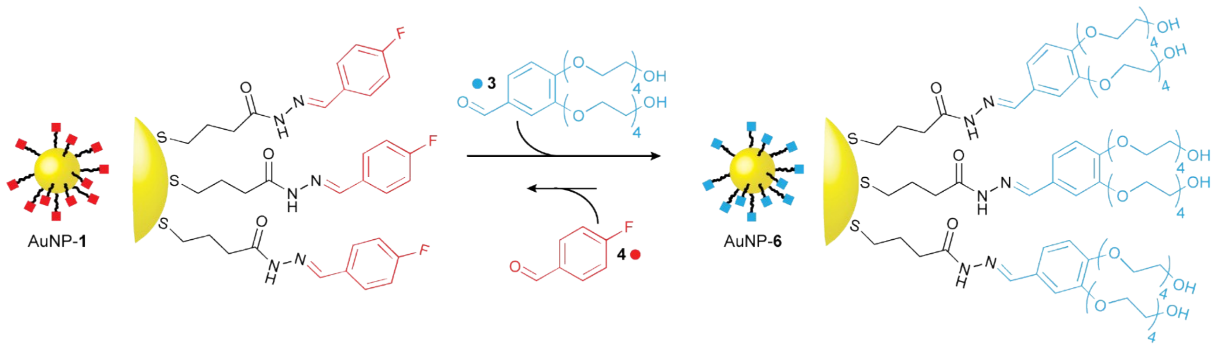

Concentrations of all fluorine-containing species were determined by quantitative ^{19}F NMR in the presence of 4-fluorotoluene as an internal standard of known concentration.

A stock solution of CF_3CO_2H was prepared in THF/D_2O (9:1) with 4-fluorotoluene as internal standard (5.00 mM) and concentration measured as 0.920 M.

A solution containing AuNP-**1** (7.2 mg) and 4-fluorotoluene (5.00 mM) was prepared in THF/D_2O (9:1, 600 μL) giving 5.07 mM in terms of 4-fluorobenzylidine hydrazone (3.04 μmol). To this was added aldehyde **3** (7.46 mg, 15.2 μmol), followed by an aliquot of the CF_3CO_2H stock solution (16.5 μL, 15.2 μmol), giving final concentrations of AuNP-**1** (4.93 mM), **3** (24.7 mM, 5.0 equiv.), and CF_3CO_2H (24.7 mM). This mixture was heated to 50 °C. After 16 h, ^{19}F NMR analysis revealed disappearance of the signal for nanoparticle-bound **1** and quantitative conversion to a sharp signal for unbound aldehyde **4**. A small quantity (< 5%) of 4-fluorobenzoic acid was also observed, and included in the calculation of total 4-fluorobenzylidine hydrazone conversion (4.47 mM, 91% conversion). However, a gel-like precipitate was observed at this stage and no further change in composition occurred over the next 7 h. Addition of D_2O (150 μL) brought all components back into solution and the mixture left at 50 °C for a further 19 h. After this time, ^{19}F NMR analysis indicated 100% conversion of the nanoparticle-bound hydrazone according to the concentration of released aldehyde **4** (Figure S7b). A further 5 equivalents aldehyde **3** were added to ensure complete conversion: no change was observed over a further 20 h at 50 °C.

The mixture was cooled to room temperature, and nanoparticle precipitation induced by addition of Et_2O/MeCN (7:1, 10 mL), followed by sonication (10 min, 20 °C), and centrifugation (1935×g rcf, 10 min, –4 °C). The colourless supernatant was carefully discharged and washing repeated in this manner a further twice. Further washes were conducted by dissolving the residue in the minimum volume of either MeOH or MeCN, then adding Et_2O/cyclohexane to induce nanoparticle precipitation, followed by sonication (10 min, 20 °C), centrifugation (1935×g rcf, 10 min, –4 °C) and removal of the supernatant until NMR analysis revealed all unbound species had been removed. Traces of volatile solvents were removed from the purified residue under a stream of compressed air, 1 mL water added, and the sample freeze dried to provide quantitatively exchanged AuNP-**6** (4.9 mg).

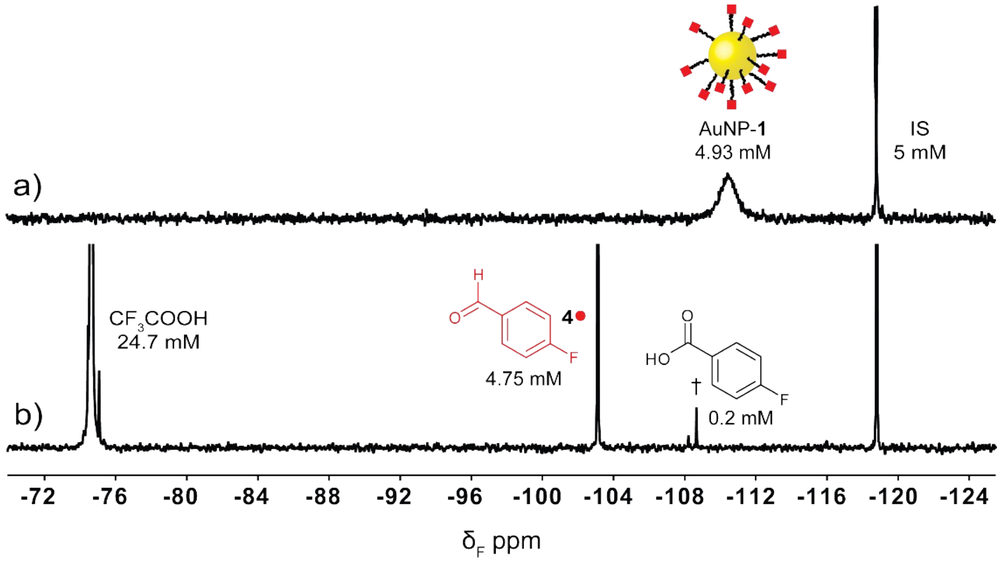

Figure S7. *In situ* monitoring of dynamic covalent exchange from AuNP-**1** to AuNP-**6** by ^{19}F NMR (470 MHz, THF/D_2O, 16 scans, 25 s recycle delay time). a) AuNP-**1** (4.93 mM). b) Reaction mixture after addition of aldehyde **3** (24.7 mM) and CF_3COOH (24.7 mM), then incubation at 50 °C for 42 h, confirming 100% conversion of the nanoparticle-bound hydrazone according to the concentration of released aldehyde **4** and 4-fluorobenzoic acid (†). IS: internal standard (4-fluorotoluene, 5.00 mM).

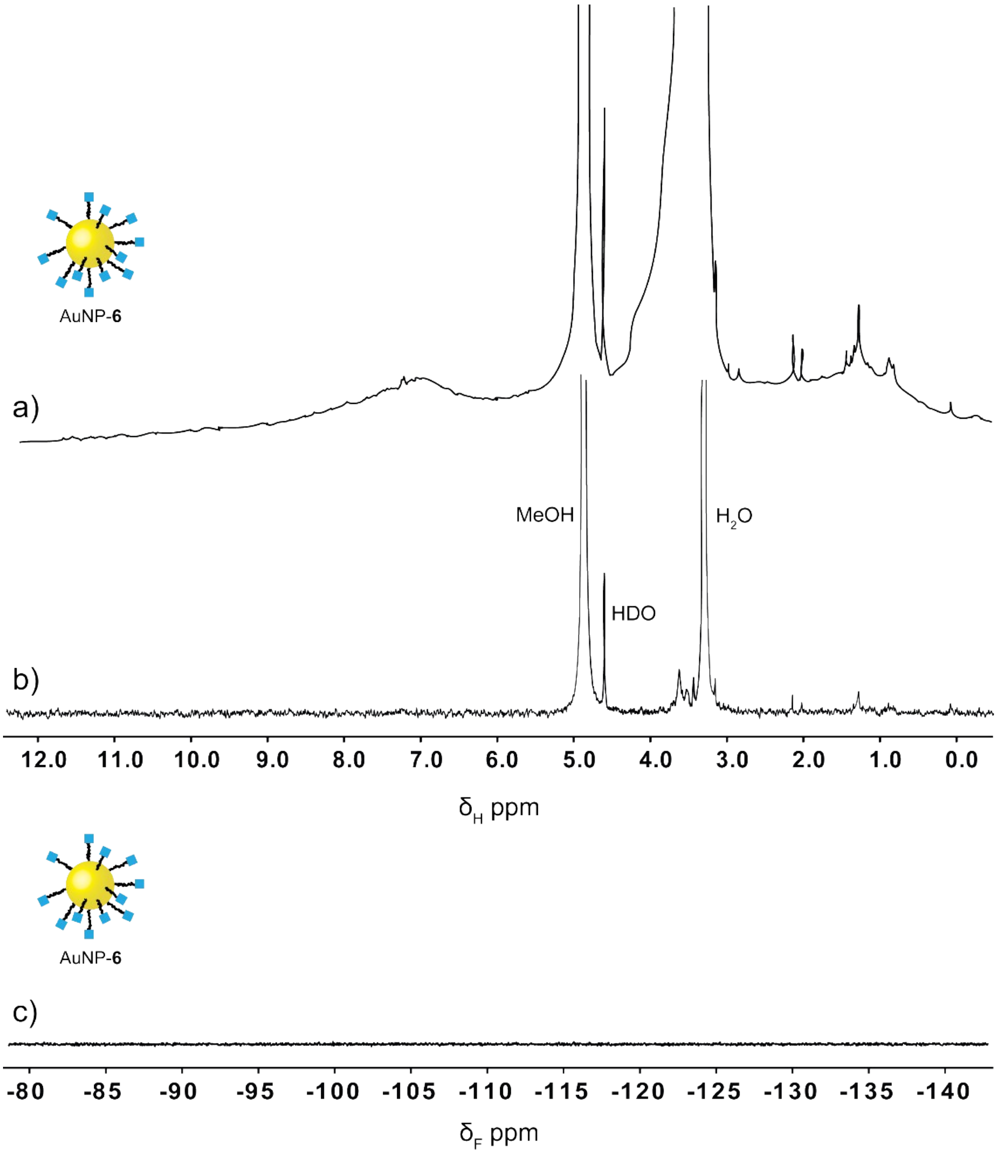

Figure S8. NMR Characterization of AuNP-**6**. a) ^{1}H NMR (500 MHz, CD_3OD, 48 scans) spectrum of AuNP-**6.** b) T_2-Filtered ^{1}H NMR (500 MHz, CD_3OD, 16 scans) spectrum of AuNP-**6** acquired using the CPMG-z pulse sequence.[4] All sharp signals can be assigned to residual non-deuterated solvents as indicated. c) ^{19}F NMR (377 MHz, CD_3OD, 128 scans) spectrum of AuNP-**6**.

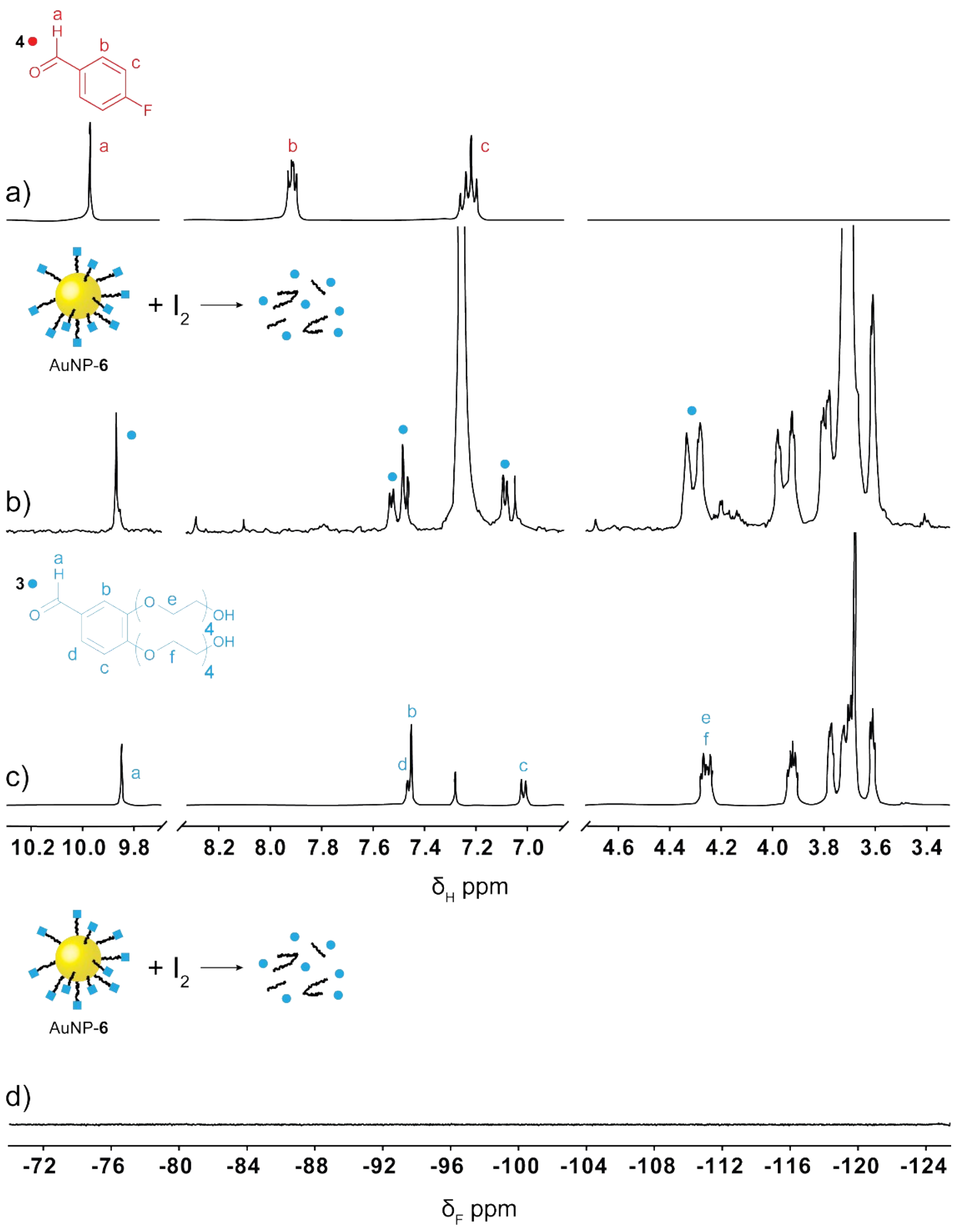

Figure S9. Oxidative ligand stripping from AuNP-**6**. a) ^{1}H NMR (500 MHz, $CDCl_3$, 8 scans) spectrum of aldehyde **4**. b) ^{1}H NMR (500 MHz, $CDCl_3$, 4 scans, 30 s recycle delay time) spectrum recorded 24 hours after addition of iodine to AuNP-**6**, showing the absence of any aldehyde **4** but signals corresponding to aldehyde **3** in bulk solution. c) ^{1}H NMR (500 MHz, $CDCl_3$, 8 scans) spectrum of aldehyde **3**. d) ^{19}F NMR (470 MHz, $CDCl_3$, 128 scans) spectrum recorded 24 hours after addition of iodine to AuNP-**6,** showing the absence of fluorinated species in bulk solution.

4.3 Continuum tuning of monolayer composition

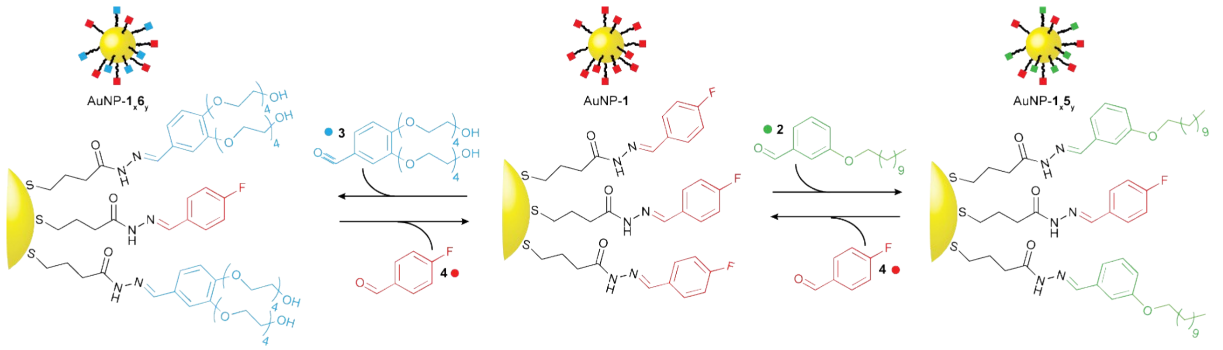

To prepare a range of mixed-monolayer nanoparticle samples, a series of exchange reactions was performed with differing stoichiometric excess of aldehyde **2** or **3**. Tetrahydrofuran/D_2O (9:1) was employed as reaction solvent as this mixture gave the best compromise of solubilities covering most monolayer compositions. To ensure the reaction endpoint was reached, each reaction was allowed to proceed for 3 days at 50 °C, although in practice, the reactions tended to occur significantly faster than this (see Sections 4.1 and 4.2).

Stock solutions were prepared as follows. Solutions **B** and **D** were prepared volumetrically to give accurately known concentrations. Concentrations for solutions **C** were determined by independent ^{1}H NMR experiments relative to 4-fluorotoluene as a volumetrically added internal standard; the concentration of nanoparticle solution **A** was determined by ^{19}F NMR during preparation of each sample (see below).

Stock solution A. Nanoparticles in THF; ca. 20 mg mL^{-1}.

Stock solution B. Internal standard (4-fluorotoluene) in THF at 48.0 mM.

Stock solution C2 or C3. Aldehyde (**2** or **3**) in THF at ca. 10 mM.

Stock solution D. Trifluoroacetic acid in THF at 240 mM.

Dynamic covalent exchange was performed at nanoparticle-bound hydrazone concentrations ca. 5–8 mM (corresponding to ca. 12–16 mg mL^{-1}) in THF/D_2O (9:1, 600 μL), with differing stoichiometries of aldehyde, in the presence of CF_3CO_2H (20 mM) and 4-fluorotoluene internal standard (4.0 mM). Each experiment was performed as follows.

An aliquot of nanoparticle solution (**A**, 390 μL) was taken in a NMR tube, and to this was added internal standard (**B**, 50 μL) and D_2O (60 μL). The concentration of nanoparticle-bound hydrazones was assessed at this stage by ^{19}F NMR. To this solution was then added appropriate volumes of stock solution **C** and THF to give the desired number of equivalents of aldehyde, and a sample volume of 550 μL. Finally, trifluoroacetic acid (**D**, 50 μL) was added.

The NMR tube was then held at 50 °C with occasional agitation by sonication for 3 d, recording NMR spectra at intermediate time points in order to track reaction progress (selected samples were monitored for a further 2 d but showed no further reaction during this period). After this time, the reaction solution was transferred to a vial and volatiles removed under a stream of compressed air. The residue was purified by repeated washings where the solid is suspended in a poor solvent, nanoparticles collected by centrifugation and the supernatant discarded, repeating this procedure until no unbound molecular species were detected by TLC or ^{1}H NMR analysis.

5. Determination of mixed monolayer composition

The concentration of 4-fluorobenzaldehyde (**4**) released during dynamic covalent hydrazone exchange was determined by deconvolution of the signal areas for **4** and the internal standard in the ^{19}F NMR spectrum taken prior to sample purification. Comparison to the initial concentration of nanoparticle-bound hydrazone **1** (also determined by ^{19}F NMR, see section 4.3) gave an initial assessment of the extent of exchange (% **4** released, Tables S1, S2).

In this study and others, we consistently observe that, under the conditions employed here, hydrazones of the general structure of **1**/**5**/**6** are stable with respect to hydrolysis, and so the proportion of aldehyde **4** released during exchange should be expected to provide a good estimate of the final monolayer composition (i.e. the final monolayer should incorporate negligible free hydrazide species). In order to verify this assumption, and to compare samples pre- and post-purification, the purified AuNP-$\mathbf{1}_x\mathbf{5}_y$ and AuNP-$\mathbf{1}_x\mathbf{6}_y$ samples were independently characterized by one of the two methods below.

Monolayer composition determination by oxidative ligand stripping

Nanoparticles in $CDCl_3$ (ca. 2 mg mL^{-1}) were treated with I_2 (ca. 1 mg), leading to oxidative decomposition of the sample, releasing the hydrazones from the surface as disulfides. These subsequently decompose to give the corresponding aldehydes in solution. To ensure there was no effect of preferential reaction for one hydrazone over the other, ^{1}H NMR spectra were recorded after 2 h and 24 h. At each time point, signals corresponding to each aldehyde component could be identified, allowing the original molar ratio of hydrazones to be established by area deconvolution.

For the series AuNP-$\mathbf{1}_x\mathbf{5}_y$ the ratios of components measured at both time points were in excellent agreement (see Figure S10 for a representative example of this procedure applied to AuNP-$\mathbf{1}_{0.6}\mathbf{5}_{0.4}$). For sample AuNP-$\mathbf{1}_{0.1}\mathbf{5}_{0.9}$-c, the monolayer composition was also verified in the presence of an internal standard. The amount of released **4** and **2** was tracked over time by ^{1}H NMR. Throughout the experiment, the relative concentrations of each component remained roughly constant. After 20 h, no further increase in overall aldehyde concentration was observed and the relative concentrations of **2** and **4** showed excellent agreement with the results obtained after either 2 h or 24 h in the experiment performed in the absence of the internal standard.

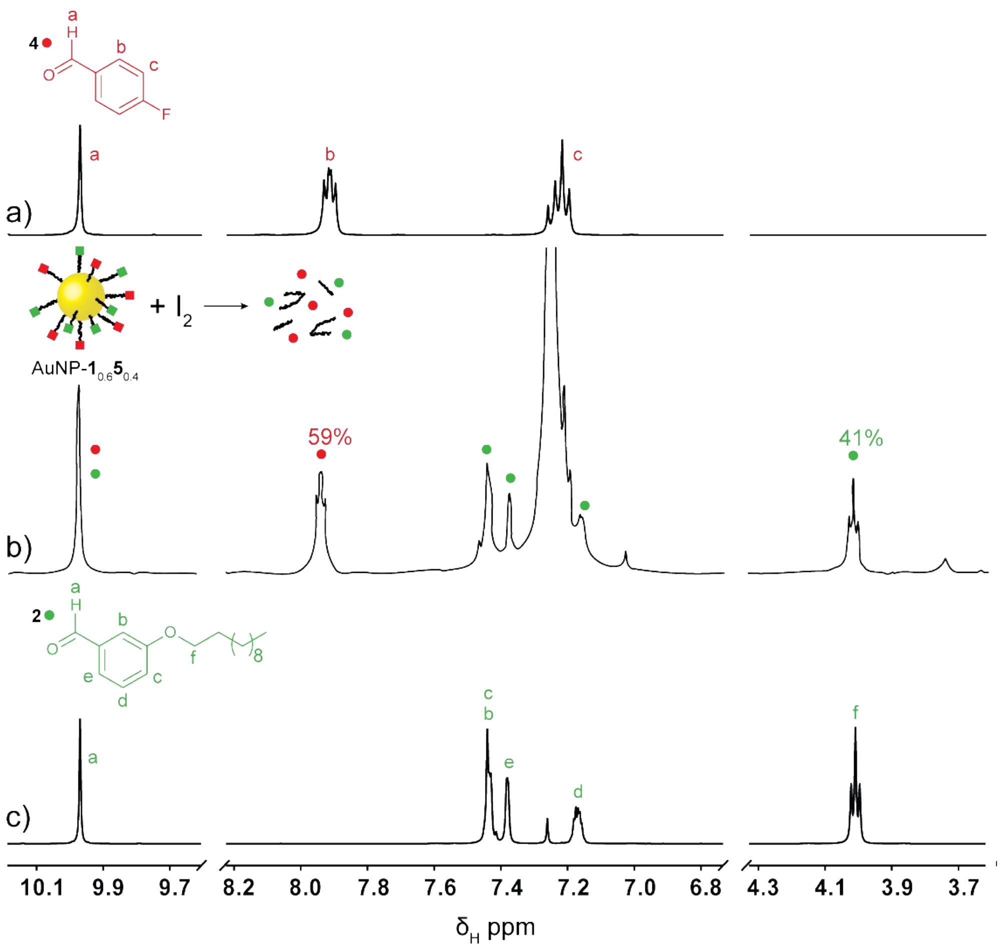

Figure S10. Example spectra for determination of mixed monolayer composition by oxidative ligand stripping (sample AuNP-$\mathbf{1}_{0.6}\mathbf{5}_{0.4}$). a) ^{1}H NMR (500 MHz, $CDCl_3$, 8 scans) spectrum of aldehyde **4**. b) ^{1}H NMR (500 MHz, $CDCl_3$, 32 scans, 30 s recycle delay time) spectrum recorded 24 hours after addition of iodine to AuNP-$\mathbf{1}_{0.6}\mathbf{5}_{0.4}$. The original monolayer composition could be established by area deconvolution of the signals corresponding to aldehydes **2** and **4** released in bulk solution. c) ^{1}H NMR (400 MHz, $CDCl_3$, 8 scans) spectrum of aldehyde **2**.

For samples AuNP-$\mathbf{1}_x\mathbf{6}_y$, inconsistent results were obtained in the presence of I_2 for spectra taken after different time periods, or depending on which signals were used for concentration determination. This was ascribed to side-reactions of aldehyde **3**, leading to multiple closely related species in solution. An alternative method was therefore developed for ascertaining monolayer composition on the purified nanoparticle samples. (It was subsequently determined that even for the AuNP-$\mathbf{1}_x\mathbf{6}_y$ series, careful identification of signals in the oxidative stripping experiment that are not affected by side reactions of **3** also provided very closely agreeing results from both quantification methods.)

Monolayer composition determination by exhaustive hydrazone exchange

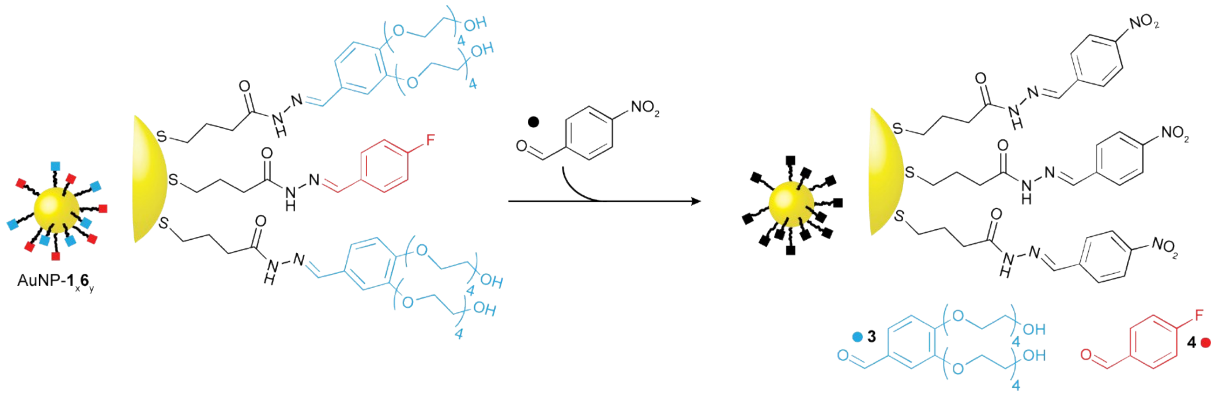

Samples were subjected to exhaustive hydrazone exchange using an excess of 4-nitrobenzaldehyde to drive the exchange reaction to completion while maintaining nanoparticle solubility throughout. Applying this procedure to AuNP-$\mathbf{1}_x\mathbf{5}_y$ samples gave very similar results to the oxidative stripping method. (See Figure S11 for this procedure applied to AuNP-$\mathbf{1}_{0.6}\mathbf{5}_{0.4}$, giving a very similar monolayer composition to that determined by oxidative ligand stripping from the same sample as shown in Figure S10. See Figure S12 for a representative example of this procedure applied to AuNP-$\mathbf{1}_{0.5}\mathbf{6}_{0.5}$).

Stock solution E. 4-Nitrobenzaldehyde (25 mM) and CF_3CO_2H (20 mM) in $CDCl_3$.

A dried portion (ca. 0.5 mg) of each nanoparticle sample was treated with stock solution **E** (600 μL). The mixture was sonicated for 20 minutes, then left at room temperature for 4 days. The ratios of released aldehydes **4**:**2**, or **4**:**3** were determined by signal deconvolution from the ^{1}H NMR spectrum.

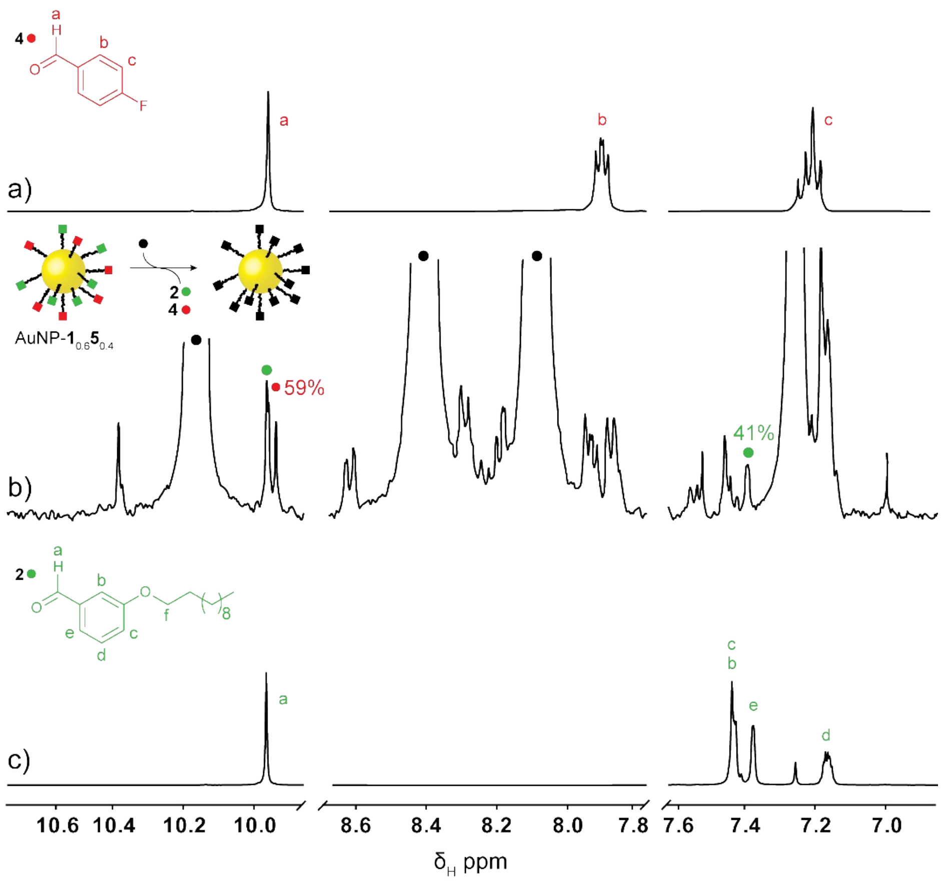

Figure S11. Example spectra for determination of mixed monolayer composition by exhaustive hydrazone exchange applied to sample AuNP-$\mathbf{1}_{0.6}\mathbf{5}_{0.4}$. a) ^{1}H NMR (500 MHz, $CDCl_3$, 8 scans) spectrum of aldehyde **4**. b) ^{1}H NMR (400 MHz, $CDCl_3$, 8 scans, 30 s recycle delay time) spectrum recorded on the reaction mixture containing AuNP-$\mathbf{1}_{0.6}\mathbf{5}_{0.4}$, 4-nitrobenzaldehyde (25 mM) and CF_3COOH (20 mM), after incubation at room temperature for 4 days. The original monolayer composition could be established by area deconvolution of the signals corresponding to aldehydes **2** and **4** released in bulk solution, and agrees very closely with the results from oxidative ligand stripping from the same sample (Figure S10). c) ^{1}H NMR (400 MHz, $CDCl_3$, 8 scans) spectrum of aldehyde **2**.

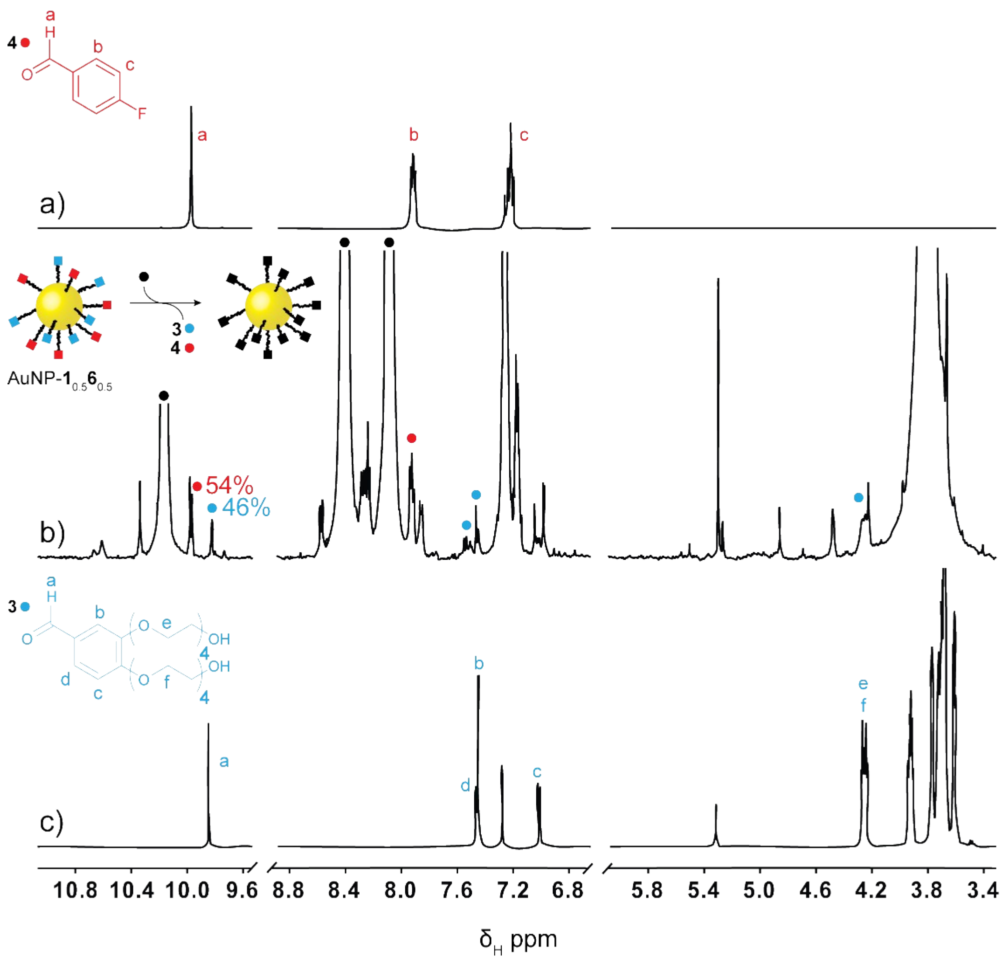

Figure S12. Example spectra for determination of mixed monolayer composition by exhaustive hydrazone exchange (applied to sample AuNP-**1**$_{0.5}$**6**$_{0.5}$. a) ^{1}H NMR (500 MHz, $CDCl_3$, 8 scans) spectrum of aldehyde **4**. b) ^{1}H NMR (500 MHz, $CDCl_3$, 4 scans, 30 s recycle delay time) spectrum recorded on the reaction mixture containing AuNP-**1**$_{0.5}$**6**$_{0.5}$, 4-nitrobenzaldehyde (25 mM) and CF_3COOH (20 mM), after incubation at room temperature for 4 days. The original monolayer composition could be established by area deconvolution of the signals corresponding to aldehydes **3** and **4** released in bulk solution. c) ^{1}H NMR (500 MHz, $CDCl_3$, 8 scans) spectrum of aldehyde **3**.

6. Assessment of nanoparticle solubility properties

Qualitative visual assessment of solubility

Solvent was added to a known mass of dried nanoparticles at a ratio of 0.5 mg mL^{-1}. The mixture was agitated by ultrasonication for 10 minutes, then subjected to centrifugation at 1935×g rcf for 2 minutes. Digital photographs of the resulting solutions and suspensions were collected immediately. Importantly, no obvious changes were subsequently observed by eye over a period of several days.

The images in Figure 2 show the solvents ordered according to the $E_T(30)$ parameter, which appears to give the closest correlation to nanoparticle solubility for all three single-component nanoparticle samples. This is consistent with the empirical solvent scale $E_T(30)$ offering a measure that combines both hydrogen bonding and electrostatic/polarizability contributions to solvent–solute interactions.[6] Other solvation scales such as relative permittivity or the Hildebrand solubility parameter fail to correctly predict nanoparticle behaviour in ethanol in particular (selected solvent scale data for the solvents used are provided in Table S3). Quantitative assessment of nanoparticle solubility in a wide range of solvents might provide further insight into the relevance of various solvent scales in describing nanoparticle solubilization, however this was beyond the scope of the current study.

Table S3. Solvation and polarity measures for the solvents employed in this study. Relative permittivities (ε_r),[7] dipole moments (μ),[7] $E_T(30)$ values,[8] and Hildebrand solubility parameters (δ_H).[9]

	ε_r	μ / D	$E_T(30)$ / kcal mol^{-1}	δ_H / $MPa^{1/2}$
n-Hexane	1.9	0.1	31	15
Carbon tetrachloride	2.2	0.0	32	18
Diethyl ether	4.3	1.2	35	15
Tetrahydrofuran	7.5	1.8	38	19
Dichloromethane	8.9	1.6	41	20
N,*N*-Dimethylformamide	38	3.8	43	24
Dimethylsulfoxide	47	4.0	45	27
Ethanol	25	1.7	52	26
Water	80	1.9	63	48

Quantitative assessment of saturation concentration and solubility by UV-Vis spectroscopy

Semi-quantitative assessment of nanoparticle saturated solution concentration was achieved by progressively diluting a saturated solution and monitoring the resulting changes in the UV-Vis absorption spectrum. A starting point solution was prepared by adding 100 μL solvent to a dried nanoparticle sample of known mass (ca. 5 mg). The mixture was than sonicated for 5 minutes and left to settle for a further 5 minutes, in each case a precipitate was clearly observed by eye. An aliquot of the supernatant (10 μL) was collected, diluted to 250 μL and the UV-Vis absorption recorded. The remaining stock solution was then diluted by adding 10 μL fresh solvent. The sonication, settling, sampling cycle was then repeated, recording a new spectrum each time. The point at which absorbance was observed to decrease in the expected linear fashion on dilution was taken as an estimation of the maximum saturated solution concentration (see Figure S13 for a representative example). In order to estimate the amount of material removed with each analysis aliquot, it was assumed that all nanoparticle material was homogeneously dispersed, which will most certainly not be the case at all points above saturation. As a consequence, this method over-estimates the amount of nanoparticle sample removed, and under-estimates the solution saturation concentration.

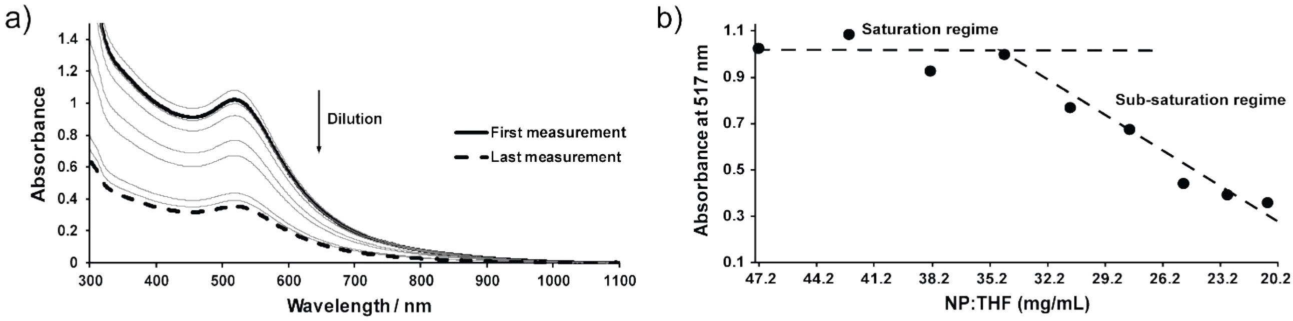

Figure S13. Assessment of saturated solution concentration for AuNP-**1** in THF: a) Series of UV-Vis spectra recorded during progressive dilution starting from a saturated solution of AuNP-**1**. b) Resulting absorbance values measured at 517 nm plotted against the estimated nanoparticle/solvent ratio concentration. The saturated solution concentration was then estimated as the point at which absorbance begins to decrease in the expected linear fashion on dilution.

Assessment of nanoparticle solubility in the solvents shown in Figure 3 was then achieved by measuring the absorbance at 517 nm for saturated solutions of each nanoparticle sample. To a sample of known mass, solvent was added to achieve a ratio calculated to be within the saturation regime as determined for the single-component monolayer samples described above (AuNP-$\mathbf{1}_x\mathbf{5}_y$: in *n*-hexane > 35 mg mL^{-1}; in DMF > 41 mg mL^{-1}. AuNP-$\mathbf{1}_x\mathbf{6}_y$: in THF > 34 mg mL^{-1}; in H_2O > 22 mg mL^{-1}). The suspension was than sonicated for 5 minutes and left to settle for a further 10 minutes. In each case a precipitate was clearly observed by eye. An aliquot of the supernatant (10 μL) was collected, diluted to 250 μL and the UV-Vis spectrum recorded against a blank of the appropriate pure solvent.

7. Solvophobic nanoparticle self-assembly

7.1 Assessment of solvodynamic size distributions by dynamic light scattering

Solvodynamic size was determined by dynamic light scattering in solvent mixtures of increasing polarity by titrating a nanoparticle solution in 100% anhydrous THF with a solution of the same nanoparticles in H_2O/THF (1:1, *v*/*v*), as described in the procedure below. At each titration point, three independent measurements were made in series, and the results averaged. In turn, each measurement is the average of 13 sequential scans. The solvodynamic sizes are reported in Figure 4 and Figure S14 as the mean for distributions expressed as % number of particles (plots of the distributions expressed as both % number of particles and % particle volume are shown below for selected samples). Size distributions were calculated by the instrument from the recorded intensity data using the appropriate values for viscosity, refractive index and dielectric constant estimated for each solvent composition as described below.

Results for the full range of solvent compositions investigated (0–50% H_2O/THF *v*/*v*) are shown in Figure S14, along with an expansion for the region 0–12% H_2O/THF (*v*/*v*) where well-dispersed nanoparticles are observed at all compositions prior to the sharp 'onset of aggregation' point, which is unique for each sample.

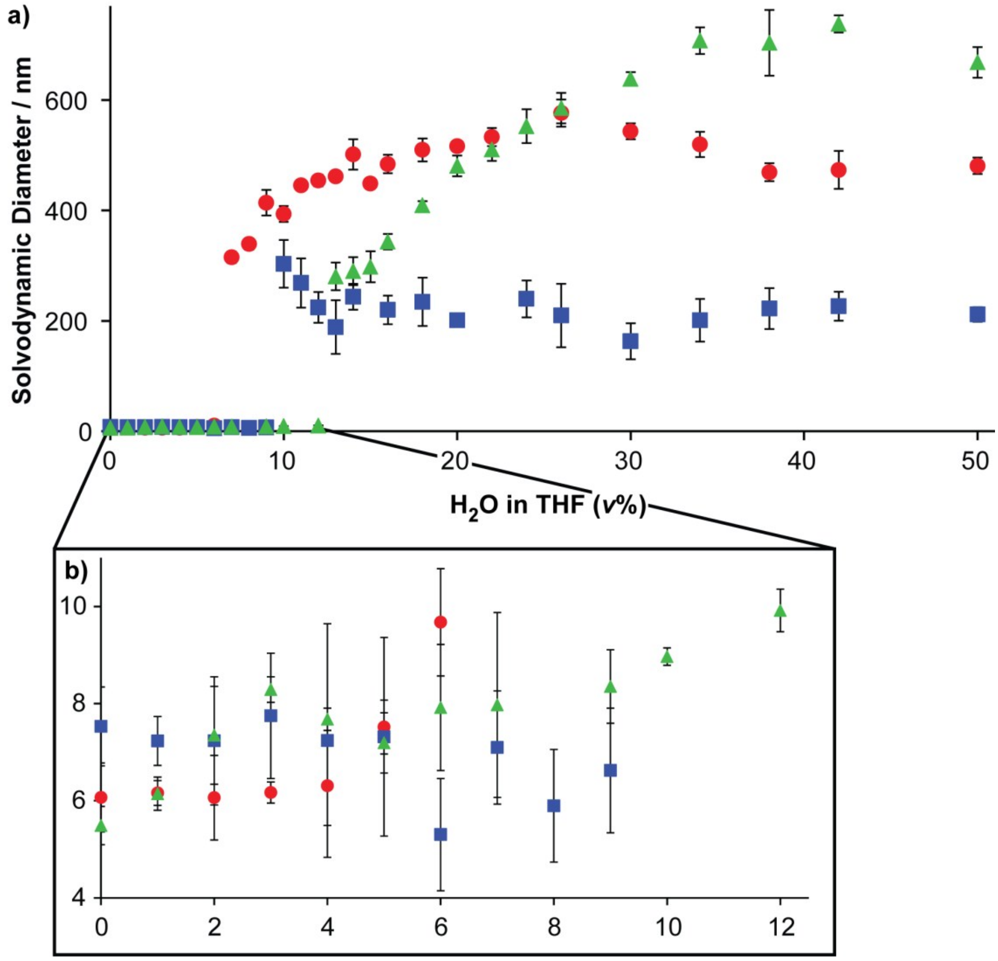

Figure S14. Solvodynamic size measurements by DLS on increasing solvent polarity in H_2O/THF mixtures for AuNP-**5** (red circles); AuNP-$\mathbf{1}_{0.1}\mathbf{5}_{0.9}$ (blue squares); AuNP-$\mathbf{1}_{0.3}\mathbf{5}_{0.7}$ (green triangles). (a) Full range of solvent compositions investigated: 0–50% H_2O/THF (*v*/*v*); (b) expansion for the compositions in the range 0–12% H_2O/THF (*v*/*v*). Error bars indicate ± 1 standard deviation from the mean.

Experimental procedure

A dried portion (ca. 0.4 mg) of each nanoparticle sample was dissolved in anhydrous THF (4 mL). The mixture was than sonicated for 10 minutes, filtered (Whatman Puradisc 13, polypropylene, 100 nm) and used to prepare the following stock solutions:

Stock solution F. Nanoparticles in anhydrous THF (0.05 mg mL^{-1}).

Stock solution G. Nanoparticles in THF/H_2O (1:1 *v*/*v*, 0.05 mg mL^{-1}).

An aliquot of stock solution **F** (1.5 mL) was transferred to a cuvette, allowed to equilibrate in the DLS instrument for 2 minutes, then the size distribution recorded. An appropriate volume of the measured sample was then removed and replaced with the same volume of solution **G**, so as to increase the amount of water by 1% without diluting the nanoparticles. The resulting solution was then left to equilibrate in the instrument for 2 minutes before re-analysis. The titration was continued until the solvent composition reached 1:1 THF/H_2O.

Estimation of solvent parameters for solvent mixtures

From the reported values for the neat solvents at 25 °C, solvent parameters for binary mixtures were estimated using equations reported in the literature for viscosity,[10] refractive index,[11] and dielectric constant.[12]

Solvodynamic size distributions

Solvodynamic size distribution for selected samples, expressed as both % particle numbers and % particle volume.

AuNP-**5** in 100% THF (non-aggregated)

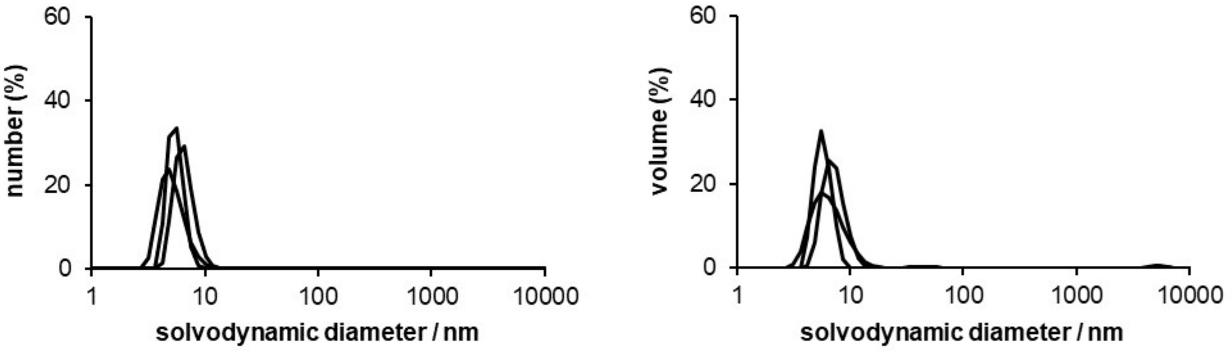

AuNP-**5** in 7% H_2O/THF (immediately after aggregation onset)

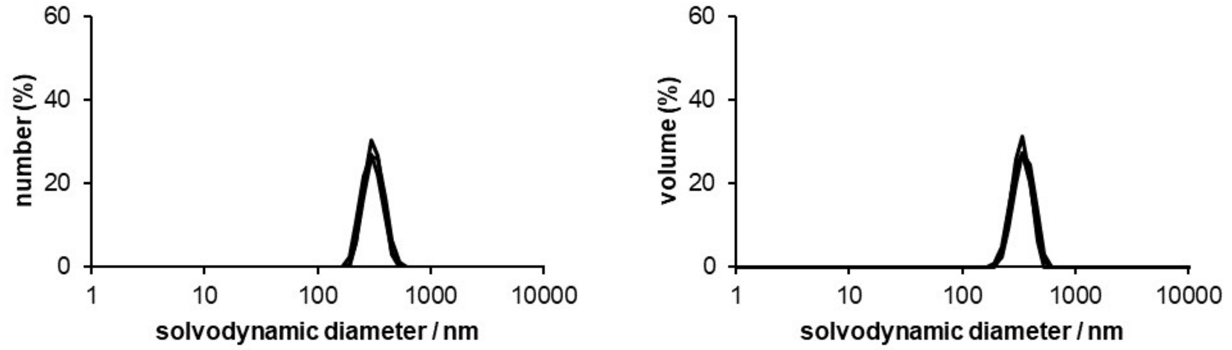

AuNP-**5** in 10% H_2O/THF (at first plateau in aggregate size)

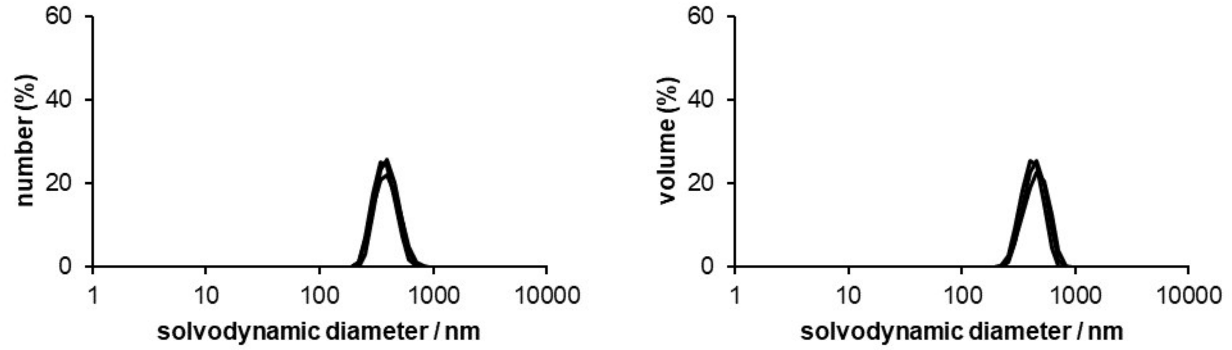

AuNP-$\mathbf{1}_{0.1}\mathbf{5}_{0.9}$ in 8% H_2O/THF (non-aggregated)

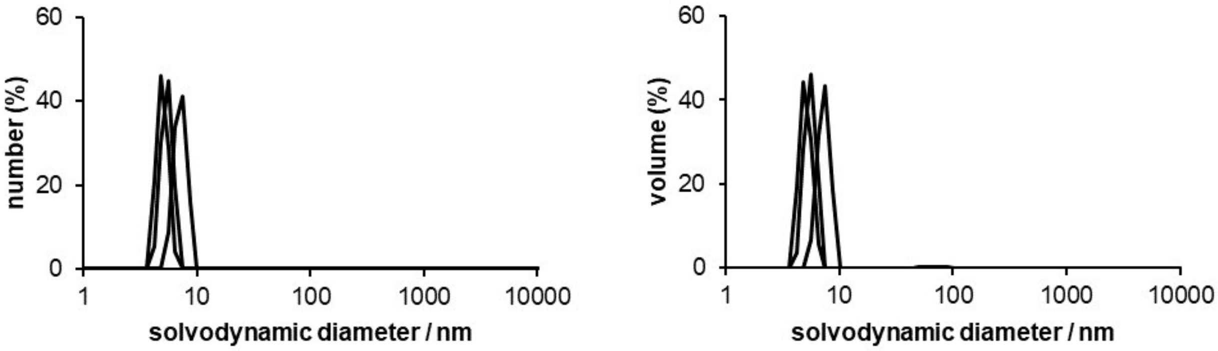

AuNP-$\mathbf{1}_{0.1}\mathbf{5}_{0.9}$ in 10% H_2O/THF (immediately after aggregation onset)

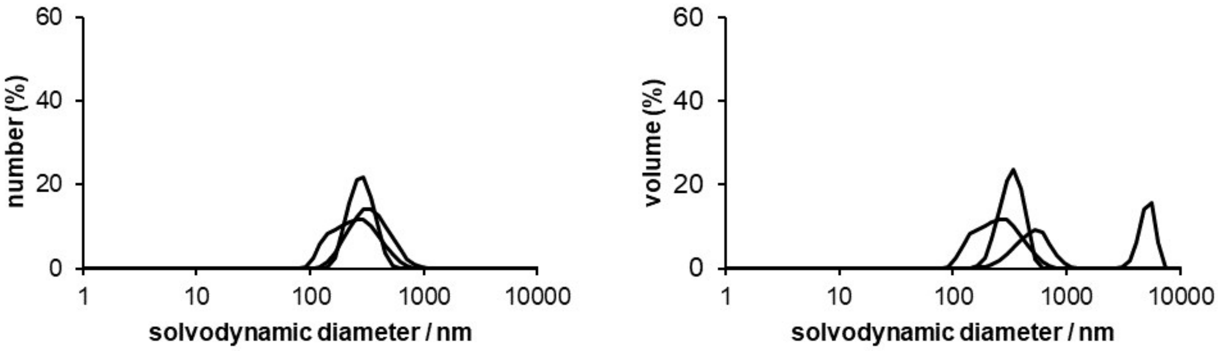

AuNP-$\mathbf{1}_{0.3}\mathbf{5}_{0.7}$ in 12% H_2O/THF (non-aggregated)

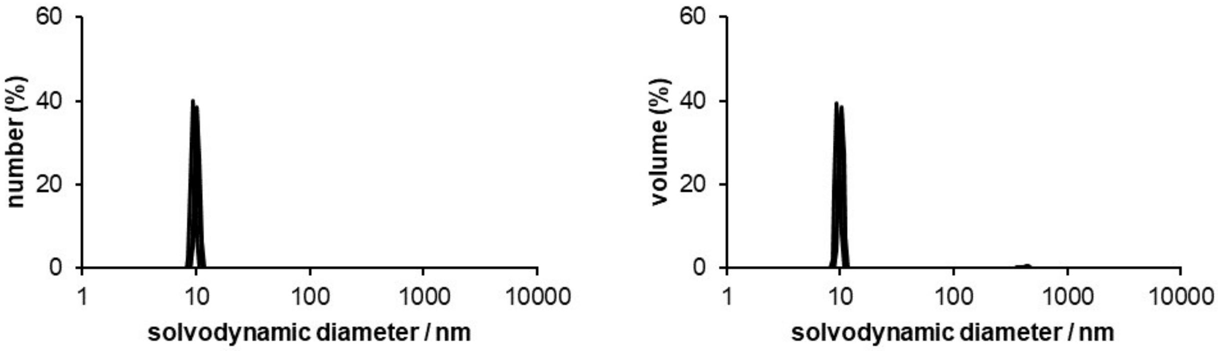

AuNP-$\mathbf{1}_{0.3}\mathbf{5}_{0.7}$ in 13% H_2O/THF (immediately after aggregation onset)

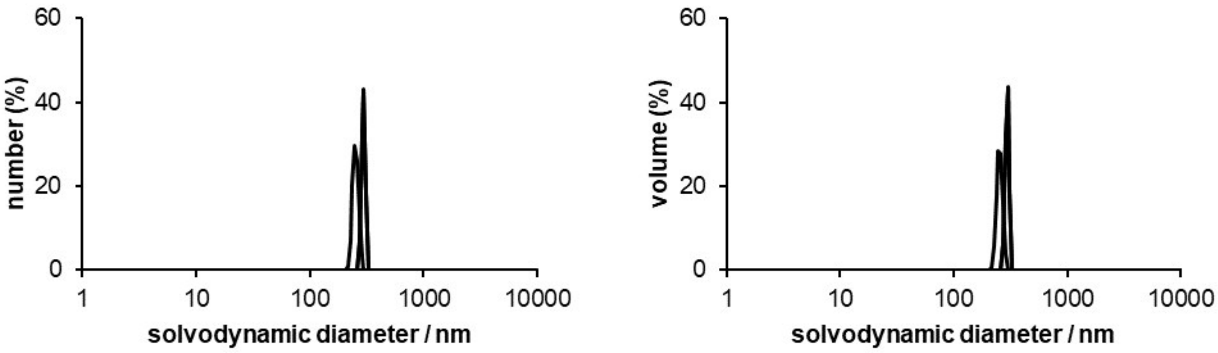

7.2 Supplementary TEM images characterizing solvophobic nanoparticle self-assembly

The TEM images below were all taken from grids prepared directly from the analogous solution sample described in Section 7.1. Immediately after analysis by DLS, one drop of nanoparticle suspension was dropped onto the TEM grid sitting on a lint-free tissue. The grids were left to dry at ambient pressure and temperature. Full solvodynamic size distributions for the corresponding solution-phase samples can be found in Section 7.1.

<u>AuNP-**5** in 100% THF (non-aggregated)</u>

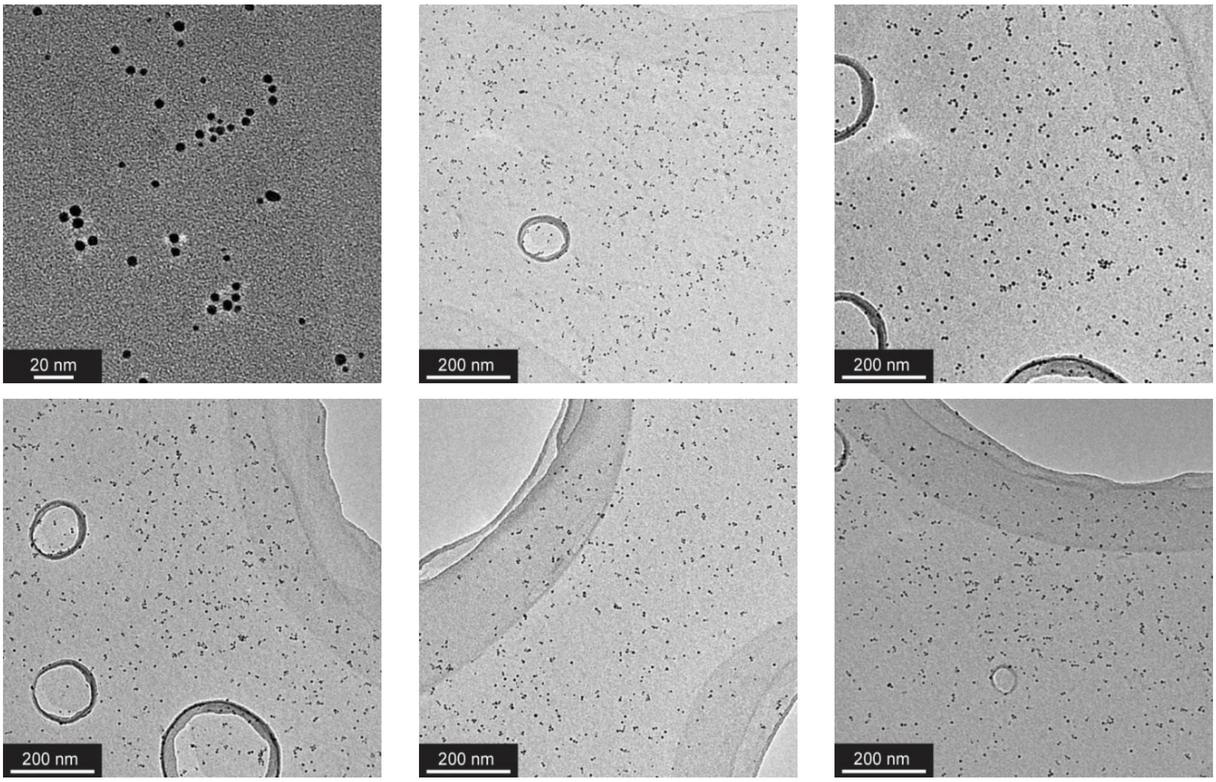

AuNP-**5** in 7% H_2O/THF (immediately after aggregation onset)

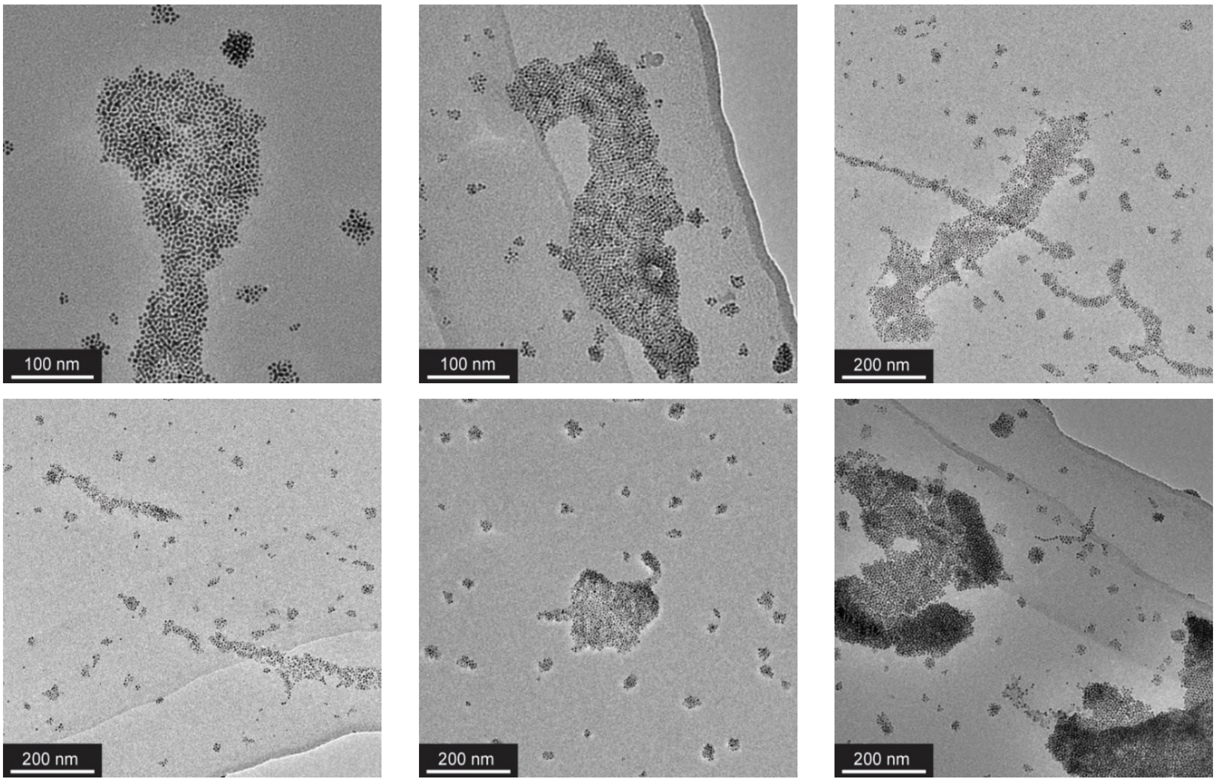

AuNP-**5** in 10% H_2O/THF (at first plateau in aggregate size)

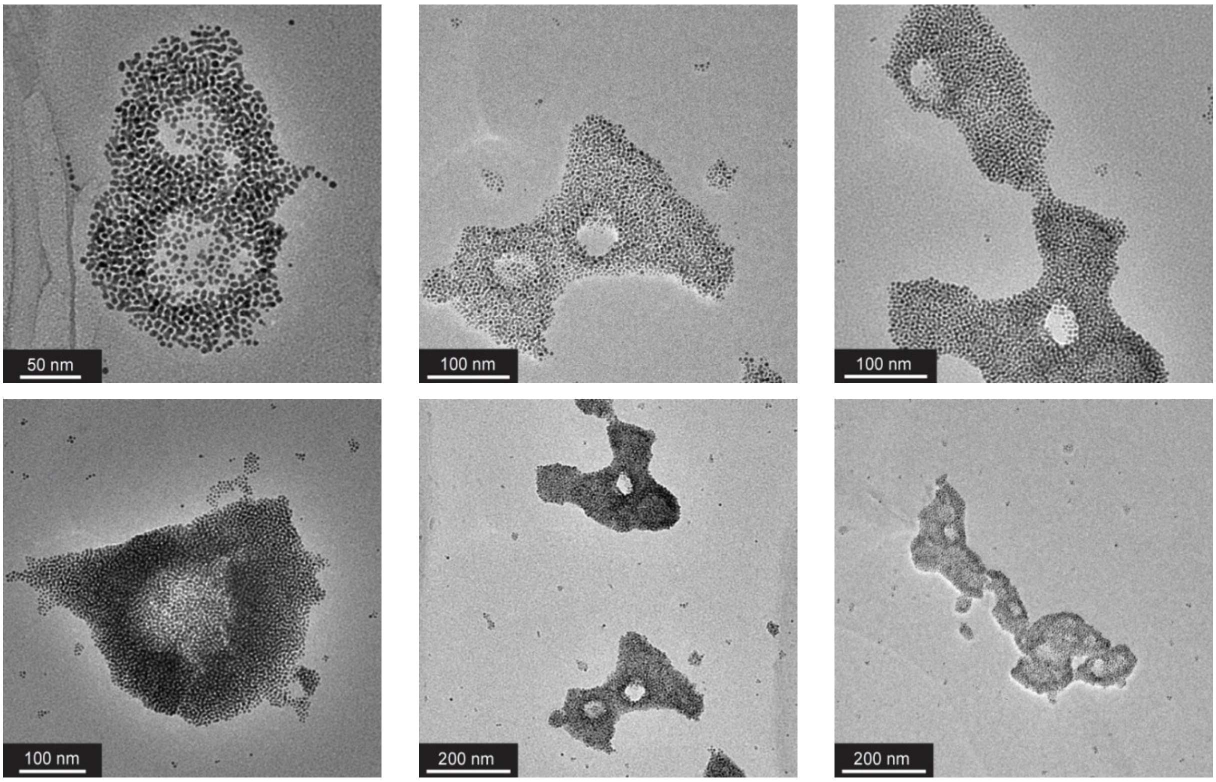

AuNP-$\mathbf{1}_{0.1}\mathbf{5}_{0.9}$ in 10% H_2O/THF (immediately after aggregation onset)

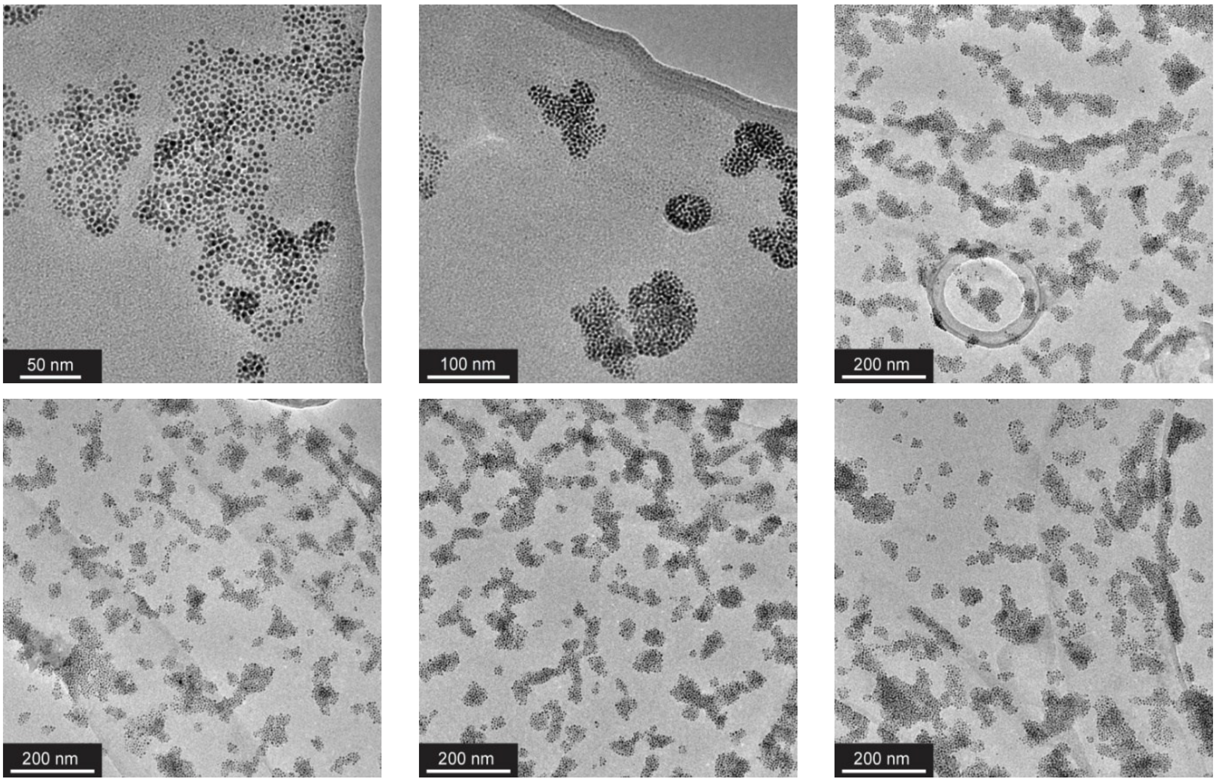

AuNP-$\mathbf{1}_{0.3}\mathbf{5}_{0.7}$ in 13% H_2O/THF (immediately after aggregation onset)

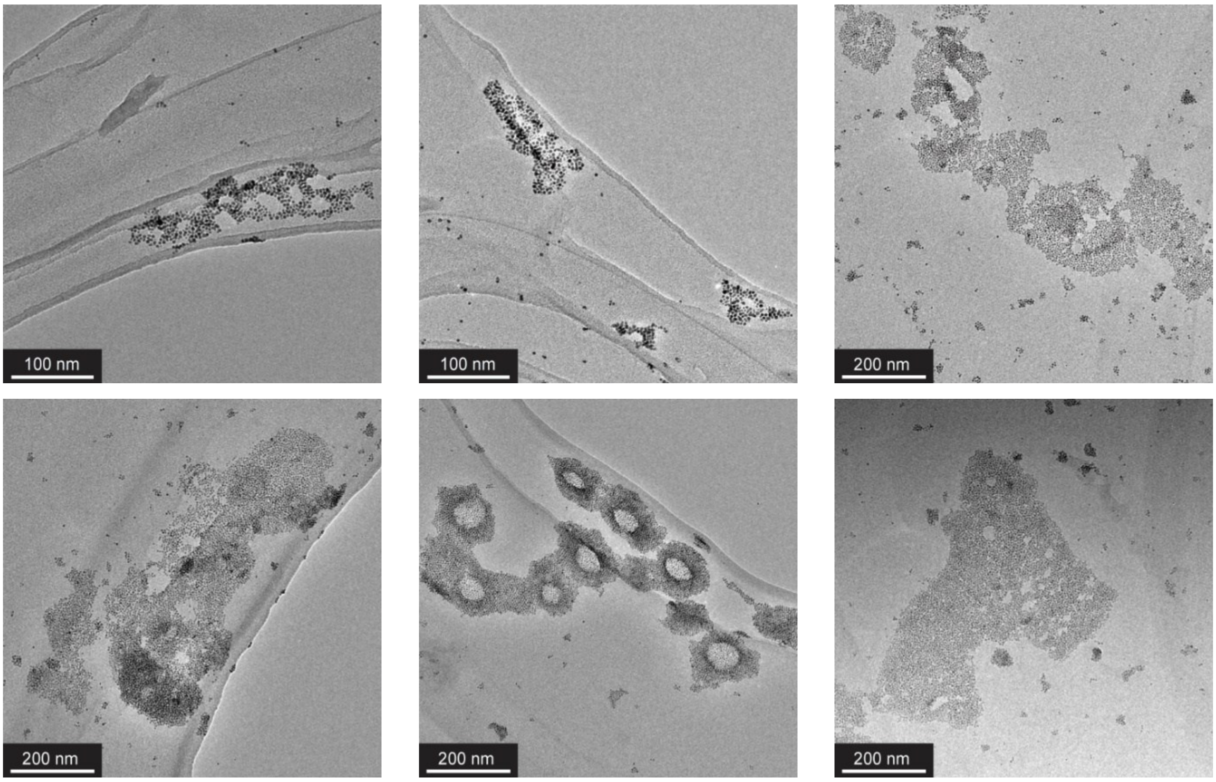

8. ^{1}H and ^{13}C NMR spectra of organic compounds

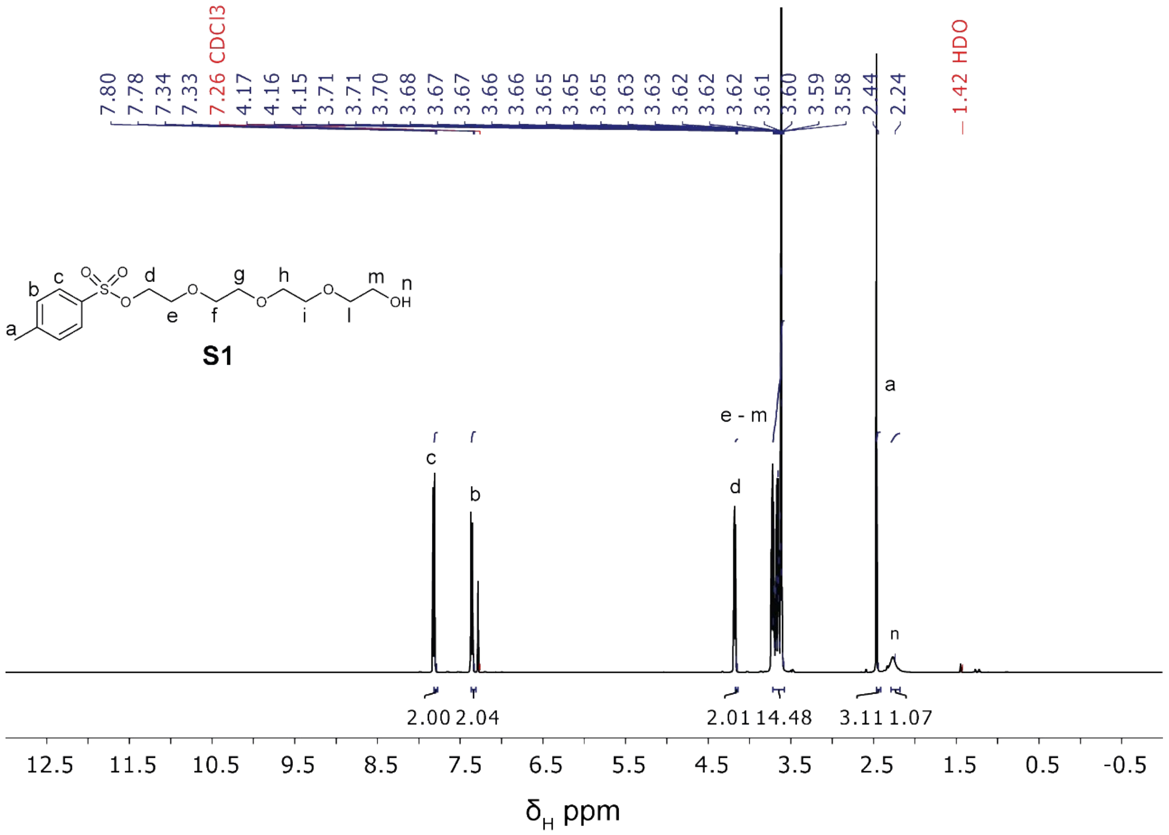

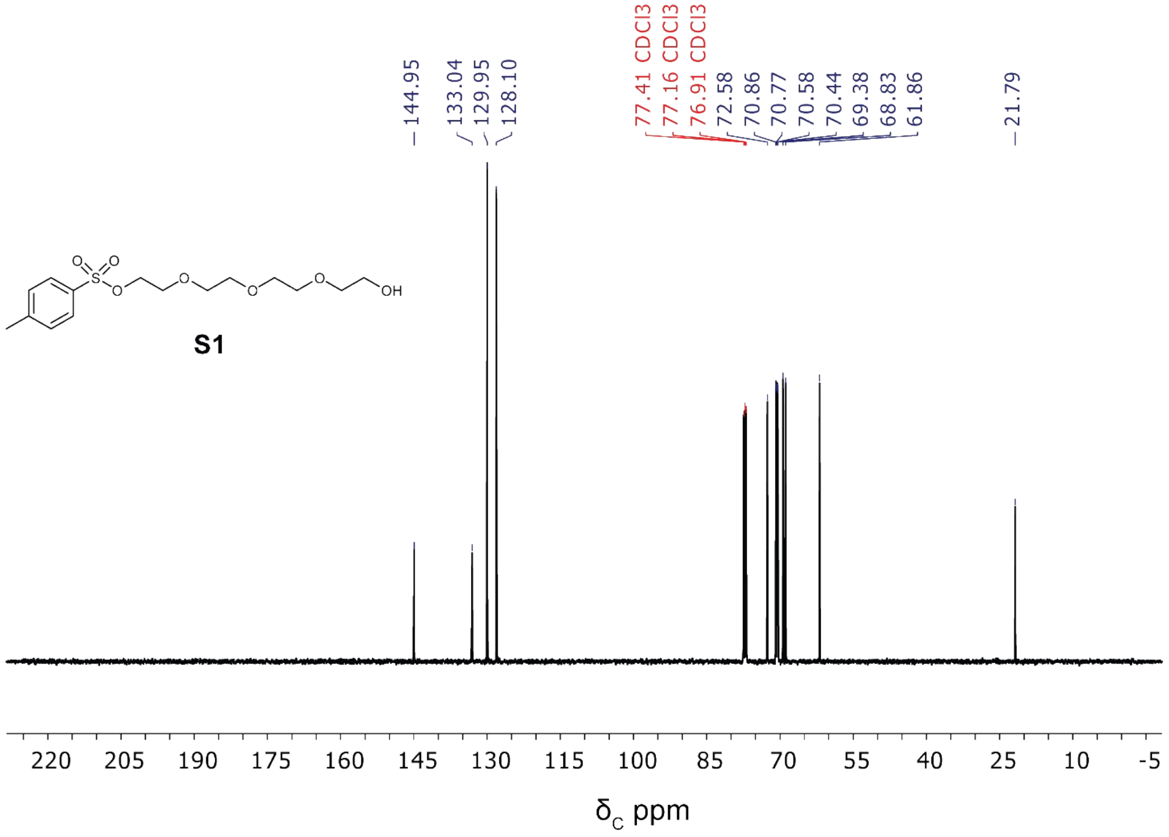

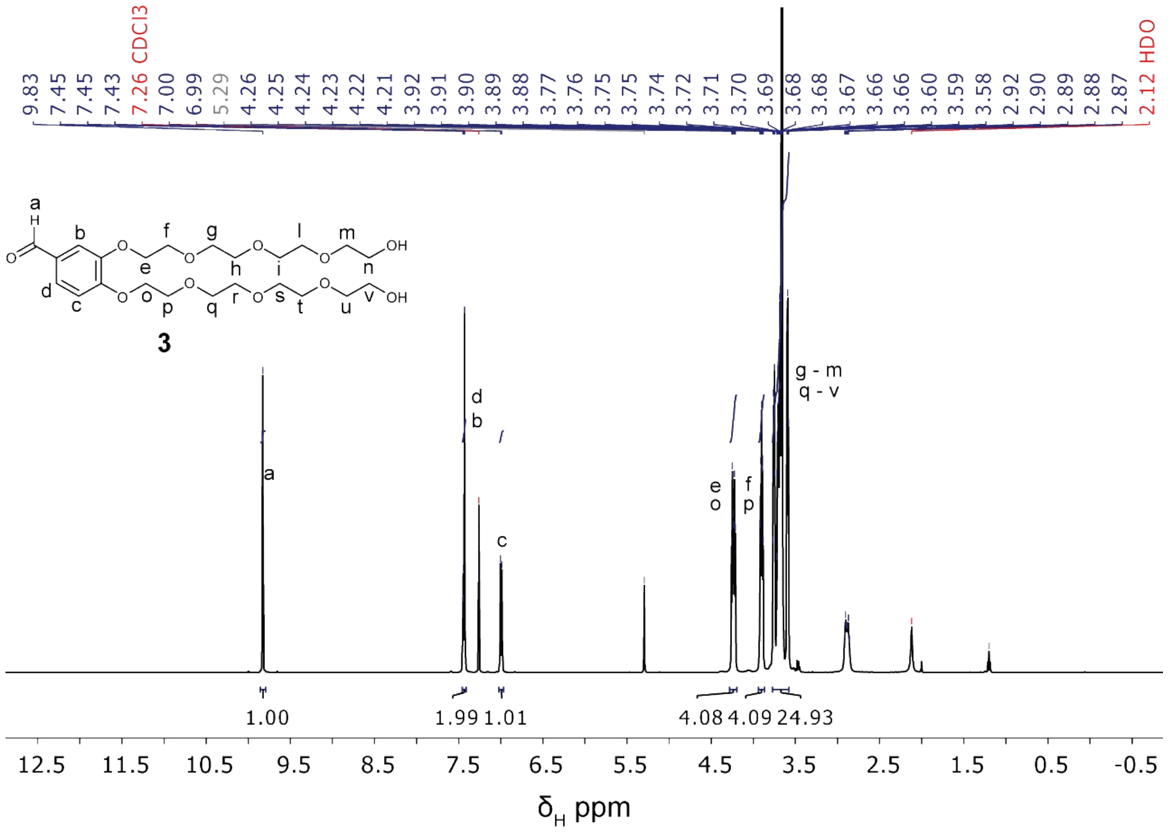
9.83
7.45
7.45
7.43
7.26 CDCl3
7.00
6.99
5.29
4.26
4.25
4.24
4.23
4.22
4.21
3.92
3.91
3.90
3.89
3.88
3.77
3.76
3.75
3.75
3.74
3.72
3.71
3.70
3.69
3.68
3.68
3.67
3.66
3.66
3.60
3.59
3.58
2.92
2.90
2.89
2.88
2.87
2.12 HDO
3
a
d
b
c
e
o
f
p
g - m
q - v
1.00
1.99
1.01
4.08
4.09
24.93
δH ppm

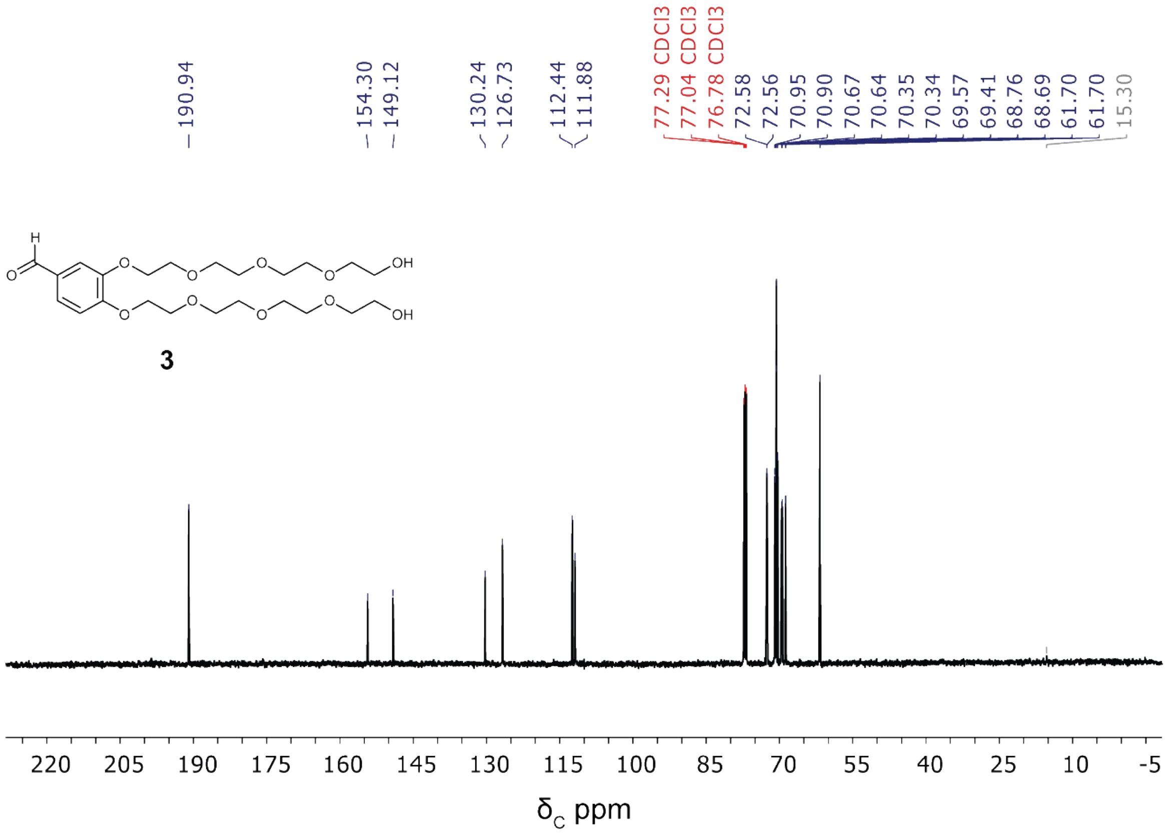
190.94
154.30
149.12
130.24
126.73
112.44
111.88
77.29 CDCl3
77.04 CDCl3
76.78 CDCl3
72.58
72.56
70.95
70.90
70.67
70.64
70.35
70.34
69.57
69.41
68.76
68.69
61.70
61.70
15.30
3
δC ppm

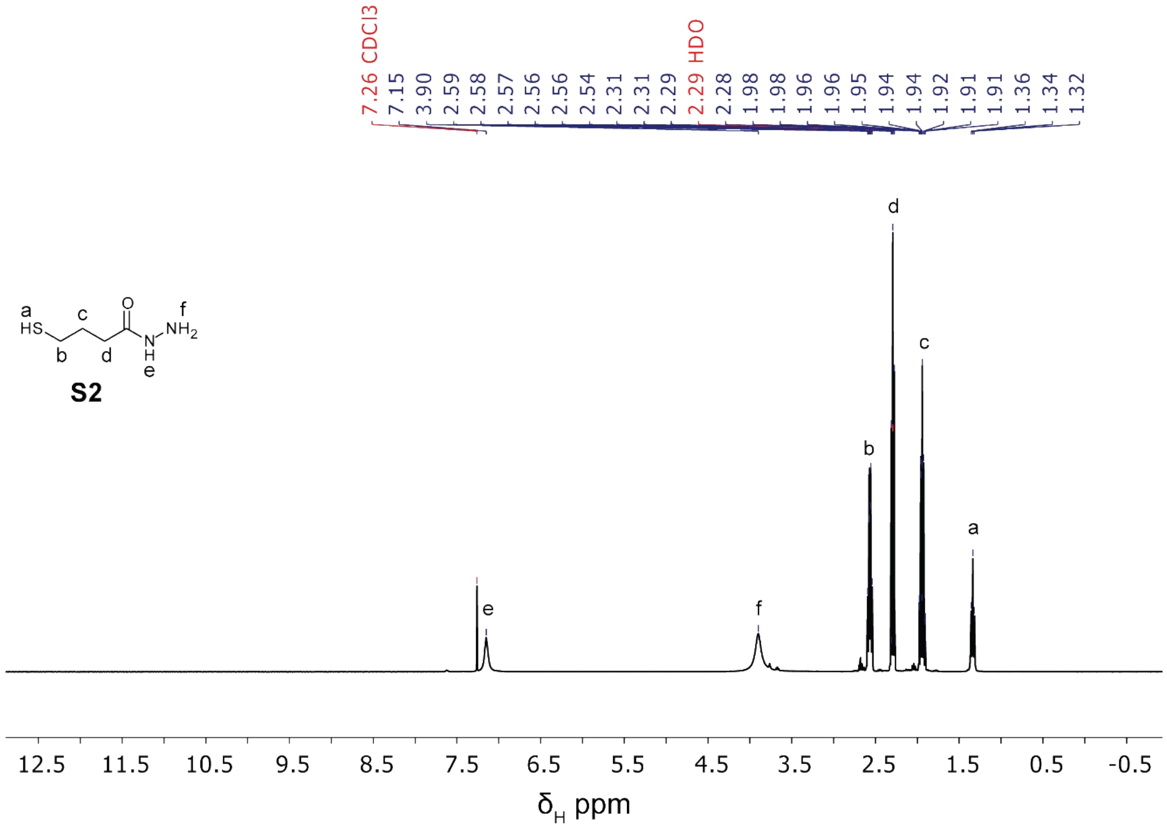
7.26 CDCl3
7.15
3.90
2.59
2.58
2.57
2.56
2.56
2.54
2.31
2.31
2.29
2.29 HDO
2.28
1.98
1.98
1.96
1.96
1.95
1.94
1.94
1.92
1.91
1.91
1.36
1.34
1.32
S2
δ_H ppm

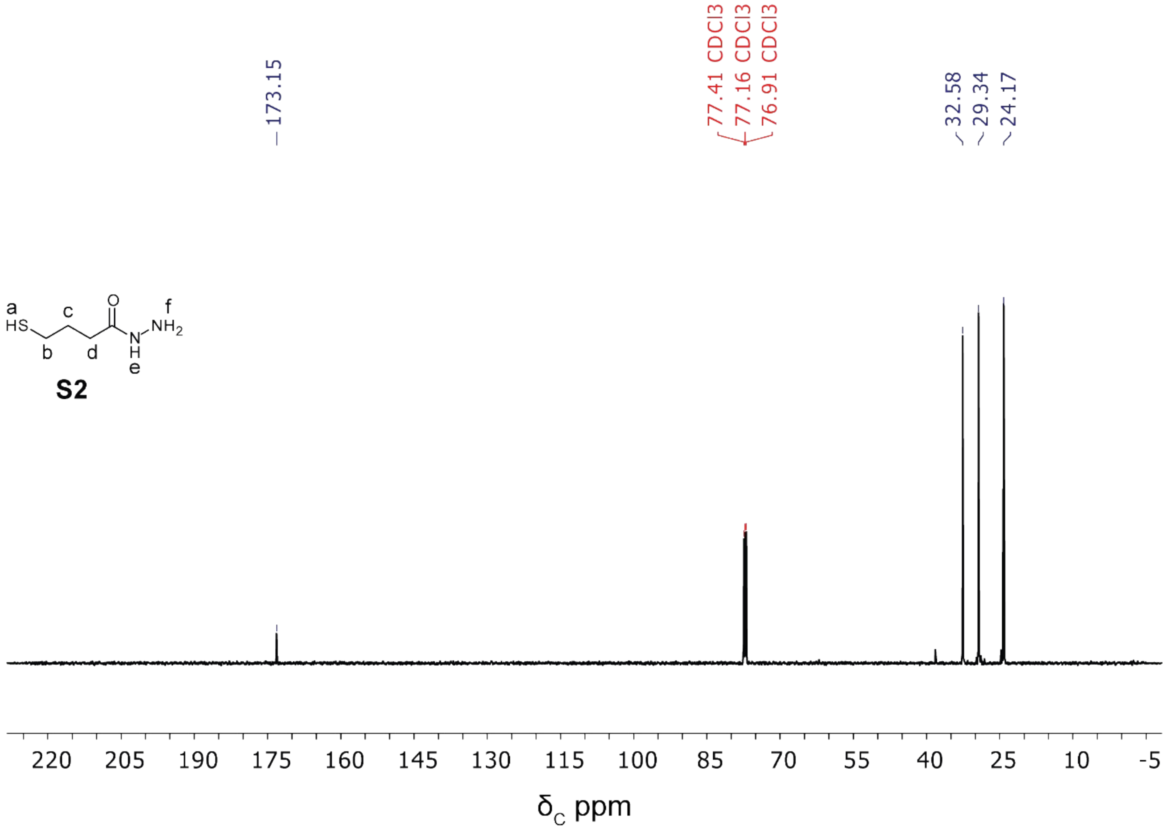
173.15
77.41 CDCl3
77.16 CDCl3
76.91 CDCl3
32.58
29.34
24.17
S2
δ_C ppm

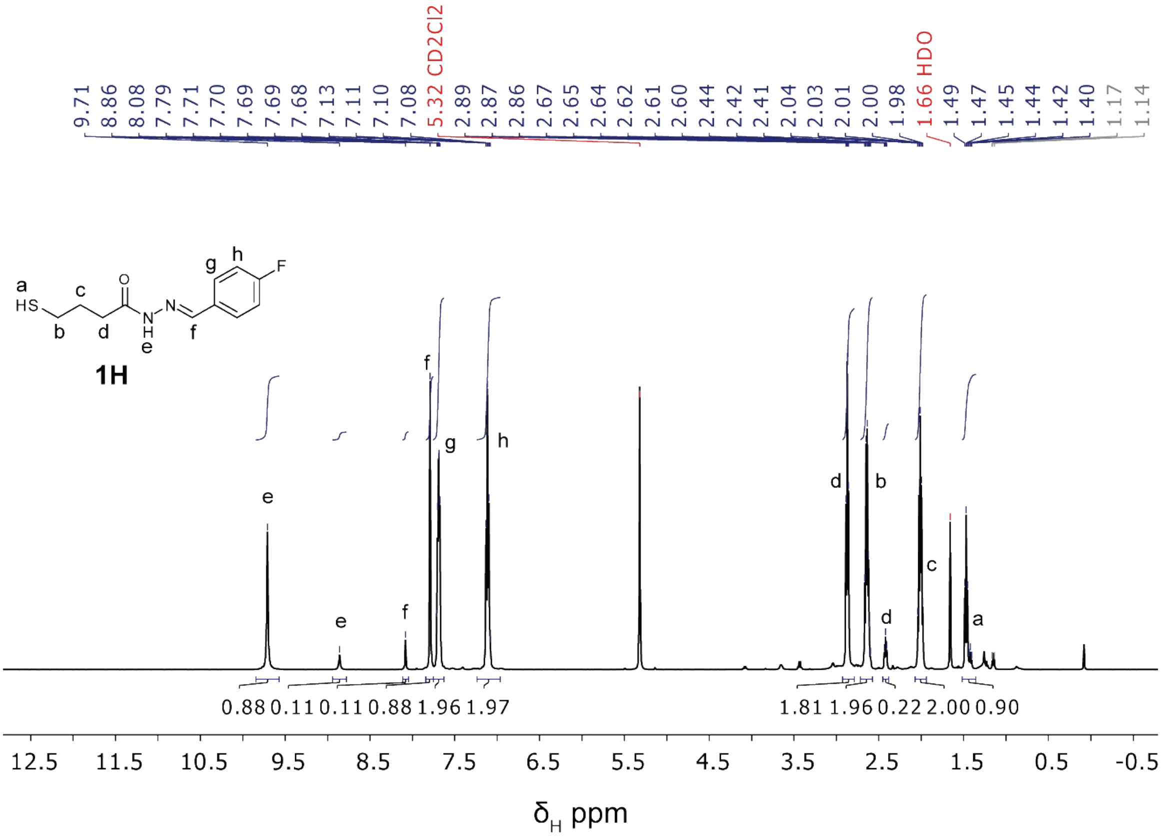
9.71
8.86
8.08
7.79
7.71
7.70
7.69
7.69
7.68
7.13
7.11
7.10
7.08
5.32 CD2Cl2
2.89
2.87
2.86
2.67
2.65
2.64
2.62
2.61
2.60
2.44
2.42
2.41
2.04
2.03
2.01
2.00
1.98
1.66 HDO
1.49
1.47
1.45
1.44
1.42
1.40
1.17
1.14
1H
0.88 0.11 0.11 0.88 1.96 1.97
1.81 1.96 0.22 2.00 0.90
12.5 11.5 10.5 9.5 8.5 7.5 6.5 5.5 4.5 3.5 2.5 1.5 0.5 -0.5
δH ppm

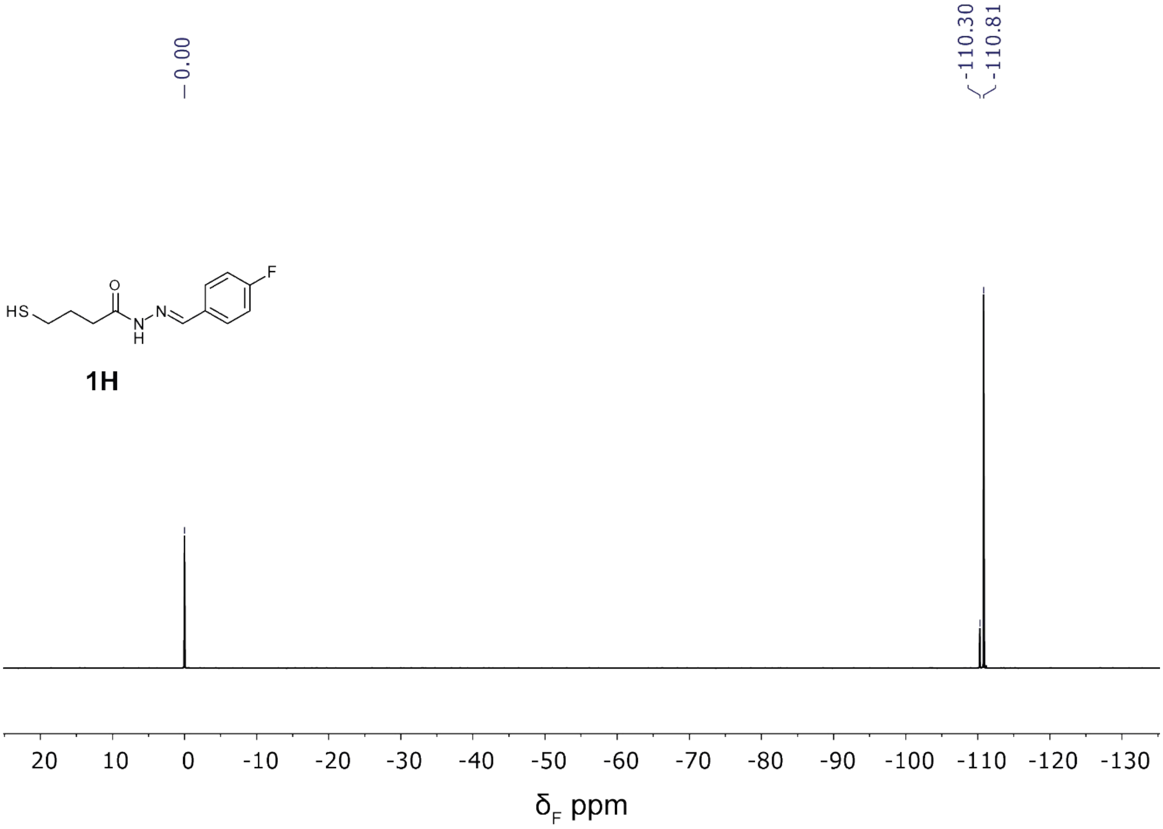
0.00
-110.30
-110.81
1H
20 10 0 -10 -20 -30 -40 -50 -60 -70 -80 -90 -100 -110 -120 -130
δF ppm

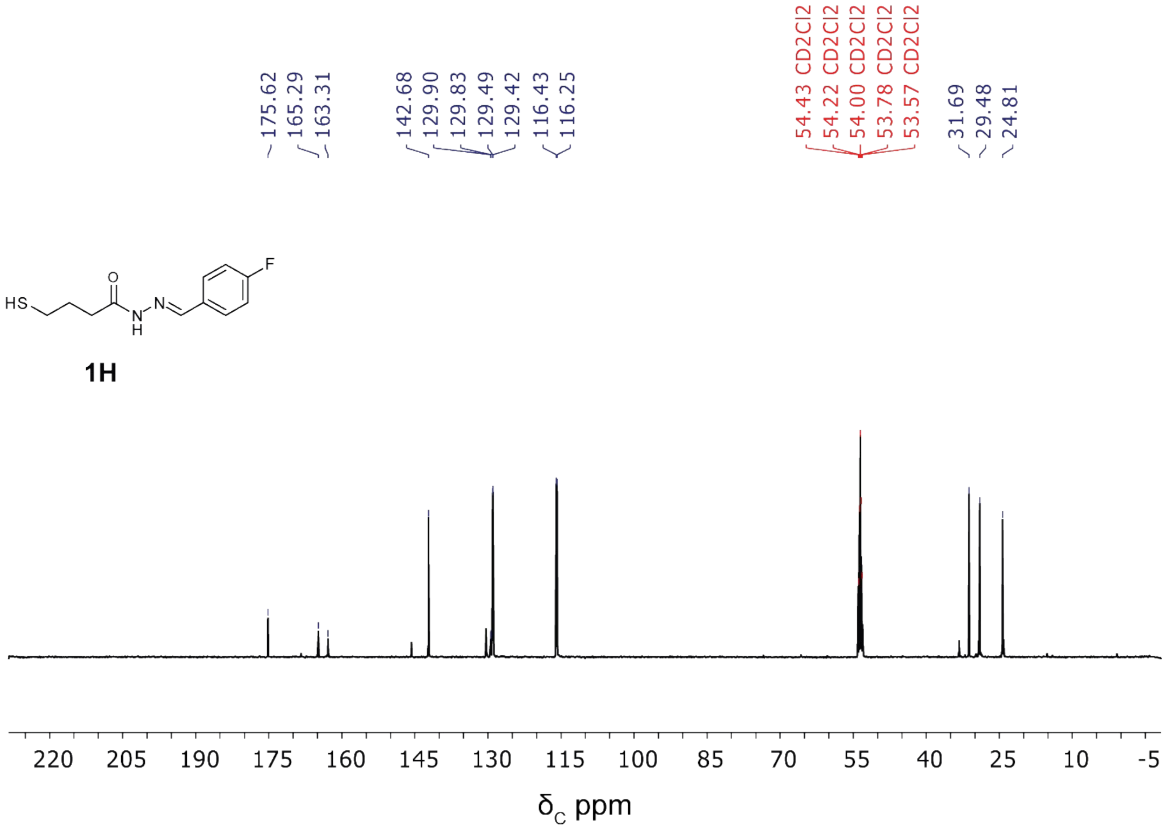

175.62
165.29
163.31
142.68
129.90
129.83
129.49
129.42
116.43
116.25
54.43 CD2Cl2
54.22 CD2Cl2
54.00 CD2Cl2
53.78 CD2Cl2
53.57 CD2Cl2
31.69
29.48
24.81
HS
O
N
H
N
F
220 205 190 175 160 145 130 115 100 85 70 55 40 25 10 -5
δC ppm

1H

9. Size distributions and representative TEM images for nanoparticle samples

AuNP-**1**

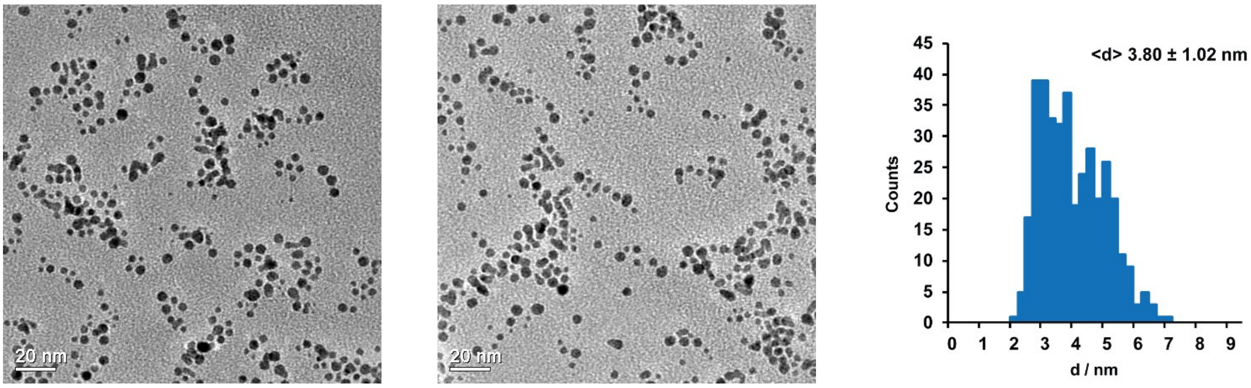

AuNP-**1**$_{0.7}$**5**$_{0.3}$

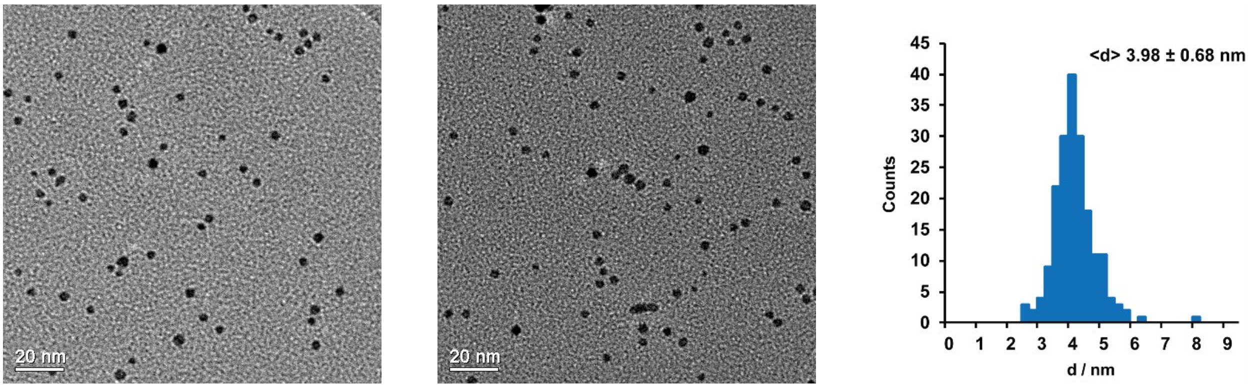

AuNP-**1**$_{0.6}$**5**$_{0.4}$

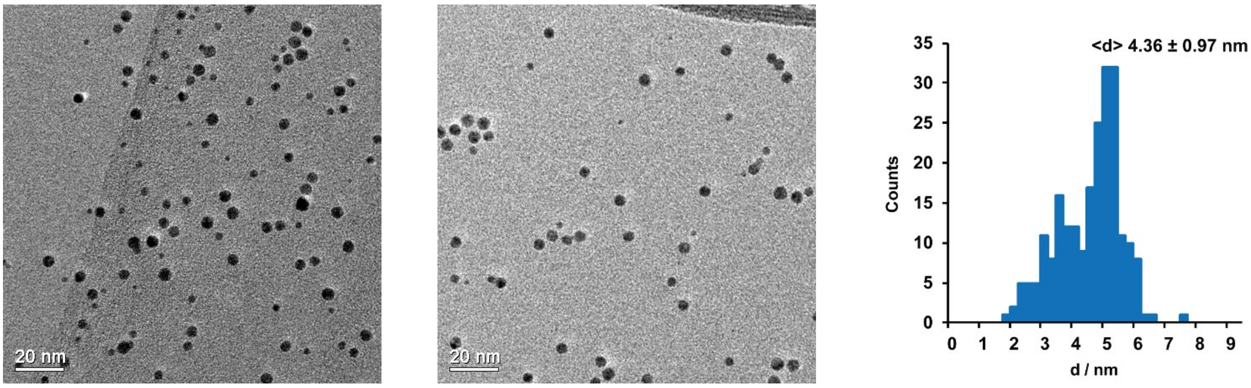

AuNP-**1**$_{0.5}$**5**$_{0.5}$

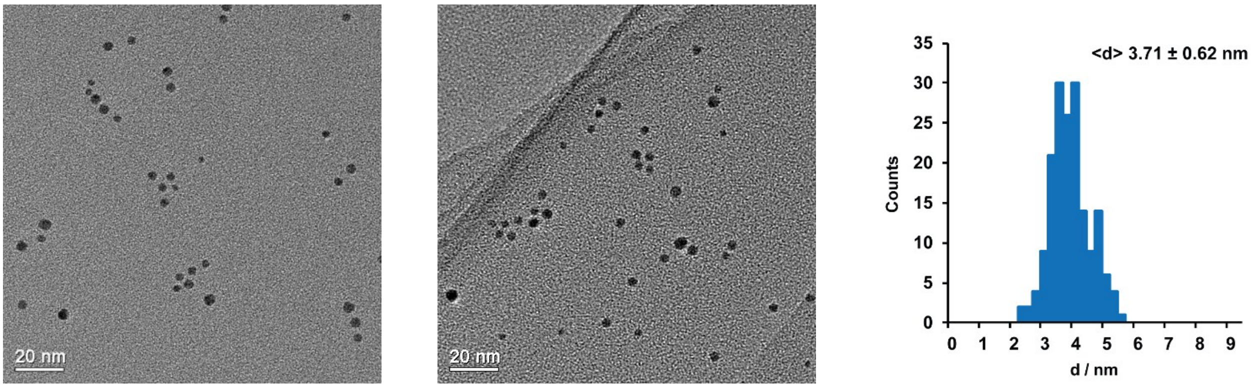

AuNP-**1**$_{0.4}$**5**$_{0.6}$

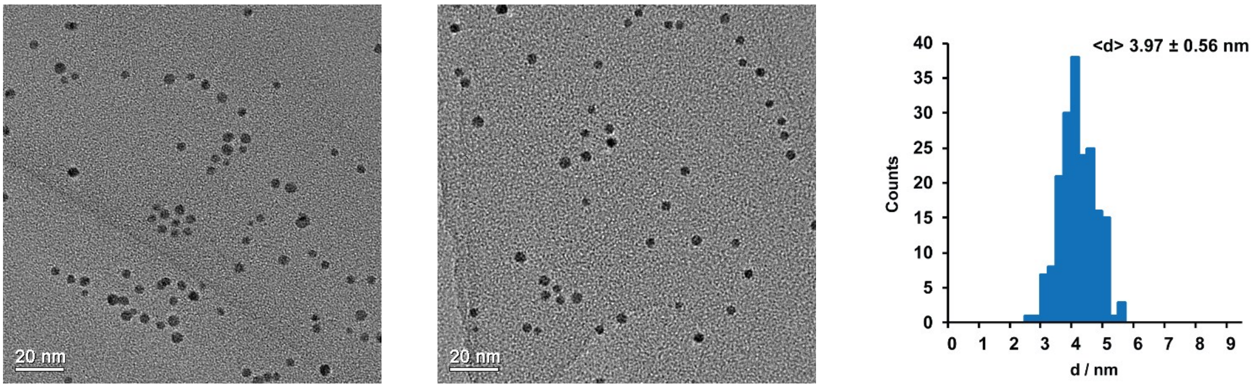

AuNP-**1**$_{0.3}$**5**$_{0.7}$

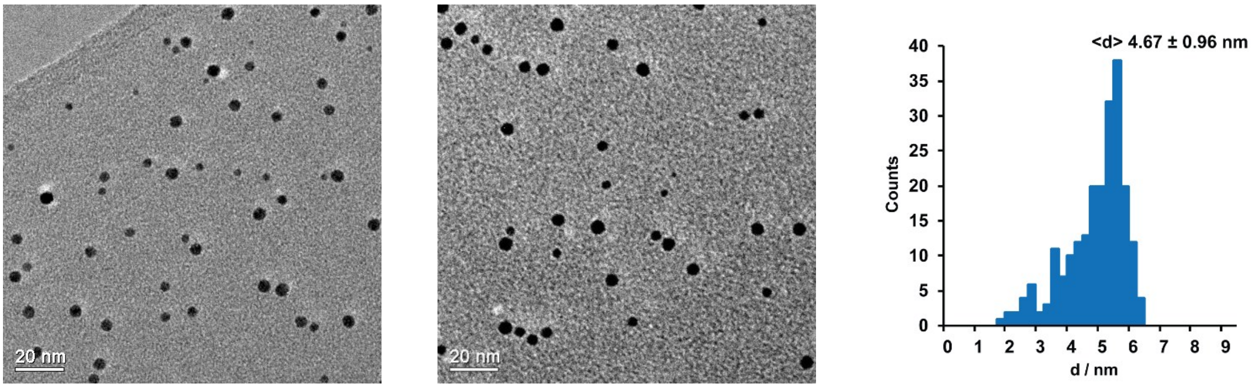

AuNP-**1**$_{0.2}$**5**$_{0.8}$

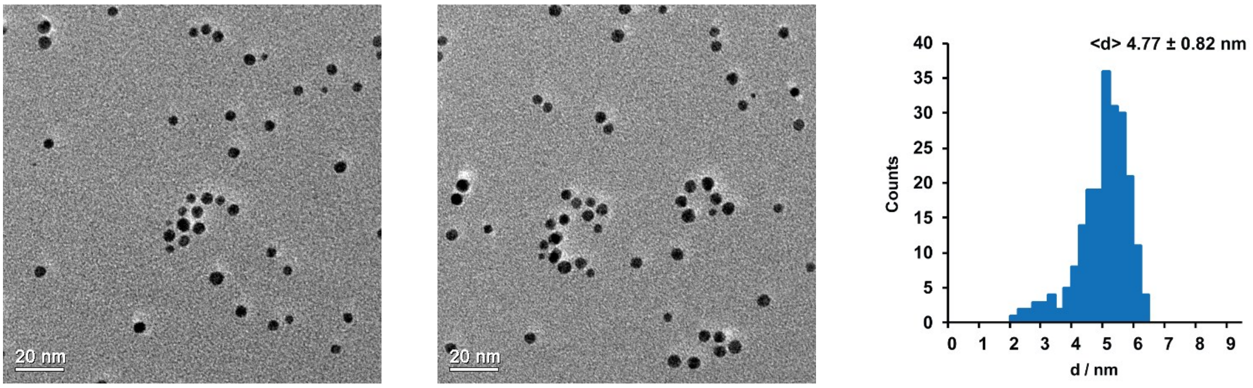

AuNP-**1**$_{0.1}$**5**$_{0.9}$-a

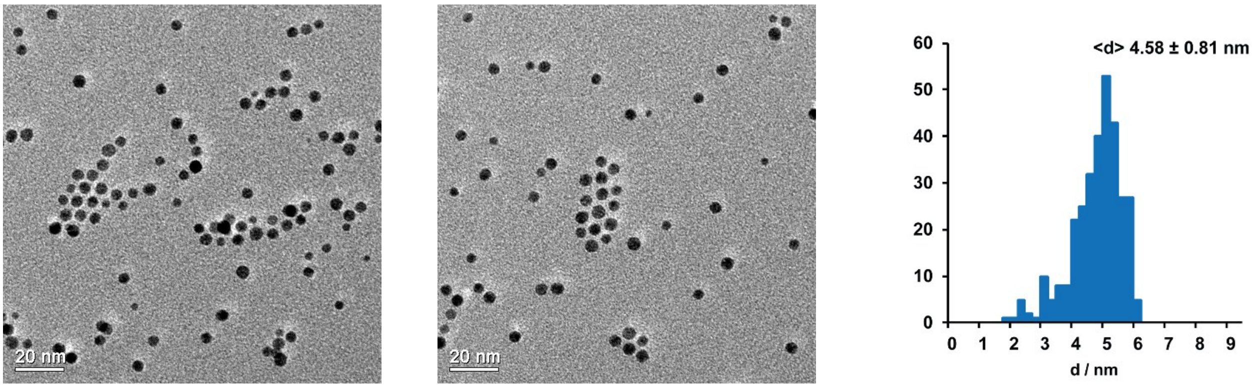

AuNP-$\mathbf{1}_{0.1}\mathbf{5}_{0.9}$-b

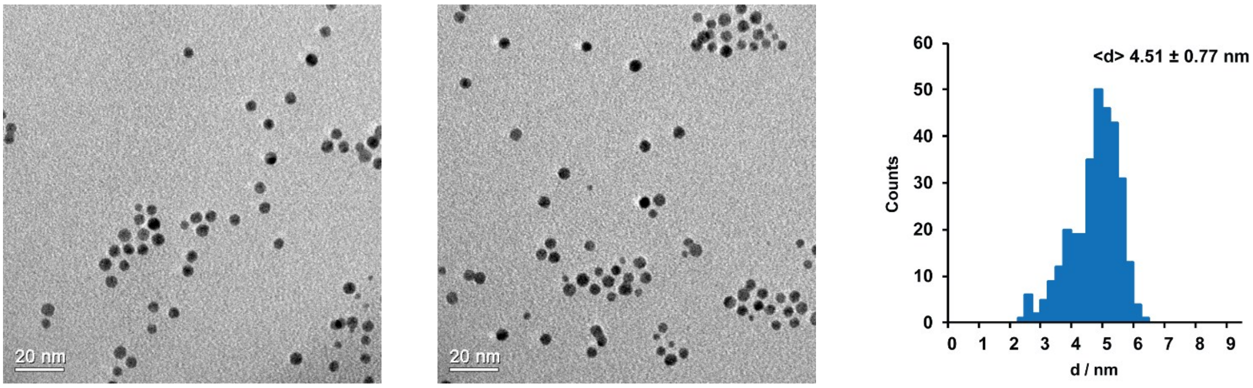

AuNP-$\mathbf{1}_{0.1}\mathbf{5}_{0.9}$-c

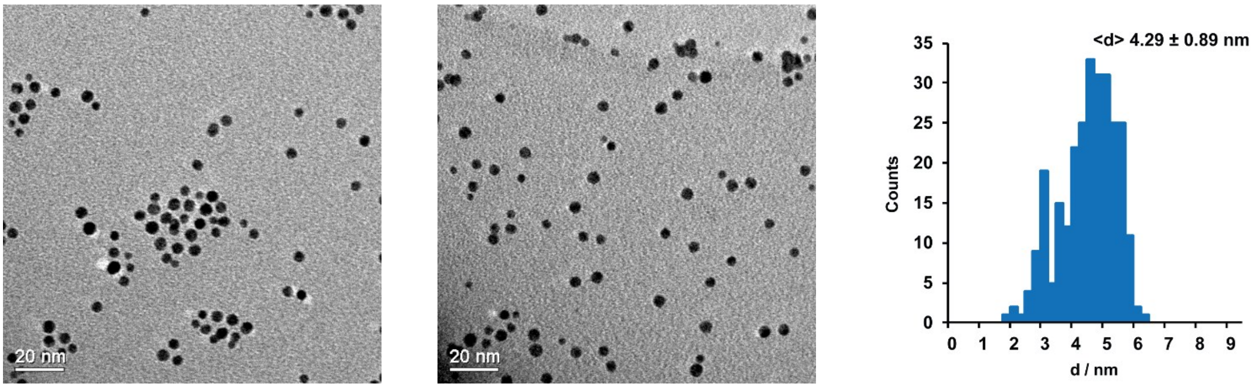

AuNP-**5**

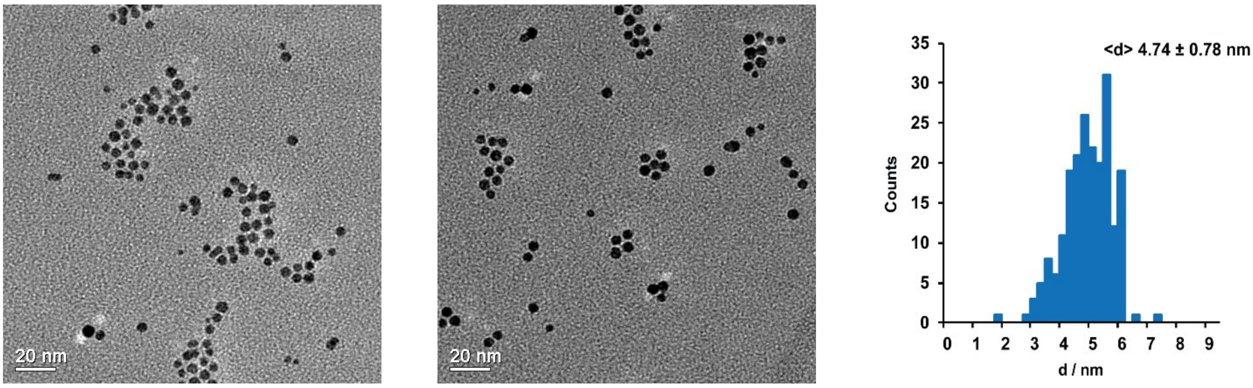

AuNP-**1**$_{0.9}$**6**$_{0.1}$

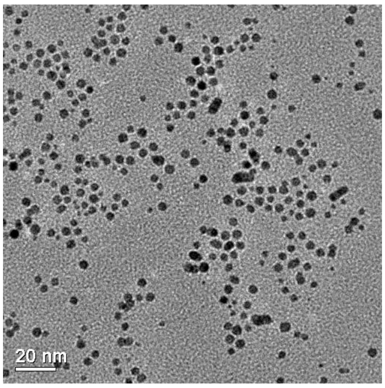

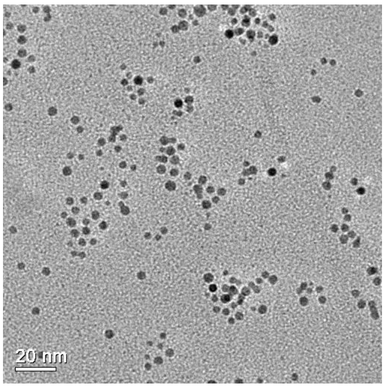

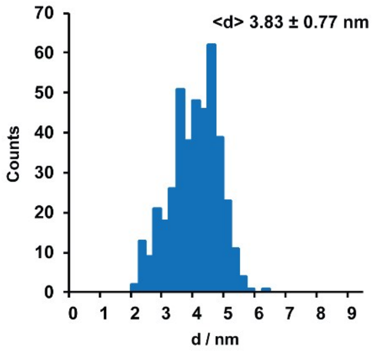

AuNP-**1**$_{0.8}$**6**$_{0.2}$

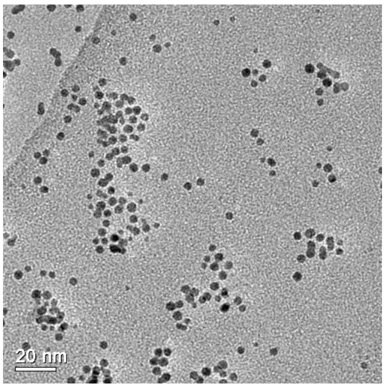

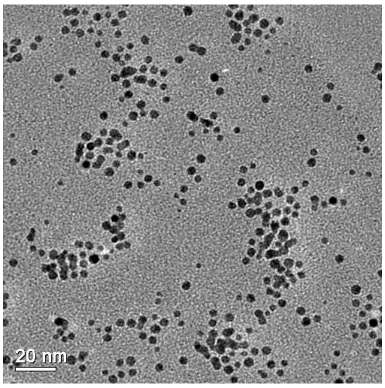

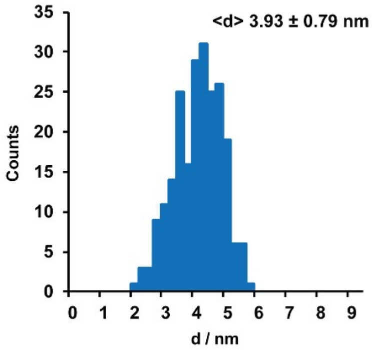

AuNP-**1**$_{0.7}$**6**$_{0.3}$

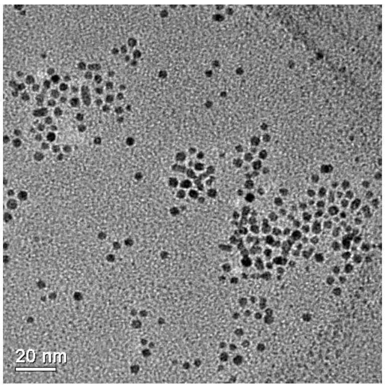

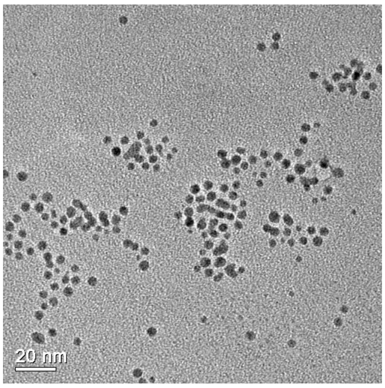

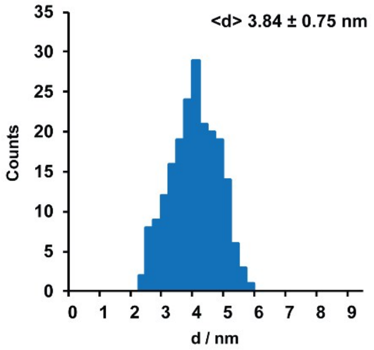

AuNP-**1**$_{0.6}$**6**$_{0.4}$

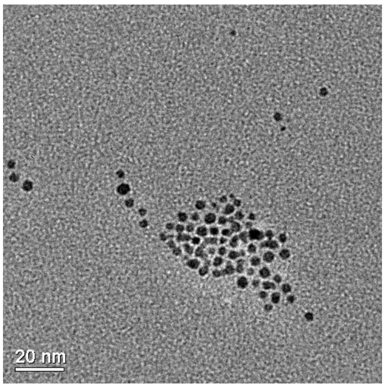

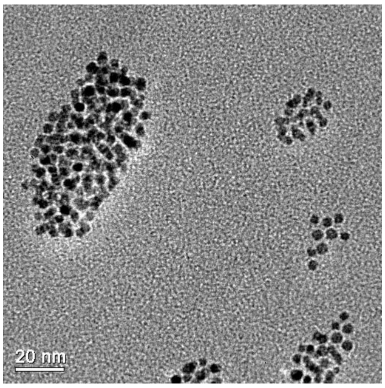

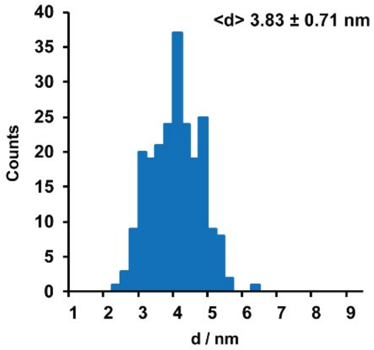

AuNP-$\mathbf{1}_{0.5}\mathbf{6}_{0.5}$-a

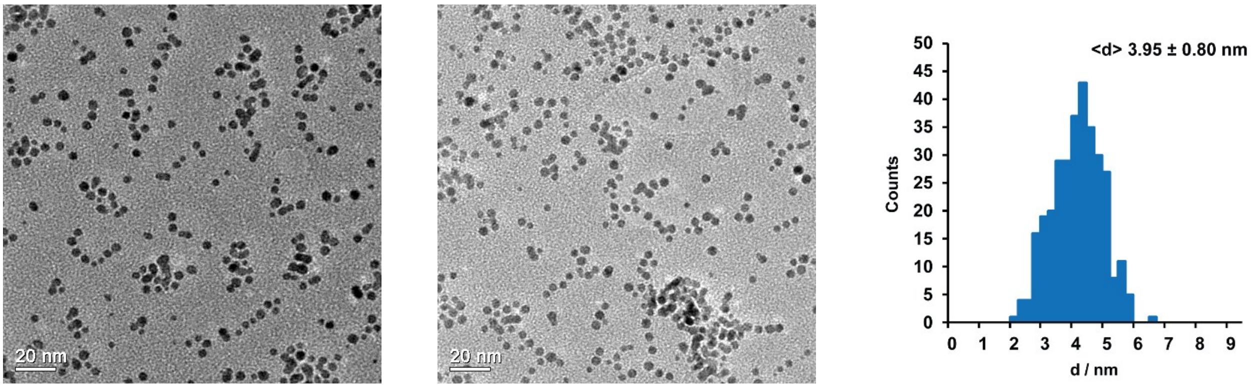

AuNP-$\mathbf{1}_{0.5}\mathbf{6}_{0.5}$-b

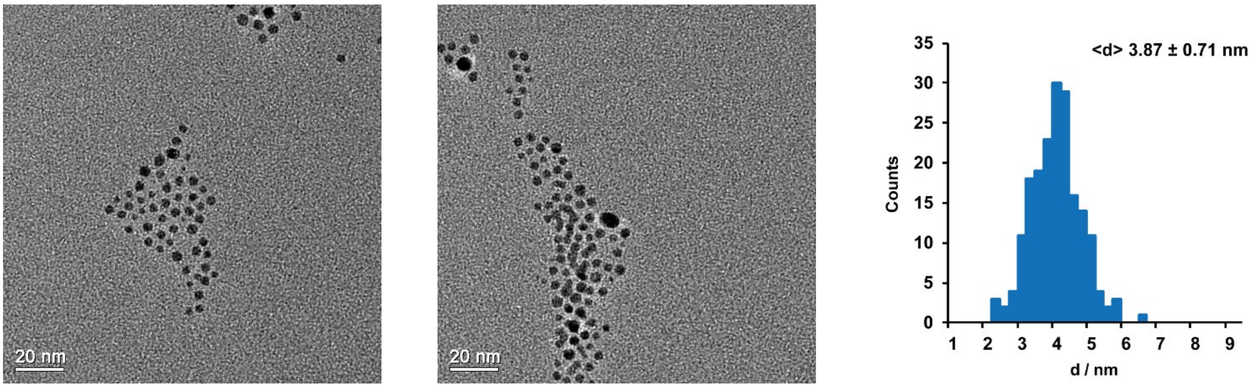

AuNP-$\mathbf{1}_{0.4}\mathbf{6}_{0.6}$

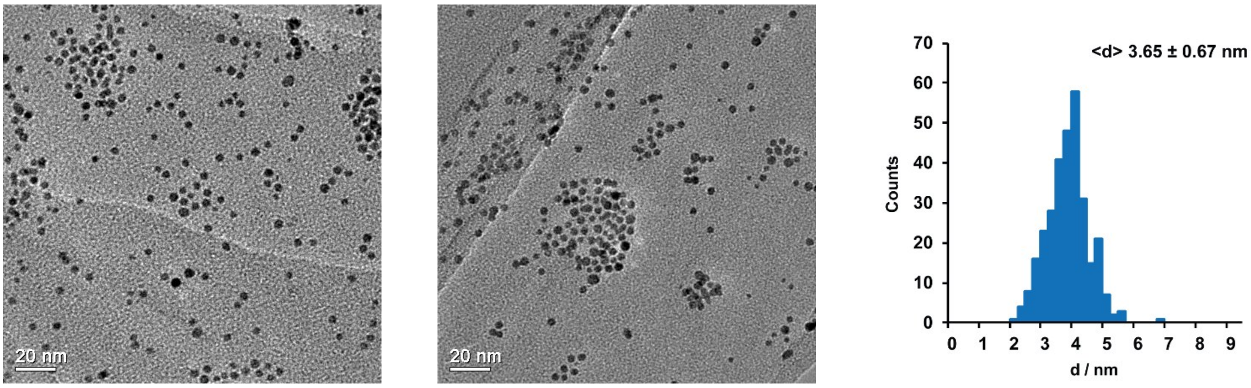

AuNP-$\mathbf{1}_{0.3}\mathbf{6}_{0.7}$

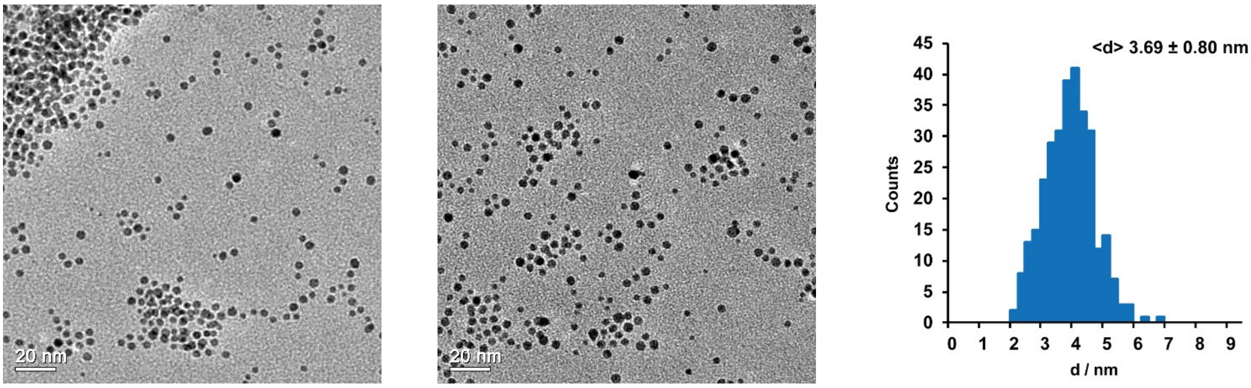

AuNP-**1**$_{0.2}$**6**$_{0.8}$

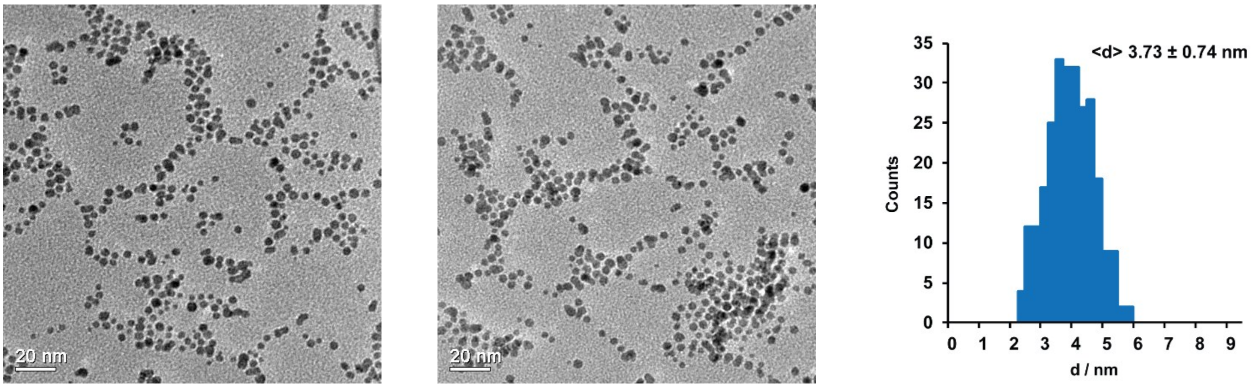

AuNP-**1**$_{0.1}$**6**$_{0.9}$

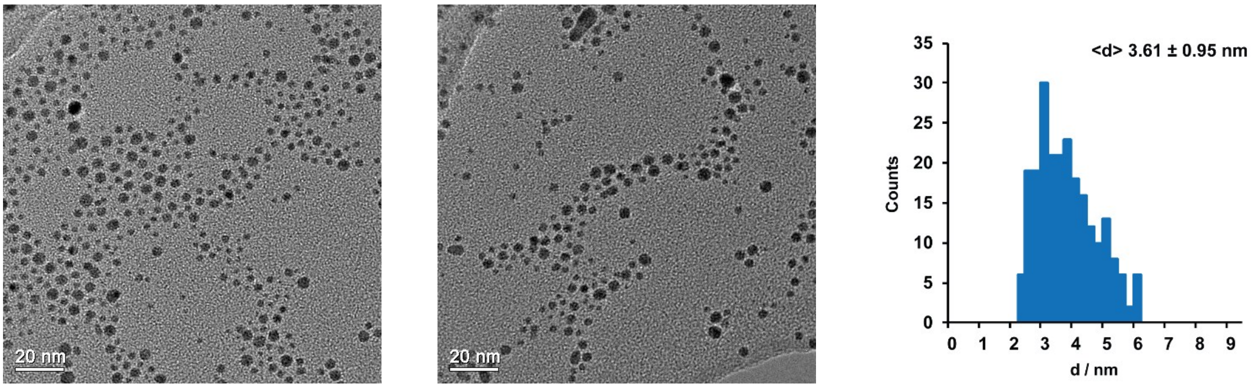

AuNP-**6**

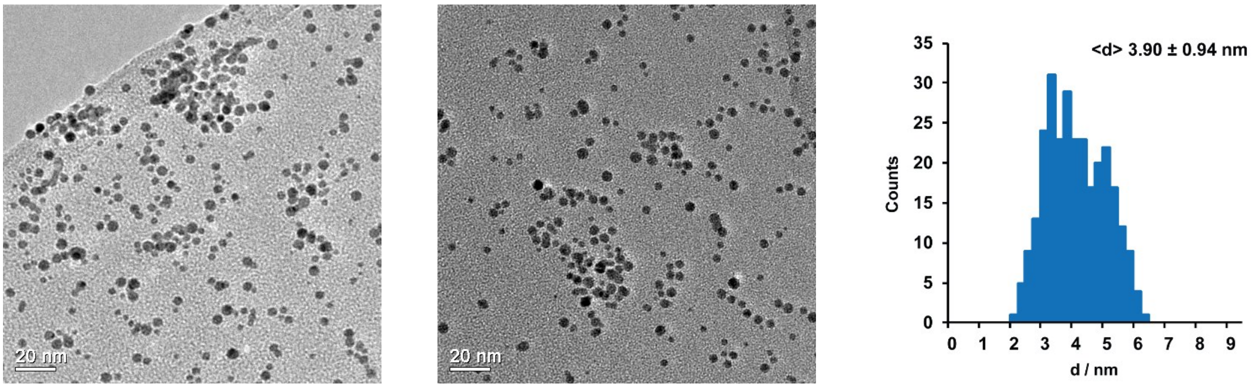

Comment on size distributions

Given the relatively large size dispersity of the starting nanoparticle sample AuNP-**1**, care must be taken not to over-interpret any apparent changes in the mean size calculated by measuring ca. 200 particles by TEM image analysis. However, it is noteworthy that the disperisty of all samples produced by dynamic covalent exchange is significantly narrower (ranging ca. 14–22%) than that for the starting point AuNP-**1** (27%). This narrowing of the size distribution – and consequent small variation in nanoparticle mean size – is most likely an outcome of unequal experimental losses of very large or very small particles during isolation and purification of each sample. Owing to the markedly differing solubiltiy properties for each sample, the purification procedure (solvents used, number of washes required) was slightly different in each case. Purification was particularly challenging for samples with small ratios of ligands **1**:**5**; in gerenal these smaples exhibited excellent solubility in a wide range of solvents, and the washing supernatant was at times observed to have a feint colour indicating unavoidable losses of some sub-population of particles.

It is important to note that the dynamic covalent exchange process is independent of the underlying nanomaterial, and does not require any careful optimization of nanoparticle size distribution to achieve the significant changes in nanoparticle physicochemical properties reported.

10. Supplementary references

1. F. della Sala and E. R. Kay, *Angew. Chem. Int. Ed.*, 2015, **54**, 4187-4191.
2. N. Zheng, J. Fan and G. D. Stucky, *J. Am. Chem. Soc.*, 2006, **128**, 6550-6551.
3. J. Song, D. Kim and D. Lee, *Langmuir*, 2011, **27**, 13854-13860.
4. F. Rastrelli, S. Jha and F. Mancin, *J. Am. Chem. Soc.*, 2009, **131**, 14222-14224.
5. (a) Y. Song, T. Huang and R. W. Murray, *J. Am. Chem. Soc.*, 2003, **125**, 11694-11701; (b) A. Caragheorgheopol and V. Chechik, *Phys. Chem. Chem. Phys.*, 2008, **10**, 5029-5041.
6. V. G. Machado, R. I. Stock and C. Reichardt, *Chem. Rev.*, 2014, **114**, 10429-10475.
7. D. R. Lide, ed., *CRC Handbook of Chemistry and Physics*, CRC Press LLC, London, 2004.
8. J. P. Cerón-Carrasco, D. Jacquemin, C. Laurence, A. Planchat, C. Reichardt and K. Sraïdi, *J. Phys. Org. Chem.*, 2014, **27**, 512-518.
9. J. L. M. Abboud and R. Notario, *Pure Appl. Chem.*, 1999, **71**, 645-718.
10. V. A. Bloomfield and R. K. Dewan, *J. Phys. Chem.*, 1971, **75**, 3113-3119.
11. W. Heller, *J. Phys. Chem.*, 1965, **69**, 1123-1129.
12. A. Jouyban, S. Soltanpour and H. K. Chan, *Int. J. Pharm.*, 2004, **269**, 353-360.
